# Polymer nanocomposites with hybrid nanoparticles: architecture–process–structure–property–performance perspectives

**DOI:** 10.1039/d6ra02849g

**Published:** 2026-08-14

**Authors:** Tasnim Alam Subah, Hasan Tasnim Salehin, Rakibul Hasan Rafi, Md. Fozle Rabbi, Sayem Kawsar, Sumaiya Khan, Shahla Rahman, Sifat Kawsar, Md Zillur Rahman

**Affiliations:** a Department of Chemical Engineering, Bangladesh University of Engineering and Technology Dhaka Bangladesh; b Department of Computer Science and Engineering, Khulna University of Engineering and Technology Khulna Bangladesh; c Department of Meat Science and Technology, Bangladesh Agricultural University Mymensingh Bangladesh; d Department of Mechanical Engineering, Ahsanullah University of Science and Technology Dhaka Bangladesh md.zillur.rahman.phd@gmail.com

## Abstract

Hybrid polymer nanocomposites have become central to the development of lightweight, high-performance, and sustainable materials in response to growing demands from electrification, advanced manufacturing, and multifunctional device technologies. In this context, hybrid nanoparticles, single nanoscale entities integrating two or more distinct nanophases, offer a timely and powerful route to overcoming the intrinsic property trade-offs of single-filler systems by enabling synergistic interactions and programmable interfacial behavior. This review critically examines polymer nanocomposites reinforced with hybrid nanoparticles, covering key hybrid nanoparticle architectures, including core–shell, Janus, heterodimer, decorated, and yolk–shell systems, as well as deliberately combined hybrid nanofiller systems, representative polymer matrices (thermoplastics, thermosets, and biopolymers), and major application domains spanning aerospace, construction, electronics, energy, biomedical, and environmental technologies. The field is structured using an architecture–process–property–application framework that links hybrid nanoparticle design, dispersion, and interphase engineering to macroscopic mechanical, thermal, electrical, optical, barrier, flame-retardant, and smart/stimuli-responsive functionalities. Synthesized evidence across the literature demonstrates that well-designed hybrid nanoparticle systems can simultaneously enhance stiffness and toughness, thermal stability and transport, electrical and dielectric performance, and multifunctionality at lower filler loadings than single-component counterparts. However, the review also highlights persistent challenges that limit translation, including reproducible dispersion at industrially relevant scales, incomplete structure–property correlations for complex hybrid architectures, inconsistent experimental reporting, processing-induced variability, and unresolved life-cycle, health, and safety considerations. Finally, concrete future directions are identified, emphasizing the need for standardized descriptors and benchmarking protocols, scalable, energy-efficient manufacturing routes, integration of in-line and multiscale characterization with processing, and adoption of sustainability- and safety-by-design strategies to enable the reliable transition of hybrid nanoparticle–polymer nanocomposites from laboratory concepts to real-world applications.

## Abstract 2

Hybrid nanoparticle architectures and interphase control enable polymer nanocomposites with synergistic mechanical, thermal, electrical, barrier, and smart performance at lower filler loadings while highlighting scale-up and sustainability barriers.

## Graphical abstract

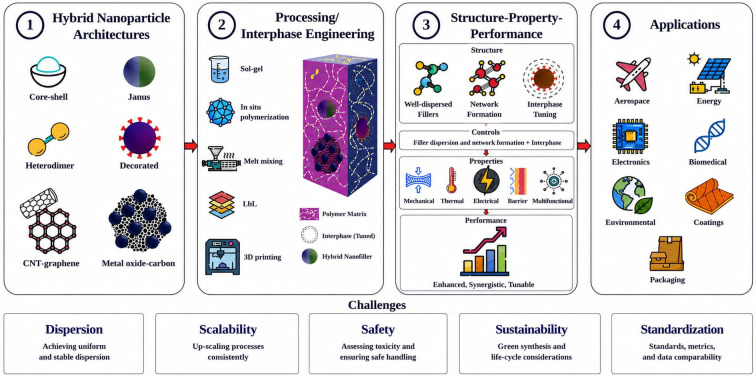

## 1. Introduction

Polymer nanocomposites have emerged as a versatile class of multifunctional materials with properties that surpass those of conventional polymers and micro-scale composites. These materials consist of a polymer matrix reinforced with nanoscale fillers, such as nanoparticles, nanoclusters, or nanoplatelets, having at least one dimension in the 1–100 nm range. Owing to their enhanced electrical, mechanical, thermal, and optical performance,^1^ polymer nanocomposites are increasingly employed in applications ranging from biomedicine to aerospace, where high performance, low weight, and tunable functionality are essential.^2^ Global trends in electrification, lightweight transportation, and circular manufacturing further intensify demand for these advanced materials, while advances in additive manufacturing and data-driven materials design accelerate their formulation, processing, and integration into functional devices.^3,4^ To further expand their performance envelope, recent research has increasingly focused on hybrid nanoparticles as tunable fillers that can tailor interfacial interactions and enable multifunctionality within polymer matrices.

Hybrid nanoparticles, defined as single particles that integrate two or more distinct nanophases, are particularly attractive because they combine, couple, or synergistically amplify the intrinsic properties of their constituents, enabling multifunctionality beyond that attainable with single-component fillers. Representative architectures include core–shell, Janus, heterodimer, yolk–shell, and branched or surface-functionalized structures.^5–7^ The current literature highlights the decisive roles of controlled synthesis, morphology, and interfacial structure in determining the optical, electronic, catalytic, and magnetic responses of hybrid systems, including semiconductor-metal and plasmonic heterostructures relevant to catalysis, optoelectronics, and sensing.^8^

**Fig. 1:**
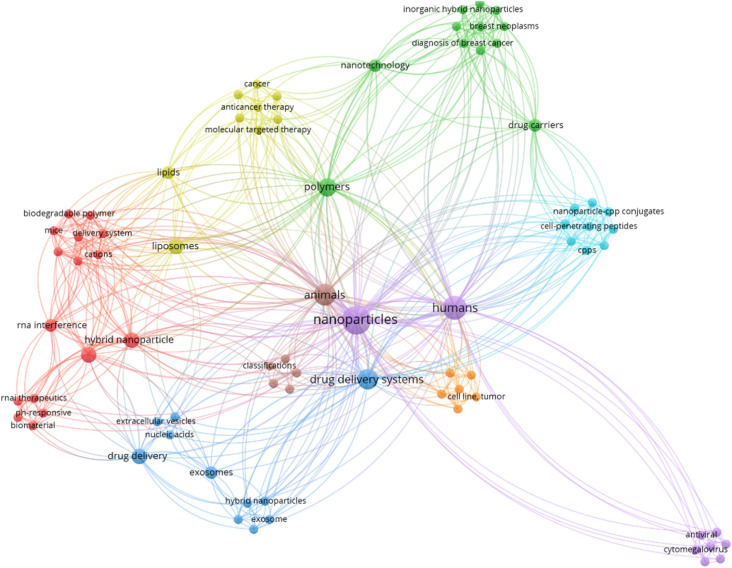
Co-occurrence of “hybrid nanoparticles” in publications from the last 5 years collected from PubMed.

When embedded in polymer matrices with appropriate dispersion and interfacial engineering, hybrid nanoparticles can simultaneously enhance stiffness, strength, and fracture resistance, increase thermal stability and heat transport, tailor dielectric response, and introduce functions such as magnetism, antimicrobial activity, degradability, and optoelectronic behavior.^9–11^ In addition, the architecture and surface chemistry of hybrid fillers can be designed for stimuli-responsive behavior to temperature, pH, light, electric fields, or magnetic fields, enabling smart sensors, actuators, and reconfigurable or self-healing devices.^12,13^ These capabilities are already reflected in emerging applications in automotive, biomedical, packaging, electronics, and aerospace technologies, where durability, reliability, and multifunctional performance are significant.^14^ A five-year co-occurrence analysis of the term “hybrid nanoparticles” in PubMed-indexed publications illustrates the rapid growth and increasing cross-disciplinary character of this field (Fig. 1), underscoring the need for integrative reviews.

Sustainability has become a central design constraint for hybrid nanomaterials and their integration into polymer nanocomposites. Green routes to metal, metal oxide, and organic-inorganic hybrids, including plant- and microbe-mediated syntheses, are being developed to reduce solvent use, energy demand, and toxicity, while improving biocompatibility for biomedical and environmental applications.^15^ Recent reviews describe biosynthetic strategies that use plant extracts, microorganisms, and biopolymers as reducing, capping, and templating agents, and emphasize their potential to replace conventional high-temperature or solvent-intensive processes in scalable hybrid nanoparticle manufacture.^16–19^ In parallel, additive manufacturing enables complex geometries and on-demand components based on polymer nanocomposite feedstocks, while machine-learning methods are increasingly applied to accelerate formulation screening, property prediction, and process optimization for both conventional processing and 3D printing of polymer composites.^3,4^

Despite substantial progress, several gaps continue to limit the translation of hybrid nanoparticle–polymer nanocomposites. Reproducible dispersion at industrially relevant loadings, durable interfacial bonding across chemically diverse polymer matrices, and robust structure–property correlations for complex hybrid morphologies remain unresolved. In addition, standardized reporting of particle architecture, surface chemistry, interphase characteristics, and processing history is still inconsistent, while scale-up introduces batch variability, cost, energy demand, solvent use, and safety-related concerns. For coatings and barrier systems, clearer links among nano-architecture, coating microstructure, and failure mechanisms under realistic service conditions are also needed.^3,8,20^ Although recent reviews have addressed polymer nanocomposites, interphase effects, additive manufacturing, high-filler-content systems, hybrid nanostructures, and hybrid filler synergy, most are organized around a specific processing route, filler class, interfacial topic, matrix family, or application domain. Table 1 clarifies how the present review differs from these studies.

**Table 1:** Positioning of the present review relative to selected recent reviews and perspectives

Selected recent review/perspective	Main focus	Main limitation relative to the present review	Distinction of the present review
Zhang *et al.*^3^	Organic/inorganic nanocomposite fabrication, from conventional synthesis to additive manufacturing	Strong process focus, but not specifically organized around hybrid nanoparticles as polymer nanofillers across architecture, interphase, properties, applications, and translation barriers	Uses an architecture–process–structure–property–performance framework centered on hybrid nanoparticles and hybrid nanofiller systems in polymer matrices
Ben-Shahar *et al.*^8^	Colloidal semiconductor–metal hybrid nanostructures, including synthesis, coupled properties, and emerging applications	Comprehensive for hybrid nanostructures, but not focused on polymer matrices, nanocomposite processing, or polymer-specific property development	Transfers hybrid nanoparticle architecture concepts into polymer nanocomposite design, processing, interphase control, and multifunctional performance
Rayhan *et al.*^21^	Additive manufacturing of nanocomposite materials and applications	Process-centered review with limited coverage of hybrid nanoparticle taxonomy, interphase formation, property trade-offs, safety, and sustainability	Places additive manufacturing within a broader framework connecting hybrid architecture, dispersion, interphase, properties, applications, and translation barriers
Huang *et al.*^20^	Interphase construction and control in polymer nanocomposites	Interphase-focused perspective, with limited comparison of hybrid nanoparticle architectures, manufacturing routes, application sectors, and regulatory or sustainability issues	Integrates interphase mechanisms with hybrid nanoparticle architecture, processing history, structure–property relationships, and end-use requirements
Kumar *et al.*^22^	Practical relevance, successes, and bottlenecks of polymer nanocomposites	Broad application-oriented perspective, but not focused on hybrid nanoparticle architectures or hybrid nanofiller systems	Extends application-oriented polymer nanocomposite discussion to hybrid nanoparticles, multifunctionality, scalability, and reporting needs
Müller *et al.*^23^	Processing, properties, nanocoating, and applications of polymer nanocomposites in packaging, automotive, and solar-energy fields	Broad polymer nanocomposite review, with limited emphasis on hybrid nanoparticle architecture and architecture-controlled multifunctionality	Focuses specifically on hybrid nanoparticles and deliberately combined hybrid nanofiller systems in polymer matrices
Harito *et al.*^24^	High-filler-content polymer nanocomposites, including synthesis, structures, properties, and applications	Emphasis on high filler loading and structural reinforcement rather than low-loading hybrid architecture, interphase programming, and multifunctional property balance	Examines how hybrid architectures improve dispersion, interphase formation, percolation, and multifunctionality at practical filler contents
Paszkiewicz *et al.*^25^	Functional polymer hybrid nanocomposites based mainly on polyolefin matrices	Valuable discussion of hybrid nanofillers in polyolefins, but limited matrix scope and less attention to hybrid nanoparticle taxonomy, safety, sustainability, and broad applications	Broadens the discussion across thermoplastics, thermosets, biopolymers, hybrid nanoparticle architectures, manufacturing routes, properties, applications, and translation issues
Afolabi and Ndou^26^	Synergy of hybrid fillers in composite and nanocomposite materials	Focuses on hybrid filler synergy and mechanical or functional improvements, but does not systematically connect hybrid nanoparticle architecture, polymer interphase, process history, and application-specific performance	Provides an integrated architecture–process–structure–property–performance synthesis for hybrid nanoparticle–polymer nanocomposites

Accordingly, this review makes three main contributions. First, it provides a structured classification of hybrid nanoparticle and hybrid nanofiller architectures, including core–shell, multi-shell, Janus, heterodimer, decorated, yolk–shell, graphene-CNT, graphene-metal oxide, and ceramic-carbon systems, and relates these architectures to interphase formation and property development. Second, it connects synthesis and processing routes with dispersion, interfacial chemistry, structure formation, and mechanical, thermal, electrical, optical, barrier, flame-retardant, and stimuli-responsive performance. Third, it evaluates application opportunities and translation barriers, with attention to scalability, reporting standards, toxicity, sustainability, and commercialization. This organization distinguishes the present review through an integrated architecture–process–structure–property–performance framework for hybrid nanoparticle–polymer nanocomposites.

The remainder of the manuscript is organized as follows. Section 2 defines the key materials concepts and discusses polymer matrices, nanoparticle classes, hybrid nanoparticle architectures, and interfacial mechanisms. Section 3 compares manufacturing routes, including sol–gel synthesis, *in situ* polymerization, melt and solution processing, layer-by-layer assembly, additive manufacturing, microfluidics, atomic layer deposition, and click functionalization, with attention to cost, energy demand, scalability, and environmental burden. Section 4 evaluates the effects of hybrid architectures on mechanical, thermal, electrical, optical, bioactive, magnetic, barrier, flame-retardant, and stimuli-responsive properties. Section 5 links these properties to aerospace, automotive, construction, biomedical, environmental, energy, electronics, packaging, wearable, energy-harvesting, and membrane applications. Section 6 examines safety and regulatory issues. Section 7 identifies remaining limitations related to processing, characterization, economics, environmental impact, and safety. Section 8 outlines emerging research directions, and Section 9 summarizes the main conclusions.

## 2. Fundamentals of polymer nanocomposites

### 2.1. Polymer matrices as interphase-forming media in hybrid nanoparticle nanocomposites

In hybrid nanoparticle–polymer nanocomposites, the polymer matrix should be considered not only as a continuous phase but also as an interphase-forming medium that controls dispersion, stress transfer, transport pathways, and long-term stability. Matrix chemistry, chain mobility, polarity, crystallinity, cure behavior, melt viscosity, and recyclability determine whether hybrid nanoparticles remain dispersed, form percolated networks, or agglomerate into defect-rich domains.^27,28^ Therefore, the selection of thermoset, thermoplastic, or biopolymer matrices should be guided by compatibility with the hybrid nanoparticle surface, the processing route, the target property combination, and the end-of-life requirement, rather than by polymer class alone.

**Fig. 2:**
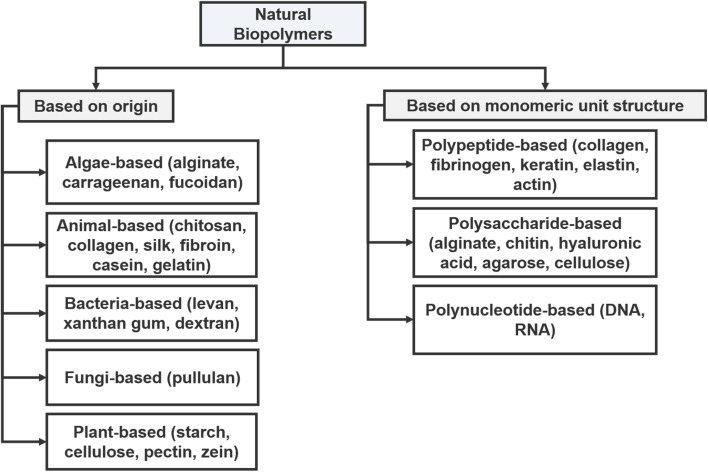
Classification of natural biopolymers based on origin and monomeric unit structure, adapted from ref. 32 and 33, under CC BY 4.0 license.

Thermoset matrices such as epoxy and polyester offer high dimensional stability, chemical resistance, and strong interfacial bonding after curing, making them suitable for structural and protective hybrid nanocomposites. However, their irreversible crosslinked networks restrict reprocessing and can make dispersion control difficult once viscosity rises during curing.^28^ Thermoplastic matrices are more compatible with melt processing and recycling, but high melt viscosity can restrict nanoparticle exfoliation, disrupt fragile hybrid architectures, and increase shear-induced filler damage during extrusion or injection molding.^28–30^ In commodity thermoplastics such as LDPE and PP, low polarity can limit adhesion with polar metal oxide or ceramic domains, requiring compatibilizers or surface-functionalized hybrid fillers to improve interphase quality.^30,31^ Biopolymer and natural-polymer-derived matrices, including polysaccharide-, protein-, and polynucleotide-based systems, offer renewable and chemically tunable platforms for hybrid nanocomposites, but their moisture sensitivity, batch variability, and thermal limitations must be considered when durable performance is required (Fig. 2).^32,33^

Thus, the critical issue is not whether the matrix is thermoset, thermoplastic, or bio-derived, but whether its chemistry and processing history can stabilize the intended hybrid nanoparticle architecture. Strong matrix-filler compatibility improves load transfer and transport continuity, while poor compatibility promotes agglomeration, weak interfacial adhesion, and inconsistent multifunctional performance.

### 2.2. Nanoparticle classes relevant to hybrid nanoparticle design

Nanoparticles are commonly defined as materials with at least one dimension in the nanometre range (∼1–100 nm), but their relevance to polymer nanocomposites depends on how their chemistry, geometry, surface functionality, and interfacial behavior interact with the polymer matrix.^34^ Filler dimensionality, such as 0D particles, 1D nanotubes or fibers, and 2D platelets or sheets, significantly affects the percolation threshold, stress transfer, barrier tortuosity, viscosity, and the formation of thermal or electrical networks.^35–38^

**Fig. 3:**
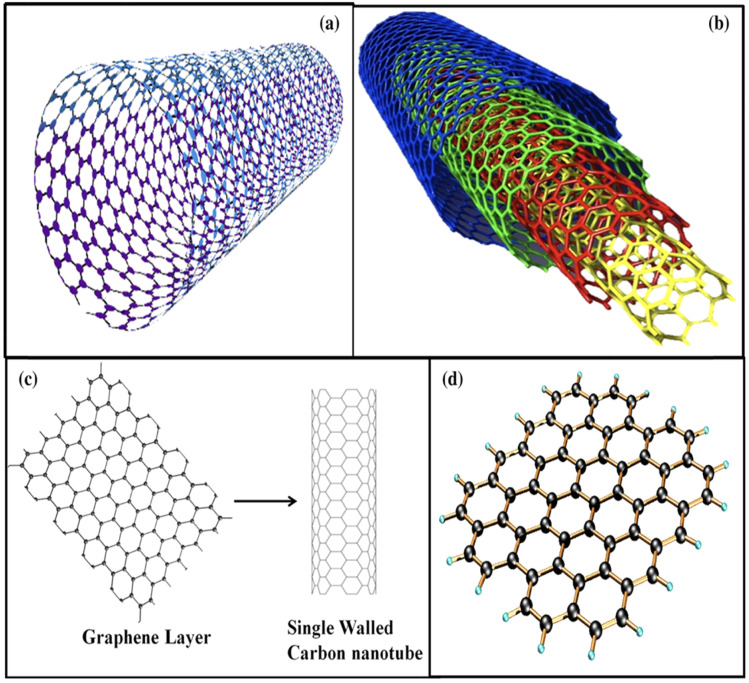
Structures of (a) SWCNTs, (b) MWCNTs, (c) graphene to SWCNT formation, and (d) graphene sheet, reproduced from ref. 46 with permission from Springer Nature, copyright 2021 (License No. 6296610885136).

Metal nanoparticles can contribute plasmonic, conductive, catalytic, or magnetic functions, but their high surface energy and tendency toward aggregation often require surface modification, encapsulation, or coupling with oxide, carbon, or polymeric domains before reliable incorporation into polymer matrices.^39–41^ Metal oxide nanoparticles such as ZnO, Fe_2_O_3_/Fe_3_O_4_, CeO_2_, TiO_2_, Al_2_O_3_, and SnO_2_ provide thermal, magnetic, photocatalytic, antimicrobial, barrier, and reinforcement functions; however, their polar surfaces often show poor compatibility with non-polar polymer matrices unless interfacial chemistry is adjusted.^42–45^ Carbon nanostructures, including CNTs and graphene-family fillers, are effective for mechanical reinforcement and electrical or thermal transport, but they frequently suffer from bundling, restacking, and processing-induced network disruption.^46^Fig. 3 illustrates how CNTs and graphene-derived sheets provide complementary 1D and 2D pathways for stress transfer, transport, and percolated network formation in hybrid polymer nanocomposites.

Hybrid nanoparticle design aims to overcome these single-filler limitations by combining complementary nanoscale components within one architecture or within a deliberately combined nanofiller system. Examples include oxide-coated metal nanoparticles for stability and interfacial control, nanoparticle-decorated graphene sheets for reduced restacking and improved multifunctionality, and CNT-graphene systems that combine 1D bridging with 2D transport pathways. At the same time, hybrid systems introduce new complications, including heteroaggregation, mismatched surface chemistries, increased viscosity, more difficult characterization, and reduced reproducibility during scale-up. Therefore, the central question for hybrid nanoparticle–polymer nanocomposites is not simply which nanoparticle class is used, but how nanoparticle architecture, surface chemistry, dimensionality, and processing route jointly determine dispersion, interphase formation, and property balance.

### 2.3. Hybrid nanoparticles in polymer nanocomposites

To maintain consistent terminology, this review distinguishes among four related but non-equivalent terms: hybrid materials, nanohybrids, hybrid nanoparticles, and hybrid nanofillers, as presented in Table 2. This distinction is important because the manuscript discusses both individual multicomponent nanoparticle architectures and combined nanofiller systems incorporated into polymer matrices.

**Table 2:** Terminology used in this review

Term	Definition and use in this review
Hybrid materials	Multicomponent materials in which organic, inorganic, or both types of components are intimately combined. This term is used for the broad material class and does not necessarily imply nanoscale size or use as a polymer filler^47,48^
Nanohybrids	Hybrid materials containing at least one nanoscale component or domain. This term is used to refer to nanoscale hybrid materials in a general sense, without restricting the discussion to individual particles or polymer reinforcement^49,50^
Hybrid nanoparticles	Individual nanoscale particles composed of two or more integrated components, domains, shells, faces, or surface-decorated regions within a single particle, such as core–shell, multi-shell, Janus, heterodimer, yolk–shell, or decorated nanoparticles^5,6,51,52^
Hybrid nanofillers	Nanoscale reinforcing or functional fillers incorporated into a polymer matrix. In this review, the term includes hybrid nanoparticles used as fillers and deliberately combined nanofiller systems, such as CNT-graphene, graphene-metal oxide, or ceramic-carbon nanofiller combinations^53–56^

In this review, hybrid materials are used as the broadest term for multicomponent hybrid systems, while nanohybrids refer to hybrid systems containing at least one nanoscale component or domain. The term hybrid nanoparticles is reserved for individual nanoscale entities in which two or more components are integrated within a single particle architecture. By contrast, hybrid nanofillers refer to nanoscale fillers after incorporation into a polymer matrix, including both hybrid nanoparticles and deliberately combined nanofiller systems. Therefore, all hybrid nanoparticles used in a polymer matrix may be considered hybrid nanofillers, but not all hybrid nanofiller systems consist of a single hybrid nanoparticle.

In polymer nanocomposites, hybridization is used to suppress nanoparticle aggregation, engineer polymer-filler interphases, improve load, charge, or heat transfer, and combine functions such as mechanical reinforcement, barrier resistance, conductivity, optical activity, magnetism, and catalytic activity that are difficult to obtain using a single nanoparticle type alone.^53,56^ From a structure–property perspective, performance gains are governed not only by the intrinsic properties of the nanofillers but also by the architecture-controlled interphase that forms around them in the polymer matrix, which can dominate the composite's effective properties when the filler size is nanoscale.^20^Table 3 summarizes the hybrid and composite material classes relevant to this review.

**Table 3:** Classification of hybrid and composite materials relevant to this review

Class	Key features	Reference
Composites	Multicomponent material comprising multiple, different (non-gaseous) phase domains, in which at least one phase is continuous (*i.e.*, matrix)	48
Nanocomposites	Composite in which at least one phase domain has at least one dimension of the order of nanometers (often <100 nm)	48
Hybrids	Intimately mixed multicomponent materials (often organic–inorganic) in which the interphase interactions/coupling are central and can yield synergistic or emergent properties beyond simple mixing; commonly discussed using class I/class II bonding	48
Nanohybrids	Hybrid materials with nanoscale building blocks/domains (order of nm), *i.e.*, hybrids that also meet the nanoscale/domain criterion, may be class I or class II depending on interphase coupling	50
Class I hybrids	Components linked primarily by weak interactions (*e.g.*, van der Waals, hydrogen bonding, weak electrostatic interactions)	57
Class II hybrids	Components linked by strong chemical interactions (*e.g.*, covalent, iono-covalent/ionic-covalent, coordinative bonding)	57
Organic–inorganic hybrids	Hybrid materials combining organic and inorganic components at nano/molecular scale; designed to couple functionalities (*e.g.*, flexibility + thermal/optical/mechanical performance); can be class I or class II	48 and 57
Inorganic–inorganic hybrids	Hybrid materials combining two different inorganic components (*e.g.*, heterostructures, core–shell, mixed oxides) where coupling at the interface yields improved mechanical/catalytic/functional performance	58
Organic–organic hybrids	Hybrid materials combining two different organic components with designed interphase coupling (often nanoscale phase separation or interpenetration); avoid treating simple miscible blends as “hybrids” unless interphase-driven synergy is demonstrated	58

Hybrid nanoparticles used in polymer nanocomposites are commonly categorized as core–shell, multi-shell, Janus, decorated, and heterodimer structures. Core–shell nanoparticles consist of an inner core surrounded by an outer shell. The shell can serve as a diffusion/thermal barrier, a compatibilizing layer, or a protective coating that mitigates sintering and aggregation, thereby improving dispersion stability and enabling more reliable control of interfacial thickness and functionality during growth and composite processing.^51,59^ Core–shell architectures also allow the decoupling of functions (*e.g.*, a plasmonic or magnetic core with a chemically robust or polymer-affinitive shell), which is a key reason they are extensively studied across optics, catalysis, and surface-enhanced spectroscopy and are increasingly used as design motifs for composite interfaces.^51,59^ Plasmonic-oxide “core–shell-like” nanoarchitectures (*e.g.*, titania@gold) illustrate how combining dissimilar phases at the nanoscale can create synergistic optical/electronic behavior, a concept that is transferable to polymer nanocomposites seeking coupled multifunctionality.^60^

Multi-shell nanoparticles extend the core–shell concept by introducing multiple shells (solid-core/multi-shell or hollow-core/multi-shell). Layer-by-layer shell construction enables inorganic–inorganic, organic–organic, or inorganic–organic hybridization, producing hierarchical structures in which each shell can be tuned for specific tasks, such as diffusion resistance, optical/magnetic coupling, or improved chemical compatibility. Multi-shelled hollow micro-/nanostructures are particularly attractive because their geometry and composition can be manipulated simultaneously, offering a route to property improvement (*e.g.*, increased accessible surface area, controlled transport pathways, and improved stability) that can be exploited when such particles are embedded as multifunctional nanofillers in polymers.^61^

Janus nanoparticles possess two chemically/physically distinct “faces” (or compartments) within a single particle, yielding anisotropic interactions and directional assembly. Their functionality and performance depend strongly on the synthesis route and the resulting asymmetry, which can be used to tune interfacial anchoring, compatibilization, and stimulus responsiveness in polymers.^52^ More broadly, Janus particles have been recognized as a platform for encoding orthogonal surface chemistries and directional interactions, enabling controlled self-assembly and interfacial activity that can be harnessed in polymer matrices (*e.g.*, as stabilizers, catalysts, or compatibilizers).^62^

Decorated nanoparticles are nanoparticles whose surfaces are deliberately modified by grafted molecules, polymers, or nanoscale substructures, with the primary goal of tailoring wettability, surface energy, and specific interactions with polymer chains. Such decoration strategies are central to improving dispersion, reducing agglomeration, and strengthening interfacial adhesion—factors that directly control stress transfer and barrier performance in polymer nanocomposites.^20,56^ Chitosan-decorated nanoparticles are a well-established example of purposeful surface decoration that changes interfacial interactions and transport behavior; although widely explored in biomedical delivery contexts, the underlying principle, using a functional polymer shell/decoration to modulate particle–matrix interactions, maps directly onto polymer nanocomposite design logic.^63^

Heterodimer nanoparticles are formed by the controlled joining of two dissimilar nanoparticle domains (often *via* guest–host or seeded growth strategies), producing a single hybrid particle with an inter-domain junction. Because the two domains can retain their characteristic properties while coupling *via* an engineered interface, heterodimers are especially powerful for multifunctional composites (*e.g.*, simultaneously magnetic and plasmonic), where the junction morphology and interfacial chemistry strongly influence performance. Magnetic–plasmonic heterodimer platforms exemplify how domain coupling can integrate multiple functionalities into a single nanoscale filler motif and motivate analogous hybrid-filler strategies in polymer nanocomposites requiring coupled responses.^64^

In addition to single-particle hybrid architectures, hybridization is also widely implemented at the filler-system level (nanofiller design), where a polymer matrix is reinforced by either a single nanofiller type or a deliberately chosen combination of two or more nanofillers (hybrid nanofillers). Single-filler nanocomposites (*e.g.*, CNT-only systems) can be effective but often exhibit limitations in simultaneously optimizing conductivity, toughness, thermal transport, wear resistance, and barrier properties, because a single filler geometry cannot efficiently satisfy competing structure–property demands at practical loadings.^53,56^ By contrast, hybrid nanofiller systems exploit geometric and functional complementarity (*e.g.*, 1D fillers bridging 2D sheets, or hard nanoparticles decorating lubricious nanosheets), which can lower percolation thresholds, suppress restacking/agglomeration, and create hierarchical pathways for stress transfer or transport. For example, reduced graphene oxide/zinc sulfide (RGO/ZnS) hybrids have been reported to outperform single-component counterparts in tribological polymer coatings due to synergistic effects and improved dispersion enabled by anchoring nanoparticles on nanosheets,^55^ consistent with broader conclusions from recent reviews on hybrid nanofiller reinforcement and polymer nanocomposite tribology,^53,56^ Similarly, graphene-CNT hybrid reinforcement has been systematically highlighted as a route to multifunctional enhancement across polymer (and other) matrices by combining complementary dimensions and network formation mechanisms.^54^

### 2.4. Interfacial interaction mechanisms and structure-property relationships in hybrid polymer nanocomposites

**Fig. 4:**
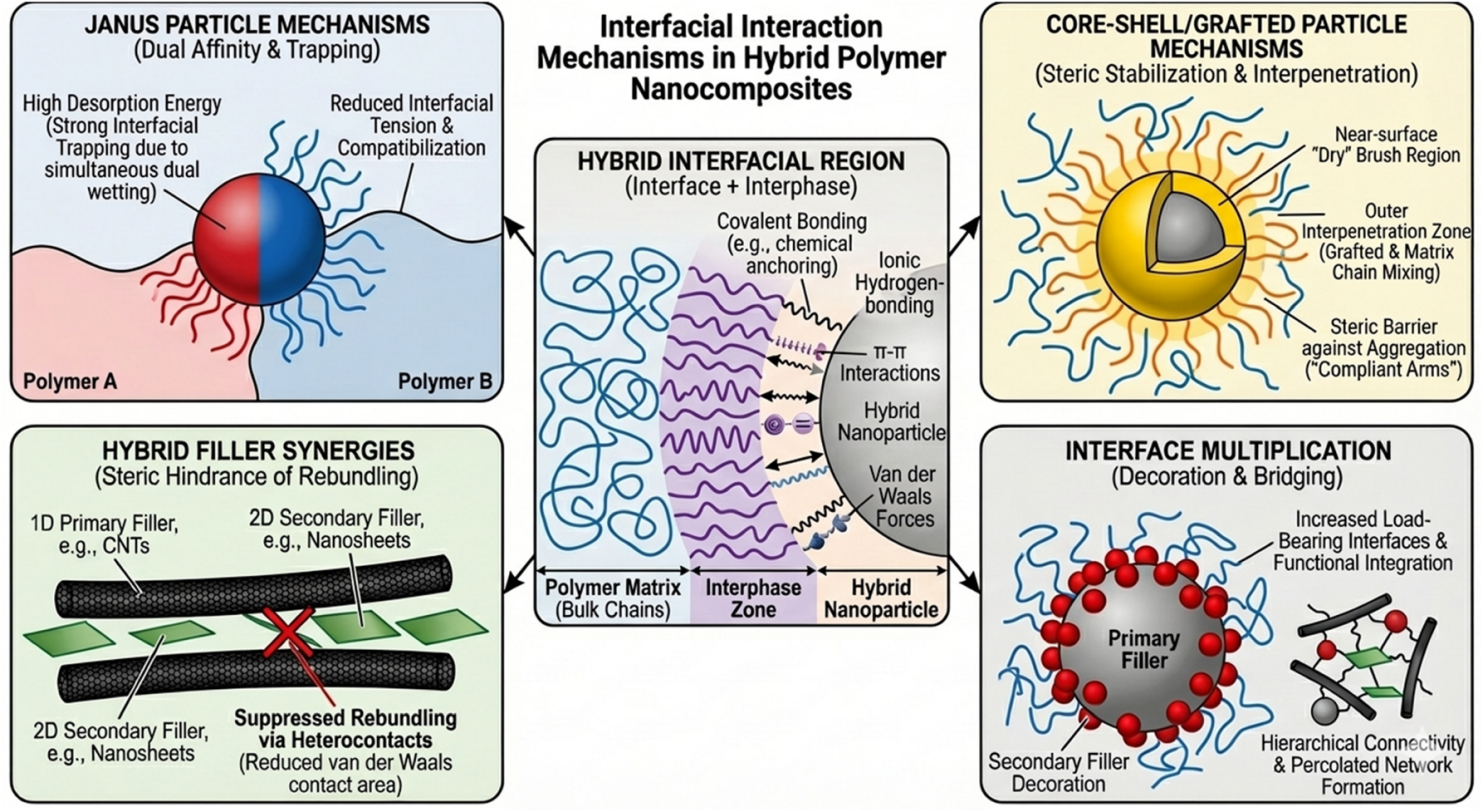
Interfacial interaction mechanisms in hybrid polymer nanocomposites.

The interfacial region between a polymer and a nanoscale additive, often described as an interface plus an interphase, links nanoparticle chemistry and architecture to dispersion, morphology, and structure–property relationships in hybrid polymer nanocomposites. In high-performance systems, the interphase can occupy a substantial volume fraction because of the large specific surface area of nanofillers; thus, macroscopic mechanical, thermal, barrier, and transport behavior often reflects interphase-controlled behavior rather than simple rule-of-mixtures averaging.^20,65^ The mechanisms of interfacial interactions in hybrid polymer composites are illustrated in Fig. 4.

**Fig. 5:**
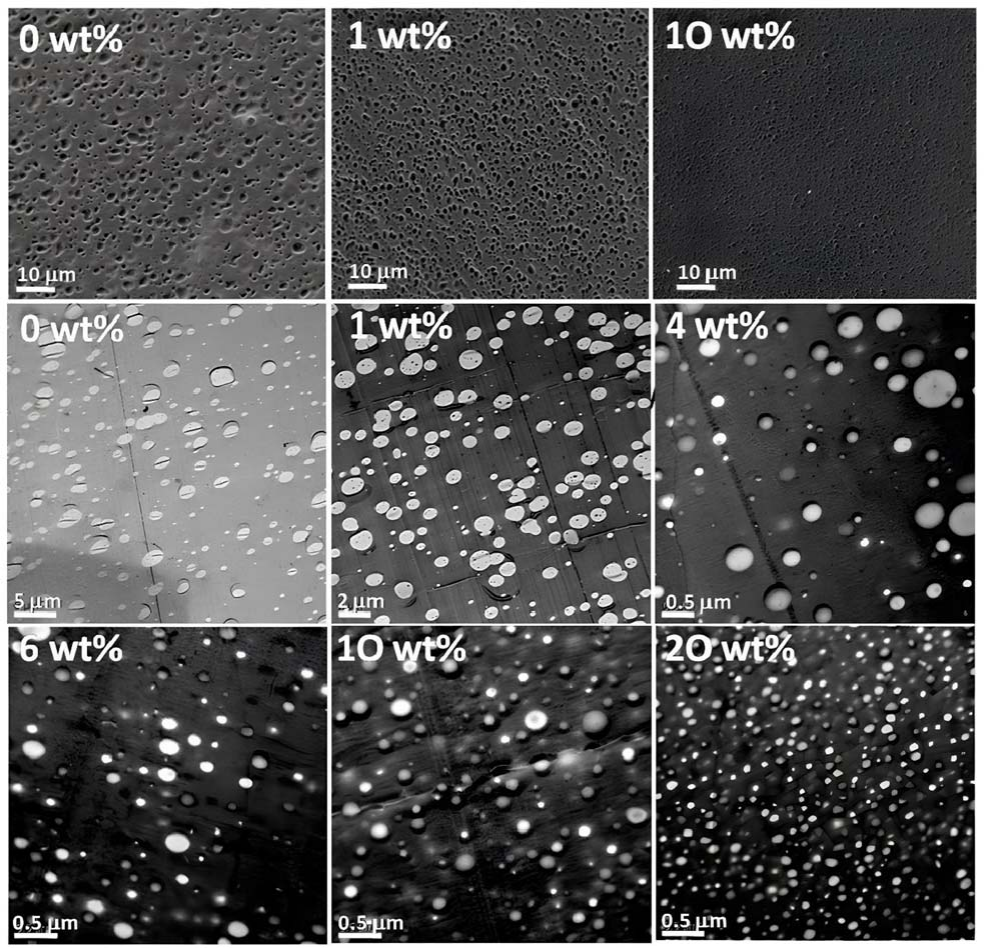
PS/PMMA blend morphologies showing progressive domain refinement and interfacial localization of Janus particles with increasing particle loading, reproduced from ref. 66 with permission from American Chemical Society, copyright 2008 (License No. 6296620155927).

A potent route to interfacial programming is to use anisotropic (Janus) particles. Janus particles possess two faces with distinct surface chemistries; consequently, one side can preferentially wet (or be enthalpically compatible with) one polymer environment while the other side prefers a second environment. This dual affinity can deterministically control where the particle resides (bulk *vs.* interface) and whether it remains dispersed or forms clusters. In a single-polymer matrix, Janus particles can remain well dispersed when the face compatible with the host polymer reduces the particle-polymer interfacial energy and promotes favorable mixing with surrounding polymer coils. Walther *et al.* demonstrated that polystyrene/polymethyl methacrylate (PS/PMMA) Janus particles remain dispersed in a PS matrix without forming large clusters, even for particles larger than the polymer coil size, because the PS hemisphere effectively “blends” with the PS matrix and stabilizes the particle.^66^ In immiscible polymer blends, Janus particles typically exhibit the strongest compatibilization effect: each face prefers a different polymer phase, driving migration to the polymer–polymer interface, reducing interfacial tension, suppressing coalescence, and refining the domain size distribution.^67^ This progressive domain refinement and interfacial localization with increasing Janus loading is consistent with the morphologies depicted in Fig. 5.^66^

**Fig. 6:**
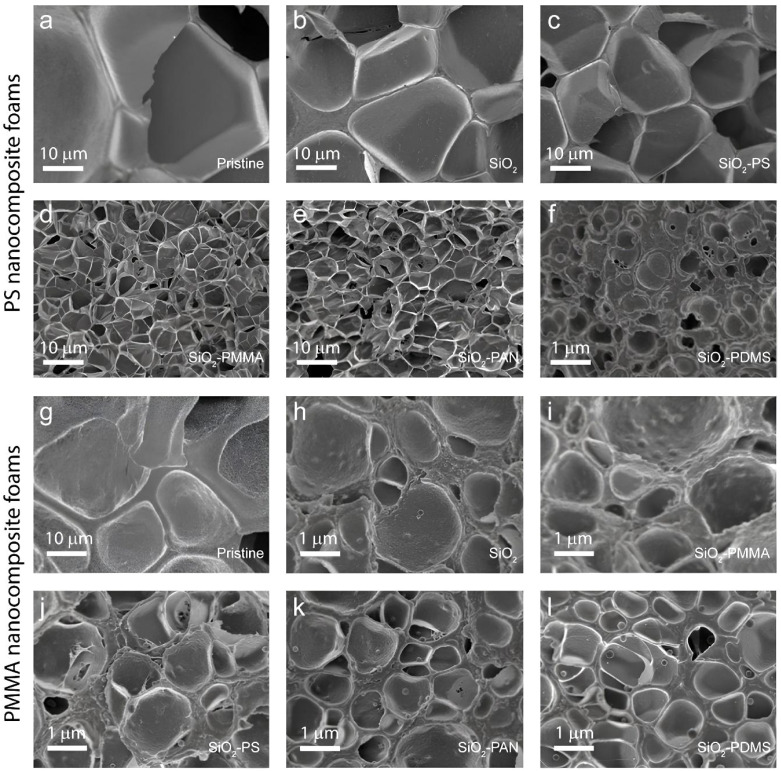
Scanning electron micrographs of the fractured cross-sections of PS- and PMMA-based nanocomposite foams containing different types of silica nanoparticles. Panels (a–f) show PS foams with (a) no nanoparticles, (b) bare SiO_2_, and silica particles grafted with (c) PS, (d) PMMA, (e) PAN, and (f) PDMS chains. Panels (g–l) correspond to PMMA-based foams with (g) no nanoparticles, (h) bare SiO_2_, and core–shell SiO_2_ particles grafted with (i) PMMA, (j) PS, (k) PAN, and (l) PDMS, highlighting the strong effect of polymer-grafted core–shell nanoparticles on cell size, cell density, and foam uniformity, reproduced from ref. 68 under CC BY-NC-ND 4.0 license.

A second widely used architecture for controlling interfacial physics is the core–shell (often polymer-grafted) hybrid nanoparticle (see Fig. 4), in which the shell serves as the “chemical contact layer” that governs wetting, dispersion, and stress/heat transfer. In polymer-grafted systems, a stable interphase forms around the particle because grafted chains alter local polymer density, segmental mobility, and entanglement statistics. Experimentally, Liu *et al.* showed that silica nanoparticles grafted with tailored polymer chains (PS, PMMA, PAN, PDMS) act as highly efficient nucleation sites in PS- and PMMA-based nanocomposite foams. Compared with neat polymers and bare silica systems, polymer-grafted core–shell silica produced substantially smaller cell sizes, narrower cell-size distributions, and higher cell densities (Fig. 6), indicating that molecularly engineered interfaces can stabilize gas–polymer interfaces during bubble growth while simultaneously improving filler-matrix compatibility.^68^

From a structure–property standpoint, mechanical reinforcement in hybrid nanocomposites is maximized when (i) fillers are well dispersed and (ii) interfacial bonding enables efficient load transfer and suppresses pull-out/sliding. Hybrid nanofillers can contribute through covalent, ionic, hydrogen-bonding, and π–π interactions, with each pathway modifying interphase stiffness and energy dissipation differently.^69^ Polymer-grafted nanoparticles offer a particularly tunable reinforcement strategy: grafted chains behave like compliant “arms” that improve particle wetting and reduce aggregation (aggregation creates stress concentrators and premature failure sites).^70^

Hybrid interfaces also enable synergistic reinforcement when a secondary nanocomponent increases the number and quality of load-bearing interfaces. For example, Zhang *et al.* prepared SiO_2_@h-BN/PVA composites and showed that improved filler distribution (vacuum filtration *vs.* solution casting) and interface multiplication *via* SiO_2_ decoration significantly enhanced both stiffness and strength: neat PVA (Young's modulus of 1.8 GPa and tensile strength of 47 MPa) increased to 4.2 GPa and 110 MPa with 14.5 wt% h-BN, and further to 5.9 GPa and 156 MPa with 15.2 wt% SiO_2_@h-BN (14.5 wt% h-BN and 0.7 wt% SiO_2_). The additional SiO_2_ introduces more filler-matrix interfaces, which can deflect and arrest cracks, thereby improving fracture resistance.^71^ More broadly, interphase engineering (*via* grafting, surface functionalization, or Janus design) reduces interfacial defects that would otherwise enable debonding, filler pull-out, or interfacial sliding; in high-impact formulations, the filler becomes functionally integrated into the polymer network, improving modulus, strength, toughness, and thermal stability simultaneously.^20,71^

Interfacial chemistry also governs modified dispersion, which directly controls both property reproducibility and percolation-driven multifunctionality. In hybrid nanocomposites, dispersion is further influenced by heterocontacts between filler types (*e.g.*, 1D–2D or 1D–0D contacts), where the secondary component can sterically hinder rebundling or reduce the effective van der Waals contact area.

These interfacial and dispersion changes significantly influence thermal behavior, including glass transition temperature (*T*_g_) and thermal transport. *T*_g_ shifts with nanoparticle addition can decrease, increase, or remain near-constant, reflecting competition between (a) mobility suppression/confinement near attractive or grafted interfaces (which tends to increase *T*_g_) and (b) interfacial layers that increase configurational entropy or act as “lubricating” organic shells (which can decrease *T*_g_). Hybrid fillers also affect thermal conductivity through interfacial thermal resistance (Kapitza resistance): functional groups on boron nitride nanosheets can enhance vibrational coupling with polymer chains, reducing interfacial thermal resistance and enabling more effective phonon transport across the filler-polymer boundary, which is critical for thermal management in packaging and electronics.^72^

**Fig. 7:**
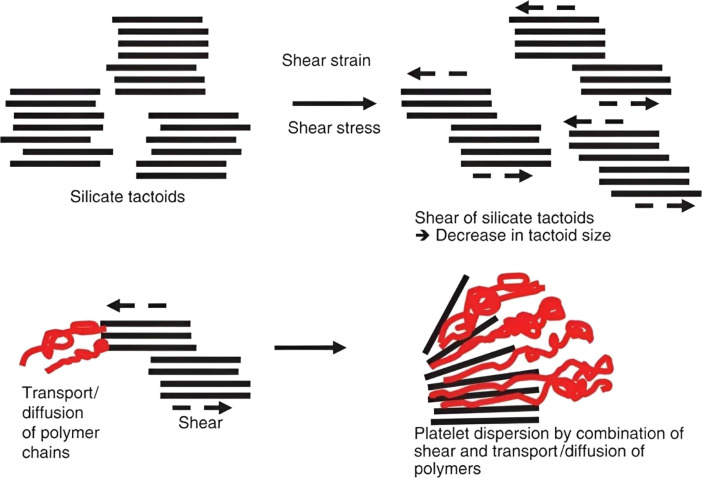
Shear-induced delamination of layered nanofillers, showing how large tactoids break into progressively smaller stacks during melt mixing, reproduced from ref. 73 with permission from Springer Nature, copyright 2011 (License No. 6296951244595).

Processing route-dependent dispersion (solution casting, melt blending, reactive/*in situ* polymerization) further couples to interfacial physics by determining when and how the interphase forms and whether filler networks become kinetically trapped. In solvent-cast systems, the lower viscosity of the medium facilitates particle mobility, but strong attractions (*e.g.*, CNT bundling) still impose large energy barriers to full debundling. The progressive shear-induced delamination of layered fillers (Fig. 7) illustrates a general pathway by which tactoids break down during melt mixing, and similar mechanisms can assist dispersion of hybrid systems containing 2D components.^73^

Reactive and *in situ* polymerization routes offer a distinct advantage because nanoparticles are dispersed in low-viscosity monomers before viscosity rises during polymerization. Chemical anchoring (covalent or strong specific interactions) can lock in early-stage dispersion states, while controlled radical polymerization methods (*e.g.*, atom transfer radical polymerization) enable well-defined polymer growth directly from nanoparticle surfaces (“grafting-from”), generating thick brushes that provide steric barriers against aggregation and can even template ordered particle arrangements with spacing controlled by chain length.^74^

**Fig. 8:**
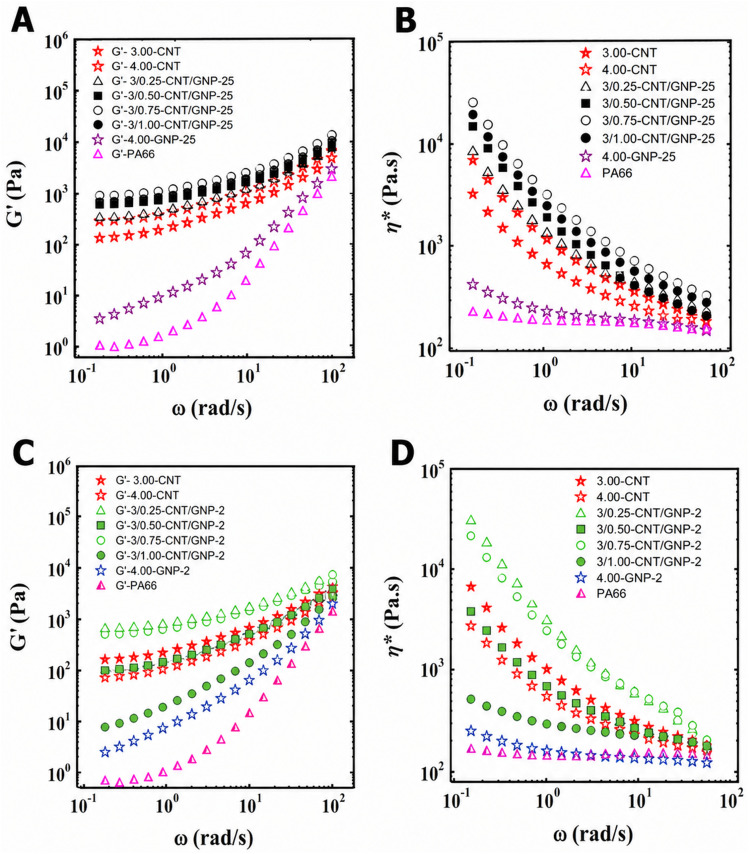
Storage modulus (*G*′) and complex viscosity (η*) as a function of angular frequency for PA66, binary CNT and GNP nanocomposites, and CNT/GNP hybrid filler nanocomposites containing 3 wt% CNT with varying GNP-25 content (a and b) and GNP-2 content (c and d). The progressive development of a low-frequency *G*′ plateau with increasing hybrid filler loading is characteristic of a percolated filler network. The earlier onset and stronger plateau in the CNT/GNP-25 hybrid system reflect enhanced network connectivity enabled by the 1D–2D hybrid architecture, while the GNP-2 system exhibits viscosity reduction alongside partial network disruption, illustrating the competing effects of filler geometry on rheological percolation, adapted from ref. 75 with permission from John Wiley and Sons, copyright 2021 (License No. 6296960303486).

Finally, rheology and percolation provide sensitive, quantitative readouts of dispersion quality and network formation in hybrid systems. When nanoparticles form a connected, percolated structure, the melt exhibits a solid-like signature: the storage modulus (*G′*) increases and may develop a low-frequency plateau (Fig. 8), indicating that the filler network can support stress under slow deformation. In CNT/GNP hybrid systems, this response depends strongly on filler geometry, size, and dispersion. Larger GNP-25 platelets can preserve or strengthen the CNT-rich network, whereas smaller GNP-2 platelets may reduce *G′* and complex viscosity (η*) by interrupting CNT contacts and improving melt mobility.^75^ This behavior shows that hybridization does not always strengthen the rheological network; rather, the final response reflects the balance between CNT bridging, platelet contacts, interfacial interactions, and processing-induced dispersion. Shear-induced delamination and rearrangement during melt mixing can assist dispersion of layered fillers and promote network development when aggregation is controlled.^73^ Electrical percolation is also strongly shape-dependent; CNTs exhibit low percolation thresholds because their high aspect ratio favors long-range conductive pathways.^76^ Hybridization can further improve connectivity when 1D and 2D fillers create effective bridging and contact zones, although poorly dispersed or mismatched fillers may disrupt rather than reinforce the network. Overall, low-frequency *G′* plateaus, controlled shear-thinning, suitable η* values, and low percolation thresholds together indicate a well-connected hybrid network that can support processability, mechanical reinforcement, thermal transport, and electrical functionality.

## 3. Manufacturing techniques of polymer nanocomposites with hybrid nanoparticles

The fabrication of polymer nanocomposites incorporating hybrid nanoparticles demands sophisticated processing strategies that ensure controlled particle dispersion, interfacial compatibility, and preservation of multifunctional properties. Manufacturing approaches are broadly categorized into bottom-up synthesis, top-down processing, and advanced emerging techniques, each presenting distinct advantages and inherent limitations that influence scalability, reproducibility, and final composite performance.

### 3.1. Bottom-up approaches

**Fig. 9:**
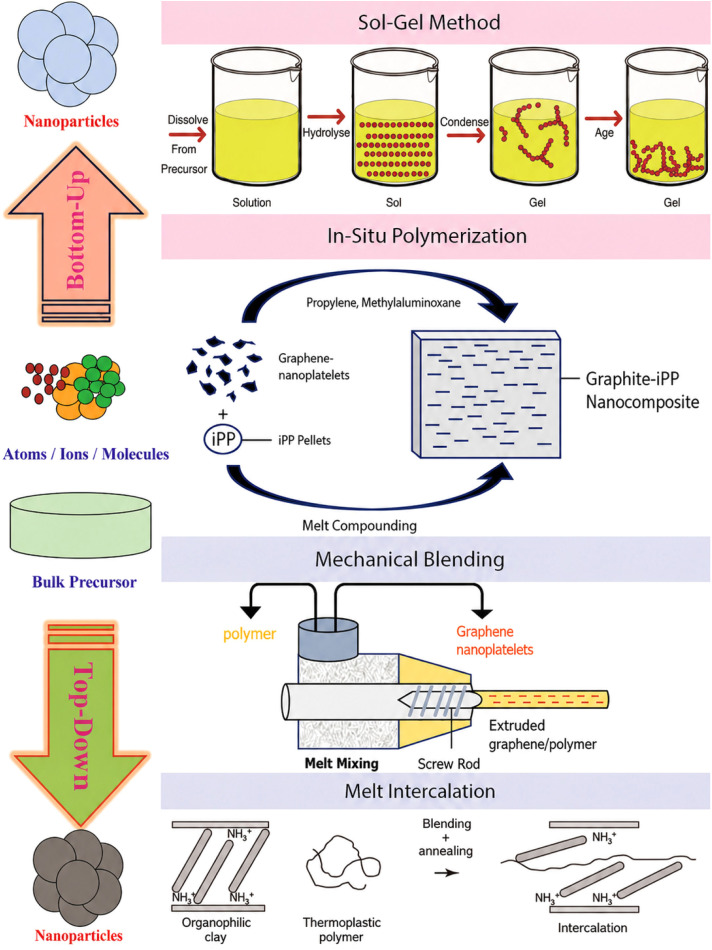
Various top-down (mechanical blending, melt intercalation) and bottom-up approaches (sol–gel method, *in situ* polymerization) for polymer nanocomposite fabrication; top-down and bottom-up schematic reproduced from ref. 77 under CC BY 4.0 license; sol–gel schematic reproduced from ref. 78 with permission from Elsevier, copyright 2022 (License No. 6297020254506); *in situ* polymerization and mechanical blending schematics reproduced from ref. 79 with permission from Elsevier, copyright 2020 (License No. 6300621237469); melt intercalation schematic reproduced from ref. 80 under CC BY 4.0 license.

Bottom-up methodologies construct nanocomposites through molecular-level assembly, offering precise control over particle size, morphology, and interfacial chemistry. These techniques facilitate the integration of chemically distinct nanoparticles into polymer matrices *via* controlled nucleation and growth. Fig. 9 shows the various top-down (sol–gel method, *in situ* polymerization) and bottom-up approaches (mechanical blending, melt intercalation).

#### 3.1.1. Sol–gel method

The sol–gel process enables the *in situ* formation of inorganic nanoparticles within polymer matrices through hydrolysis and condensation reactions of metal alkoxide precursors. This approach allows simultaneous synthesis and dispersion of hybrid nanoparticles, resulting in intimate polymer–particle interfaces that enhance load transfer efficiency and interfacial adhesion.^81^ Owing to its high versatility, the sol–gel method facilitates the fabrication of complex hybrid architectures, including core–shell structures and hierarchical nanocomposites, through the sequential deposition of different metal oxide phases.^82^

Despite these advantages, the sol–gel method presents several significant challenges that limit its industrial applicability. The process typically involves extended gelation and drying stages that often span several days, increasing energy consumption and reducing throughput.^83^ Conventional convective and conductive heating methods often result in inefficient reaction kinetics and heterogeneous temperature distributions, compromising nanostructural uniformity and batch-to-batch reproducibility.^84,85^ During drying, capillary stresses can induce gel shrinkage, cracking, and network collapse, leading to microstructural defects that degrade the mechanical integrity of the final nanocomposites.^86^ In addition, the sol–gel process is susceptible to reaction parameters such as pH, temperature, and precursor concentration, necessitating strict control to ensure reproducibility.^87,88^ Incomplete hydrolysis and condensation reactions may leave residual alkoxy groups, adversely affecting the purity, optical transparency, dielectric behavior, and functional performance of the resulting nanoparticles.^89^ Furthermore, reliance on inorganic or organic solvents raises environmental and safety concerns, requiring specialized handling and waste-management infrastructure.^90^ Recent developments have sought to address these limitations through microwave-assisted sol–gel processing, which significantly accelerates reaction kinetics, improves thermal homogeneity, and reduces processing time.^84^ However, large-scale implementation remains challenging due to limited microwave penetration depth and non-uniform electromagnetic fields, which complicate uniform heating in industrial-scale reactors.

#### 3.1.2. *In situ* polymerization

*In situ* polymerization is a technique in which nanomaterials, either pre-formed or synthesized *in situ*, are dispersed directly in a liquid monomer in the presence of a catalyst or polymerization initiator to form polymer nanocomposites. Because polymerization proceeds entirely within the continuous phase (rather than at an interface, as in interfacial polymerization), the method enables high nanomaterial loadings while maintaining nanoscale dispersion and good miscibility across diverse polymer matrices.^47^ It is compatible with both radical and condensation polymerization, making it applicable to thermoplastics, thermosets, and elastomers. Common systems include melamine-formaldehyde and urea-formaldehyde resins; in a typical encapsulation route, high-shear mixing emulsifies an oil phase in water, and melamine resin is added, followed by controlled acidification, which induces polycondensation to form microcapsules with crosslinked resin shells.^91^ Beyond encapsulation, sequential addition of different nanoparticle types during polymerization can generate graded or hierarchical hybrid architectures, enhancing multifunctional performance.^92^

Despite these advantages, achieving stable and uniform nanoparticle dispersion throughout polymerization remains a major challenge. Inorganic nanoparticles are typically hydrophilic, whereas most polymer matrices are hydrophobic, leading to aggregation and phase separation unless surface functionalization with compatibilizing agents is employed.^93^ The effectiveness of this functionalization depends critically on grafting density and ligand chemistry: insufficient modification results in weak interfacial adhesion and poor dispersion, while excessive functionalization can restrict polymer chain mobility and reduce the *T*_g_.^94^ As polymerization proceeds, the increasing viscosity immobilizes nanoparticle agglomerates, making post-gelation dispersion correction impractical.^95^ In addition, nanoparticle surfaces may catalyze premature polymerization or induce side reactions, thereby altering molecular weight distribution and crosslink density.^93^

From an industrial perspective, large-scale implementation is further complicated by processing and equipment limitations. The high viscosity of filled systems hinders effective mixing, while the abrasiveness of inorganic nanoparticles accelerates wear in pumps, mixers, and extruders.^96^ Process control becomes increasingly complex in hybrid systems containing multiple nanoparticle types, as each component may require distinct dispersion strategies and may exert competing effects on polymerization kinetics.^93,95,96^

### 3.2. Top-down approaches

In the context of polymer nanocomposites, top-down techniques focus on mechanically dispersing pre-synthesized hybrid nanoparticles into polymer matrices. These methods are particularly attractive due to their scalability and compatibility with established thermoplastic processing routes, including melt mixing, extrusion, and injection molding. As a result, top-down approaches are widely adopted in industrial applications where large-scale production, process robustness, and cost efficiency are critical.

#### 3.2.1. Mechanical blending and melt mixing

Mechanical blending is the most widely used top-down approach for producing polymer-based composites, including metal/binder, ceramic/binder, and polymer/filler systems, particularly for large-scale applications such as 3D printing feedstocks. The process involves mechanically mixing polymer powders or melts with fillers, metal/binder, or ceramic/binder powders using high-shear equipment such as kneaders, internal mixers, or extruders. When blending is performed in the molten state, the process is referred to as melt mixing or melt blending, where polymer pellets are melted, combined with additives, subjected to shear forces, and subsequently cooled to form solid composites.^97^ Melt blending is especially attractive for thermoplastic systems due to its cost-effectiveness, solvent-free operation, and compatibility with existing industrial processing infrastructure. High-shear mixing during extrusion, particularly in twin-screw extruders, facilitates filler dispersion by breaking up particle agglomerates and enabling continuous production with consistent quality.^98^ In addition, melt processing prevents filler re-aggregation during solvent evaporation and eliminates the need for chemical solvents or extensive surface modification, making it environmentally friendly and economically viable.^79^

Despite these advantages, mechanical blending poses significant challenges for achieving uniform nanoparticle dispersion, especially in hybrid nanoparticle systems. Differences in particle size, density, and surface chemistry lead to non-uniform flow behavior and segregation during feeding and processing, resulting in heterogeneous filler distributions.^94^ High-energy ball milling is often employed to improve dispersion; however, this approach offers limited control over particle size and morphology and typically produces broader particle size distributions than chemical synthesis methods.^89,99^ Moreover, contamination from milling media and chamber walls can compromise nanoparticle purity and composite performance.^100^ Ball milling also requires long processing times and high energy input to achieve nanoscale dispersion, generating excessive frictional heat that can degrade thermally sensitive polymers or induce phase transformations in inorganic nanoparticles.^101^ During melt processing, elevated temperatures (typically 180–300 °C) may decompose organic surface modifiers on hybrid nanoparticles, exposing bare inorganic surfaces that readily re-agglomerate due to strong van der Waals interactions.^92^ Additionally, the high viscosity of heavily filled polymer melts limits achievable filler loadings and often necessitates higher processing temperatures, further increasing the risk of thermal degradation. Recent studies indicate that the use of compatibilizers, reactive processing aids, or tailored screw designs can improve dispersion and interfacial adhesion in mechanically blended nanocomposites. However, these strategies increase formulation complexity, processing costs, and sensitivity to processing conditions, which may offset their benefits in large-scale manufacturing.^89^

#### 3.2.2. Melt intercalation

Melt intercalation is the standard and most industrially relevant method for producing thermoplastic polymer nanocomposites, particularly those based on layered fillers such as clays and clay-supported hybrid nanoparticles. The process involves annealing the polymer matrix at elevated temperatures, typically between 190 °C and 220 °C, followed by the incorporation of fillers under shear to promote polymer chain diffusion into the interlayer galleries.^78^ Successful intercalation is driven by entropy gain and favorable polymer–surface interactions, enabling the formation of intercalated or fully exfoliated nanocomposite morphologies.^37,102^ When the electrostatic forces between filler layers are sufficiently weakened, often through organic surface modification, the polymer chains can penetrate the galleries, significantly increasing the interfacial area and enhancing mechanical reinforcement, barrier performance, and thermal stability.^103^ As a result, melt intercalation has demonstrated significant industrial potential, particularly for polymer/clay nanocomposites.^38^

The effectiveness of melt intercalation depends strongly on the thermodynamic compatibility between the polymer and the layered filler. Non-polar polymers such as polyethylene exhibit poor affinity for hydrophilic silicate layers due to their chemically inert backbones, necessitating the use of compatibilizers or surface-modified fillers.^104^ In polypropylene systems, grafting with maleic anhydride or hydroxyl-functional groups has been shown to facilitate intercalation with montmorillonite by improving interfacial interactions.^105^ However, even in modified systems, weak polymer–surface interactions can lead to thermodynamically unstable interlayer structures that collapse under processing shear, limiting exfoliation and resulting in tactoid reformation.^106^

Achieving complete exfoliation is even more challenging for hybrid layered nanoparticles, where secondary nanoparticles embedded within the interlayers introduce steric hindrance that restricts polymer diffusion. This frequently leads to incomplete intercalation and mixed tactoid-exfoliated morphologies.^107^ In addition, the processing temperature window is narrow: insufficient temperatures hinder polymer mobility, while excessive heat degrades organic modifiers, releasing volatile byproducts that generate voids and compromise composite performance.^107,108^ Furthermore, the success and reproducibility of melt intercalation depend critically on filler characteristics such as aspect ratio, surface charge density, and modifier chemistry, which can vary substantially between batches, particularly for hybrid nanoparticles.^107^ These factors introduce processing variability and complicate scale-up, despite the method's compatibility with conventional melt-processing infrastructure.

### 3.3. Advanced techniques

#### 3.3.1. Layer-by-layer assembly

Layer-by-layer (LbL) assembly is a versatile bottom-up technique for fabricating polymer nanocomposites by sequentially depositing polymers and nanoparticles, enabling precise control over interfacial structure, composition, thickness, and architecture at the nanoscale.^109^ The method allows independent engineering of individual layers, making it particularly effective for constructing stratified or gradient hybrid nanocomposites with tailored multifunctional properties.

**Fig. 10:**
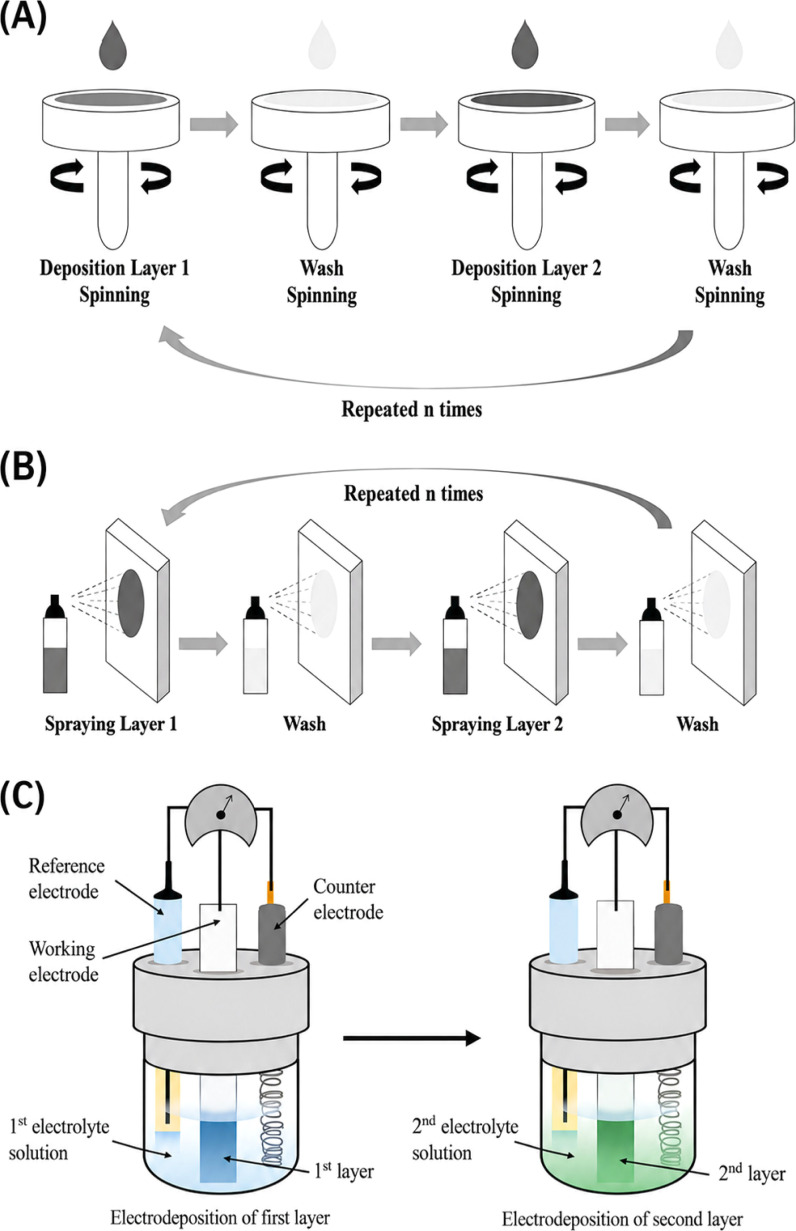
Three LbL deposition technologies: (A) spin-coating assembly and (B) spray-coating assembly, reproduced from ref. 113 with permission from Elsevier, copyright 2020 (License No. 6304800189179); (C) electrodeposition assembly, reproduced from ref. 115 under CC BY 4.0 license.

LbL assembly can be implemented using several deposition strategies, including immersive or dip coating, spin coating (Fig. 10A), spray coating (Fig. 10B), electromagnetic assembly (Fig. 10C), and fluidic or microfluidic assembly, each offering distinct advantages in deposition rate, film uniformity, and substrate compatibility.^110^ Spin assembly exploits centrifugal, viscous, and electrostatic forces to accelerate deposition, making it suitable for large-area substrates.^110,111^ This control over deposition forces enables faster and more uniform coating, particularly on larger substrates.^112,113^ Spray assembly enables rapid multilayer formation through controlled atomization and deposition,^113,114^ while electromagnetic assembly uses electric or magnetic fields to enhance interfacial interactions, particularly for charged or magnetically responsive components.^115^

For hybrid nanocomposites, LbL assembly enables precise spatial organization of multiple nanoparticle types within periodic or graded multilayer architectures, facilitating property optimization in applications such as electromagnetic shielding, barrier coatings, and controlled release systems.^111^ However, despite its structural precision, LbL assembly faces significant scalability and throughput limitations. Immersive and fluidic methods are inherently time-intensive due to repeated deposition and rinsing cycles, while spin and spray techniques, although faster, often suffer from non-uniform thickness, radial gradients, or heterogeneous coverage caused by centrifugal forces or aerosol droplet size distributions.^112,114^ Spray processes further raise environmental and health concerns due to solvent volatility, requiring specialized ventilation systems.^116^ Extending LbL assembly beyond thin films remains challenging. Polymer melt-based LbL and multilayer co-extrusion processes suffer from viscosity mismatches and interfacial instabilities, leading to non-uniform layer thickness and restricting applications primarily to films or sheets.^117^ Similarly, crystallization-and-annealing strategies are sensitive to cooling rates and crystallization kinetics, leading to limited reproducibility and structural stability.^118^ Consequently, despite its exceptional control over nanoscale architecture, LbL assembly remains confined mainly to thin-film and surface-engineered nanocomposites rather than bulk structural materials.

#### 3.3.2. Additive manufacturing (3D printing)

Additive manufacturing (AM), commonly referred to as 3D printing, fabricates objects layer by layer and enables the production of geometrically complex polymer nanocomposites that are difficult or impossible to realize using conventional processing routes.^119^ Polymers remain the most widely used materials, although metals and ceramics are increasingly explored. AM is particularly attractive for incorporating continuous fibers, short fibers, and nanofillers, including carbon nanotubes (CNTs), graphene nanosheets (GNSs), and hybrid nanoparticles, to enhance mechanical, thermal, electrical, and biocompatibility properties.^120^ Consequently, AM has emerged as a promising platform for fabricating multifunctional polymer-graphene and hybrid nanocomposites.

**Fig. 11:**
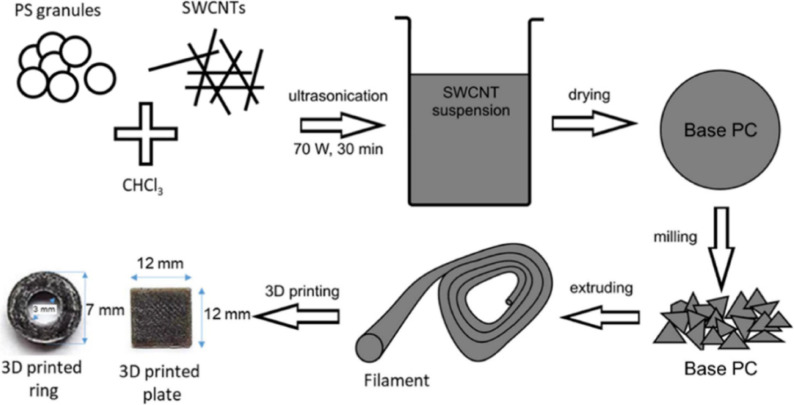
A diagram of a 3D printing approach for carbon nanotube-polymer composite fabrication, reproduced from ref. 121 under CC BY 4.0 license.

In a typical workflow, hybrid nanoparticles are first dispersed into a polymer matrix *via* solution mixing or melt compounding, often assisted by ultrasonication or high-shear mixing, as illustrated in Fig. 11. The resulting composite is processed into printable feedstocks, such as filaments, resins, or powders, by extrusion or curing. These feedstocks are then selectively deposited using AM techniques, including material extrusion, vat photopolymerization, powder bed fusion, or binder jetting, to fabricate complex components, which are subsequently post-processed and characterized. Recent demonstrations include CNT-graphene hybrid filaments with synergistic electromagnetic interference (EMI) shielding and mechanically reinforced photopolymer resins containing oxide-based hybrid nanoparticles for biomedical applications.^121^

Despite its versatility, AM of hybrid nanocomposites faces significant technical challenges. In material extrusion, precise rheological control is required: excessive viscosity leads to nozzle clogging and print defects, whereas low viscosity compromises shape fidelity and interlayer adhesion.^121^ Density and surface chemistry mismatches among hybrid nanoparticles promote sedimentation during prolonged printing, generating spatial heterogeneity and mechanical anisotropy. The intrinsic layer-by-layer nature of AM further introduces direction-dependent properties, as interlayer bonding is typically weaker than intralayer cohesion.^119^

Process-specific limitations also persist. Vat photopolymerization suffers from nanoparticle-induced light scattering and absorption, reducing curing depth and necessitating post-curing steps that may cause dimensional distortion.^122,123^ Powder bed fusion often yields porous structures due to incomplete sintering around nanoparticles and exposes fillers to high temperatures that risk degradation or unwanted phase transformations.^124^ Binder jetting is less suitable for highly porous nanocomposites, while sheet lamination remains constrained in material selection and achievable geometries.^123,124^

A fundamental bottleneck across all AM techniques is achieving homogeneous dispersion of multiple nanoparticle types within printable feedstocks. Current dispersion strategies, such as intensive ultrasonication or high-shear mixing, can damage nanoparticle morphology or disrupt surface functionalization, undermining the intended synergistic effects of hybrid architectures. Addressing these challenges is essential for translating the design freedom of AM into reliable, high-performance hybrid nanocomposite components.

### 3.4. Emerging synthesis techniques

#### 3.4.1. Microfluidic synthesis

Microfluidic synthesis enables the continuous, highly controlled fabrication of hybrid nanoparticles by exploiting laminar flow, confined geometries, and rapid mixing in microreactors.^125^ Through flow-focusing, droplet microreactors, or confined co-flow mixing, microfluidic platforms provide precise control over nucleation, growth, and polymer-nanoparticle assembly, resulting in hybrid nanoparticles with narrow size distributions, well-defined architectures, and superior batch-to-batch reproducibility compared to bulk synthesis methods.^126^ Additional advantages include on-chip functionalization, real-time process monitoring, and compatibility with shear-sensitive polymers. A representative example is the microfluidic fabrication of lipid–polymer hybrid nanoparticles composed of a poly(lactic-*co*-glycolic acid) core and a lipid shell. Compared to single-component polymeric nanoparticles or conventional liposomes, these hybrids exhibit enhanced drug loading capacity, improved colloidal stability in physiological environments, tunable surface functionality, prolonged systemic circulation, and the ability to co-deliver hydrophilic and hydrophobic therapeutics.^126^ Microfluidic control further enables tuning of core–shell morphology and particle size. However, achieving precise control over lipid shell structure (monolayer *versus* bilayer) and fully understanding polymer-lipid interfacial interactions remains an open challenge.^126^ Beyond biomedical systems, microfluidic platforms allow sequential reagent introduction and layer-by-layer assembly of complex hybrid architectures, such as metal cores with polymer shells and secondary nanoparticle coatings, within a single integrated device. This eliminates intermediate purification steps, reducing contamination risk and processing time.^125^

Despite these advantages, microfluidic synthesis faces significant scalability constraints. The small channel dimensions (typically 10–500 µm) limit volumetric throughput to milliliters per hour, necessitating extensive device parallelization for industrial production.^125^ Channel fouling caused by nanoparticle adhesion alters flow profiles and mixing efficiency over time, requiring frequent maintenance or device replacement. High-aspect-ratio channels also generate substantial pressure drops, requiring robust pumping systems and leak-free connections. Moreover, the technique is poorly suited for high-viscosity polymer solutions, which suppress diffusive mixing and compromise assembly control.^126^ Finally, the need for specialized microfabrication infrastructure and operational expertise imposes high capital and training costs, limiting widespread adoption despite the method's exceptional precision.

#### 3.4.2. Atomic layer deposition

Atomic layer deposition (ALD) is a vapor-phase technique based on self-limiting surface reactions that enables the conformal deposition of ultrathin inorganic coatings such as Al_2_O_3_, TiO_2_, and ZnO onto polymer substrates or dispersed nanoparticles with angstrom-level thickness control.^127^ This exceptional precision allows the fabrication of well-defined core–shell hybrid nanoparticles and polymer-inorganic architectures with tunable interfacial chemistry, enhanced thermal stability, and improved barrier performance. By uniformly coating particle surfaces, ALD suppresses nanoparticle aggregation through electrostatic or steric stabilization, facilitating homogeneous dispersion even at relatively high filler loadings.^128^ The layer-by-layer growth mechanism of ALD further enables the construction of multilayer hybrid coatings (*e.g.*, Al_2_O_3_/TiO_2_ stacks) with atomically sharp interfaces, allowing fine control over optical, electrical, and gas-barrier properties through compositional and thickness engineering. ALD processes can be conducted at moderate temperatures (typically 80–300 °C), which are compatible with many thermoplastics, although they are still above the *T*_g_ of some commodity polymers.

Hybrid ALD-modified nanocomposites demonstrate synergistic multifunctionality compared to single-filler systems. For example, ZnO-coated TiO_2_-reinforced PLA nanocomposites combine TiO_2_-driven bulk mechanical reinforcement with ZnO-derived surface hydrophobicity and antimicrobial activity, making them promising candidates for active packaging applications.^128^ Such hybrid designs enable simultaneous optimization of surface and bulk properties while preserving barrier performance. However, in antimicrobial systems, achieving strong biological activity may require UV activation, adding a functional constraint.

Despite these advantages, ALD faces significant barriers to widespread adoption in hybrid nanocomposite manufacturing. Deposition rates are inherently slow (≈0.1–2 Å per cycle), requiring hundreds of cycles and processing times spanning hours to days, which severely limits throughput and economic viability for bulk production.^127^ Capital costs are high due to the need for vacuum systems, precise precursor delivery, and heated reaction chambers. Polymer substrates introduce additional challenges, including subsurface precursor diffusion, which can cause non-uniform coatings, and polymer plasticization, as well as strict temperature constraints imposed by polymer thermal sensitivity.^128^ Moreover, achieving uniform ALD coatings on nanoparticles typically requires specialized fluidized-bed or rotary reactors, further increasing process complexity. As a result, while ALD offers unparalleled control over hybrid nanostructure interfaces and functionality, its application in polymer-based hybrid nanocomposites remains largely exploratory, with most demonstrations confined to thin coatings or low-volume, high-value systems rather than scalable bulk materials.^128^

#### 3.4.3. Click chemistry functionalization

Click chemistry, most notably copper-catalyzed azide–alkyne cycloaddition (CuAAC) and thiol–ene/thiol–yne reactions, provides a powerful and versatile strategy for covalently functionalizing polymer chains and nanoparticle surfaces in hybrid nanocomposites.^129,130^ These reactions proceed under mild conditions, exhibit high selectivity and near-quantitative yields, and tolerate a broad range of functional groups, enabling precise control over grafting density, polymer architecture, and interfacial chemistry. As a result, click-enabled grafting significantly improves nanoparticle dispersion, polymer compatibility, and interfacial load transfer, which are critical for mechanical, electrical, and functional performance. The orthogonality of click reactions further enables sequential or simultaneous grafting of different polymers onto hybrid nanoparticles bearing complementary functional groups, facilitating the formation of Janus, patchy, or hierarchically functionalized surfaces that direct controlled assembly and multifunctionality.^131^ This level of architectural control is challenging to achieve using conventional physical adsorption or non-selective coupling strategies.

A representative example is the work of Shao *et al.*, who developed a molecularly imprinted polymer (MIP) grafted onto graphene oxide-magnetite (GO@Fe_3_O_4_) hybrid nanoparticles *via* CuAAC coupling of azide-functionalized GO@Fe_3_O_4_ with alkyne-terminated RAFT (reversible addition–fragmentation chain transfer) agents, followed by surface-initiated RAFT polymerization. This approach integrates magnetic separability (Fe_3_O_4_), high surface area and conductivity (GO), and selective molecular recognition (MIP) into a single sensing platform for ultrasensitive detection of tetrabromobisphenol A (TBBPA). Compared with single-filler systems, the click-functionalized hybrid exhibits superior performance due to stable covalent grafting, synergistic multifunctionality, and well-defined imprinted polymer architectures.^132^

Despite these advantages, click chemistry functionalization faces several practical limitations. Residual copper from CuAAC reactions can induce cytotoxicity in biomedical applications and may catalyze polymer degradation, necessitating extensive purification steps that increase processing complexity and cost.^130^ Diffusion limitations within dense nanoparticle aggregates can slow functionalization kinetics, particularly for larger or highly packed particles, often requiring prolonged reaction times or elevated catalyst concentrations. Moreover, the multistep synthesis route, surface activation, click coupling, and controlled polymerization reduce overall yield and scalability. The relatively high cost of functional monomers, azide/alkyne reagents, and controlled radical polymerization agents (*e.g.*, RAFT initiators) further restricts the economic viability of click-based strategies for large-volume or commodity nanocomposite applications.^132^

### 3.5. Comparative perspective

Table 4 compares the principal synthesis routes for polymer nanocomposites incorporating hybrid nanoparticles, outlining their core processing principles, key advantages, and major limitations. However, selecting appropriate manufacturing techniques for hybrid nanoparticle–polymer composites requires careful consideration of property requirements, production scale, and economic constraints. Bottom-up approaches (sol–gel, *in situ* polymerization) offer superior control over nanoscale architecture and interfacial chemistry, making them attractive for high-performance applications (aerospace, biomedical) where property optimization justifies the elevated processing costs. Conversely, top-down methods (melt mixing, extrusion) offer industrial scalability and compatibility with existing infrastructure, making them suitable for commodity applications despite inherent dispersion limitations. Emerging techniques represent promising research directions but require substantial development before industrial implementation. Microfluidic synthesis should overcome throughput limitations through numbering-up strategies or by integrating continuous processing. ALD needs substantial rate acceleration and cost reduction to move beyond niche thin-film applications. Click chemistry functionalization requires more economical reagents and streamlined purification protocols to achieve commercial viability.

**Table 4:** Summary of synthesis methods for polymer nanocomposites incorporating hybrid nanoparticles, including classification, processing principles, advantages, and limitations

Method	Type (approach)	Principle/process	Advantages	Disadvantages/limitations	References
Sol–gel method	Bottom-up	Chemical transformation of sol to gel *via* hydrolysis and polymerization of metal alkoxides or precursors	High purity and homogeneity; low processing temperature; good control over composition and structure; suitable for coating and film; simple and overall cost-effective	Time-consuming (gelation, drying); shrinkage and cracking; sensitive to temperature, pH, and concentration; possible impurity from incomplete reactions; uses inorganic solvents (environmental concern)	143–145
*In situ* polymerization	Bottom-up	Nanomaterials dispersed in the monomer; polymerization is initiated to form a nanocomposite in the continuous phase	Strong interfacial bonding; uniform nanoparticle dispersion; scalable; enhanced mechanical, thermal, and dielectric properties	Requires surface modification of nanoparticles; possible agglomeration; high viscosity complicates processing; potential equipment wear	146–148
Mechanical blending (ball milling/melt blending)	Top-down	Physical mixing of polymer and fillers using shear or grinding forces; solid-state or melt-based dispersion	Simple, cost-effective, and scalable; eco-friendly (no solvent); good filler dispersion; suitable for thermoplastics	Poor control over particle size; contamination from milling media; high energy and time consumption; possible degradation due to heat	149–151
Melt intercalation	Top-down	Filler layers (*e.g.*, clay) are intercalated/exfoliated in molten polymer through shear mixing at high temperature	Environmentally friendly; good filler dispersion without solvents; compatible with extrusion processes; industrially viable	Requires compatibilizers for nonpolar polymers; weak polymer–filler interactions; structural instability under shear; limited to thermoplastics	36, 38 and 152
Layer-by-layer assembly	Advanced	Sequential adsorption of oppositely charged species to build multilayered structures (immersive, spin, spray, and so on)	Precise control over thickness and composition; tunable surface properties; applicable to coatings and thin films; eco-friendly (water-based, possible)	Long preparation time; complex process; limited to thin films; possible non-uniform thickness; some methods use volatile or hazardous solutions	109, 153 and 154
3D printing (additive manufacturing)	Advanced	Layer-by-layer fabrication of composite structures using polymer-filler filaments (*e.g.*, CNTs, h-BN, graphene)	High-design flexibility; complex geometry possible; controlled filler distribution; strong mechanical and thermal properties; scalable for prototyping	High equipment cost, filler aggregation, weak interlayer adhesion, limited printable material types, and surface roughness issues	155–157
Microfluidic synthesis	Bottom-up/continuous-flow	Confined mixing in laminar microchannels using droplet reactors or flow-focusing geometries to control nucleation, growth, and hybrid nanoparticle assembly	Narrow particle size distribution; high reproducibility; on-chip functionalization; excellent control of mixing and residence time; suitable for shear-sensitive polymers	Channel fouling, limited throughput, requires microfabrication, and scaling up is challenging	125 and 126
Atomic layer deposition (ALD)	Vapor-phase, self-limiting	Alternating precursor pulses react with surface groups to deposit angstrom-level inorganic layers (Al_2_O_3_, TiO_2_, ZnO) on polymers or nanoparticles	Conformal ultrathin coating; precise shell thickness control; enhances dispersion and stability; prevents aggregation; improves barrier and thermal properties	Expensive equipment; slow deposition rates; thermal sensitivity of polymers; requires careful precursor control and sometimes UV activation	127 and 128
Click chemistry	Chemical functionalization	Covalent grafting of polymers or ligands onto nanoparticle surfaces through fast, orthogonal, mild click reactions, enabling tunable interfacial chemistry	Precise surface functionalization; excellent compatibility and dispersion; strong covalent interfacial bonding; RAFT-enabled polymer architecture control; works under mild conditions	Multi-step synthesis; copper residue may cause cytotoxicity; slower functionalization for large particles; higher reagent cost	130 and 132

A critical gap across all techniques is the lack of in-line characterization and feedback control. Real-time monitoring of particle dispersion, interfacial adhesion, and structure evolution during processing would enable adaptive process control, improving reproducibility and reducing defect rates.^94^ Future research may prioritize the development of such monitoring capabilities alongside continued refinement of synthesis methodologies. Additionally, life-cycle assessments comparing environmental impacts of different manufacturing routes are conspicuously absent from the literature, yet essential for guiding sustainable technology selection.^124^ Ultimately, advancing hybrid nanocomposite manufacturing requires integrating materials science insights with chemical engineering process design and industrial pragmatism. Techniques demonstrating laboratory promise should be evaluated not only on achievable properties but also on scalability, reproducibility, energy consumption, and total production cost to realize widespread adoption.

A critical constraint on the industrial translation of polymer nanocomposites containing hybrid nanoparticles often arises before matrix processing, namely during the synthesis of the engineered fillers themselves. Complex architectures such as Janus particles, multi-shell core–shell particles, and decorated heterodimers typically require sequential emulsion polymerization, seeded growth, selective surface passivation, masking, anisotropic etching, or multistep surface functionalization. These routes provide strong control over particle asymmetry and interfacial chemistry at the laboratory scale, but scale-up can reduce morphological fidelity, batch yield, and product purity.^133,134^

Janus particle synthesis is especially sensitive to scale. Masking and interface-confined functionalization methods, including Pickering emulsion templating, can generate well-defined anisotropic particles, but larger-scale production is limited by low volumetric productivity, batch-to-batch variation in the Janus balance, and purification losses associated with removing symmetric or incompletely functionalized particles.^135,136^ Microfluidic and electrohydrodynamic co-jetting approaches improve morphological control, but industrial throughput usually requires parallelized devices, robust clogging control, and specialized pumping and monitoring infrastructure.^136,137^

Multi-shell core–shell particles face cumulative yield and quality losses at each deposition step. Shell thickness uniformity, interlayer adhesion, drying-induced cracking, calcination damage, and shell delamination during melt processing can alter filler performance.^138,139^ Similarly, surface grafting, which is often required to improve polymer compatibility, adds cost and waste: grafting-to methods are limited by steric crowding, whereas grafting-from routes such as surface-initiated ATRP or RAFT require controlled reaction conditions, catalyst removal, and additional purification.^74,140^

These upstream limitations directly affect top-down nanocomposite processing. Variability in filler morphology, grafting density, and residual impurities can shift viscosity, dispersion quality, and percolation thresholds, complicating process qualification and reproducibility.^94^ Future progress will therefore require continuous-flow or intensified synthesis platforms, masterbatch-based pre-dispersion, in-line monitoring, and standardized reporting of filler lot characteristics together with final composite properties.^141,142^

## 4. Properties and functionalities of polymer nanocomposites with hybrid nanoparticles

### 4.1. Mechanical properties

**Fig. 12:**
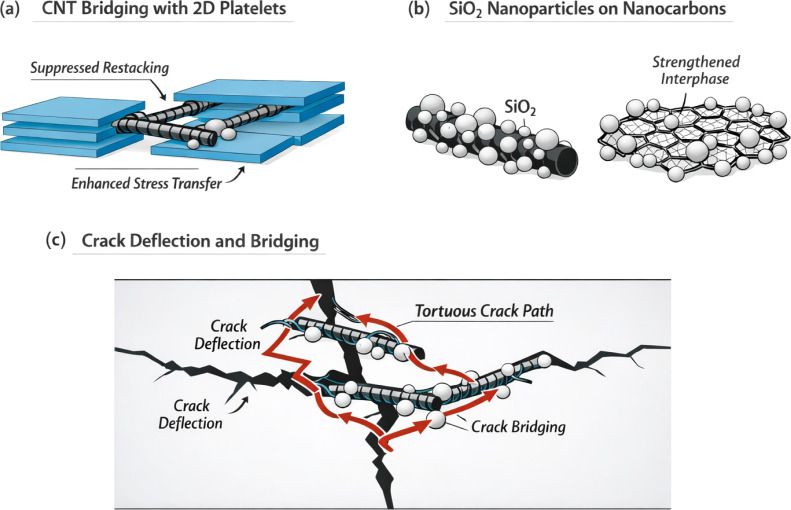
(a) 1D CNTs bridging between 2D platelets (suppressed restacking, improved stress transfer), (b) inorganic nanoparticles (SiO_2_) decorating or surrounding nanocarbons to strengthen the interphase, and (c) crack deflection/bridging on a schematic fracture surface with arrows indicating tortuous crack paths.

Hybrid nanofillers (for example, 0D–1D, 1D–2D, and nano-micro combinations) often improve stiffness, strength, and damage tolerance beyond those of neat polymers or single-filler nanocomposites, provided that co-dispersion and efficient interfacial stress transfer are simultaneously achieved.^26,158,159^ Synergistic reinforcement is most consistently observed when (i) one filler suppresses the characteristic aggregation mode of the other, such as CNT bundling or graphene restacking; (ii) a connected, load-bearing network forms at relatively low overall filler loading; and (iii) interfacial chemistry promotes shear transfer through a robust interphase rather than weak physical contact alone.^26,46,159^Fig. 12 schematically summarizes the dominant reinforcement mechanisms responsible for the mechanical synergy observed in polymer nanocomposites containing hybrid nanoparticles. As illustrated in Fig. 12a, 1D CNTs act as interlayer bridges between adjacent two-dimensional nanoplatelets, suppressing platelet restacking and enabling the formation of a connected, load-bearing network that facilitates efficient stress transfer at relatively low filler contents. Fig. 12b highlights the role of rigid inorganic nanoparticles, such as SiO_2_, which decorate nanocarbon surfaces or occupy interfacial regions, thereby strengthening the polymer-filler interphase and reducing local strain concentrations. Under mechanical loading (Fig. 12c), the hybrid architecture promotes crack deflection along nanoplatelet surfaces and nanotube-mediated crack bridging, thereby increasing crack tortuosity and delaying microcrack coalescence. The combined action of these mechanisms accounts for the simultaneous improvements in stiffness, strength, and fracture resistance for hybrid nanofiller systems.

In fiber-reinforced architectures, hybrid nanoparticles can enhance both load transfer to the fibers and matrix-dominated deformation. Sathish *et al.* demonstrated that combining MWCNTs with SiO_2_ in basalt/glass-fiber polymer composites improved tensile performance at optimized low filler contents, whereas higher loadings promoted agglomeration and diminishing gains, highlighting the need for careful composition and processing control.^160^ In multiscale bio-based systems, Champa-Bujaico *et al.* reported that ternary SEP (sepiolite) : MWCNT : WS_2_ (tungsten disulfide) reinforcement in poly(3-hydroxybutyrate) (P3HB)-based composites increased stiffness by up to 132%, with maximum stiffness gains occurring at 1 : 2 : 2 wt% SEP : MWCNTs : WS_2_, consistent with well-dispersed, percolated reinforcement networks.^161^ Tajdari *et al.* showed that ZnO content in PLA/ZnO/TiO_2_ nanocomposite films from 1.75 to 5.25 wt% increased tensile strength from approximately 32.5 to 38.5 MPa, tensile modulus from approximately 1360 to 1730 MPa, and elongation at break from approximately 1.30 to 1.60%, corresponding to increases of about 18.5%, 27.2%, and 23.1%, respectively.^162^

From a mechanistic perspective, 1D–2D carbon hybrids are frequently associated with synergistic stress sharing and crack-tip shielding *via* CNT bridging between adjacent nanoplatelets, crack deflection, and delayed microcrack coalescence (see Fig. 12). Mechanical performance typically peaks at intermediate CNT-to-platelet ratios rather than at maximum filler loadings.^46,159,163^ In epoxy systems, Jen *et al.* demonstrated that a MWCNT : GNP ratio of 1 : 9 delivered superior monotonic and fatigue performance by limiting nanoplatelet agglomeration and establishing efficient load-transfer pathways through CNT bridging.^209^ Hybrid architectures that combine nanocarbons with rigid inorganic nanoparticles can further strengthen the interphase and reduce strain localization; for example, fused-silica/MWCNT hybrids increased epoxy tensile strength and modulus by up to 12% and 37%, respectively, at an optimized composition (0.6 wt% of CNTs and 50 wt% of silica particles).^164^ Beyond bulk tensile properties, hybrid nanofillers can substantially enhance fracture resistance in laminated composites, as evidenced by a 120% increase in Mode I interlaminar fracture toughness reported for CFRP incorporating a 1 wt% GNP/MWCNT hybrid interleaf system.^165^ While these studies clearly establish the mechanical benefits of hybrid nanofillers, future research may prioritize scalable dispersion strategies such as masterbatching, controlled rheology during melt compounding, and stable surface treatments, alongside quantitative structure–property frameworks capable of distinguishing true synergy from additive effects. Mechanical characterization can be extended beyond quasi-static tension to include fracture, fatigue, and creep, where hybrid nanofiller networks often deliver their most practically relevant performance gains.^26,46,159,165^

### 4.2. Thermal properties

Neat polymers generally exhibit low thermal conductivity (*k* ≈ 0.1–0.5 W m^−1^ K^−1^), which limits their applicability in advanced thermal management systems. This behavior arises from inefficient phonon transport caused by intrinsic polymer chain disorder and, in filled systems, pronounced phonon scattering at polymer-filler interfaces. Hybrid nanofiller strategies address these limitations by integrating complementary nanostructures that promote continuous heat-conduction pathways, reduce interfacial thermal resistance, and preserve multifunctional performance.^166,167^ Significant enhancements have been reported using hybrid combinations of two-dimensional fillers. Ren *et al.* demonstrated that epoxy composites containing graphene-boron nitride (BN) nanosheet hybrids achieved a thermal conductivity of 0.649 W m^−1^ K^−1^ at only 5 wt% loading.^168,169^ This improvement was attributed to noncovalent π–π interactions at the graphene-BN interface, which enhanced phonon coupling across the heterointerface and suppressed phonon scattering. The synergistic integration of graphene's high intrinsic thermal conductivity with the electrical insulation of boron nitride enables composites suitable for thermally demanding electronic applications.

**Fig. 13:**
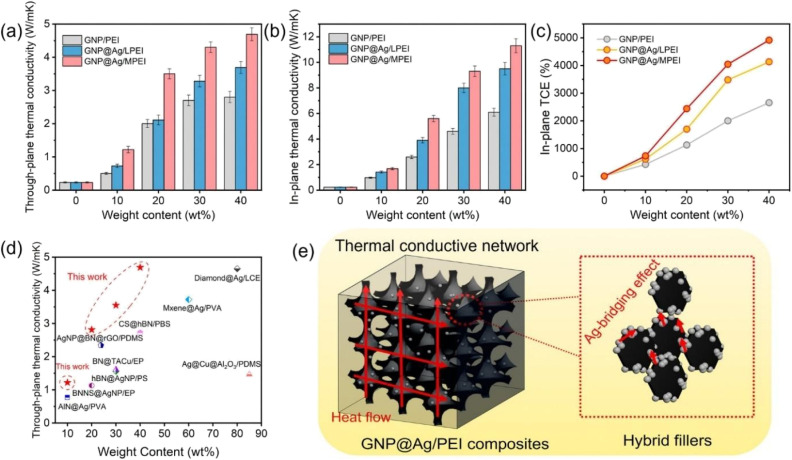
Thermal transport behavior of GNP@Ag-PEI composites: (a) through-plane thermal conductivity, (b) in-plane thermal conductivity, (c) thermal conductivity enhancement as a function of hybrid filler loading, (d) comparison of thermal conductivity with representative polymer nanocomposite systems, and (e) schematic illustration of the Ag-decorated graphene nanoplatelet (GNP@Ag) network within the PEI matrix, reproduced from ref. 173 with permission from American Chemical Society, copyright 2024 (License No. 6297030496310).

**Fig. 14:**
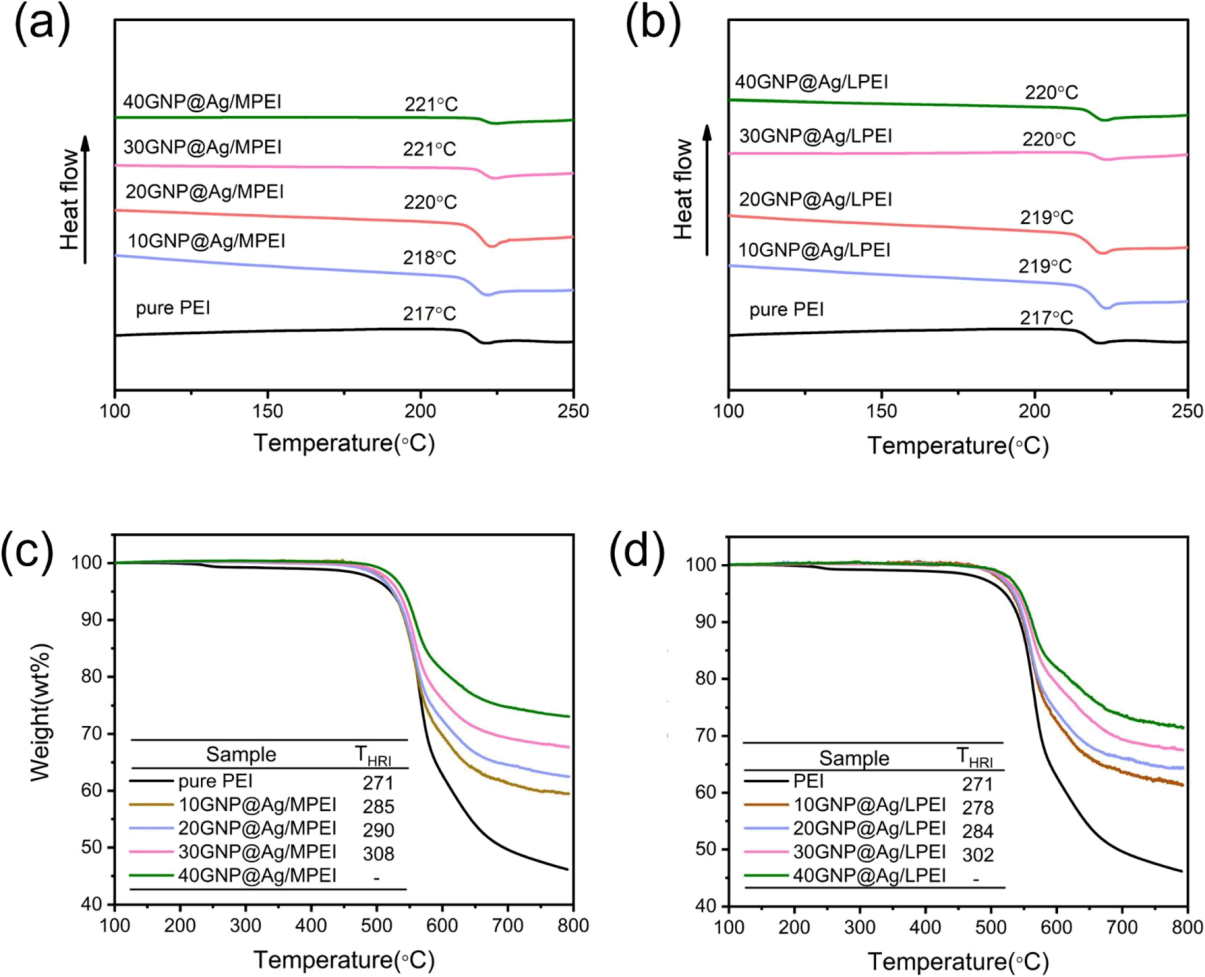
Thermal characterization of GNP@Ag hybrid nanofiller-reinforced PEI composites: DSC curves of (a) MPEI and (b) LPEI, and TGA curves of (c) MPEI and (d) LPEI composites, reproduced from ref. 173 with permission from American Chemical Society, copyright 2024 (License No. 6297030827524).

Hybridization strategies have also incorporated metallic nanoparticles to further enhance heat transport. Barani *et al.* reported epoxy composites reaching 13.5 ± 1.6 W m^−1^ K^−1^ using a combination of graphene (40 wt%) and Cu nanoparticles (35 wt%).^170^ In this system, Cu nanoparticles acted as thermally conductive bridges between graphene flakes, facilitating percolative heat transport and lowering interflake thermal resistance. Related studies on graphene-BN hybrids and segregated filler networks confirm that hybrid architectures enable concurrent control over thermal and electrical properties.^158,171,172^Fig. 13 illustrates the anisotropic thermal transport behavior of GNP@Ag/PEI (polyetherimide) composites, showing enhanced through-plane and in-plane thermal conductivities with increasing hybrid filler content, along with a schematic highlighting Ag-mediated thermal bridging between graphene nanoplatelets.^173^Fig. 14 depicts DSC (differential scanning calorimetry) and TGA (thermogravimetric analysis) curves of GNP@Ag-reinforced medium-molecular-weight polyetherimide (MPEI) and low-molecular-weight polyetherimide (LPEI) composites, revealing minimal changes in thermal transitions and improved thermal stability due to the barrier effect and heat-dissipation capability of the hybrid nanofillers.^173^ However, despite substantial progress, several challenges remain. Future efforts may focus on ensuring long-term thermal stability under cyclic and harsh operating conditions, optimizing 3D hybrid networks at moderate filler loadings to maintain mechanical integrity and processability, and developing scalable, cost-efficient manufacturing routes suitable for industrial deployment.^166,167^

### 4.3. Optical and electrical properties

Tailoring the optical and electrical properties of polymer nanocomposites is governed by the electronic structure of the inorganic phase, its spatial distribution, and the efficiency of charge transport across polymer–nanofiller interfaces. Hybrid nanoparticle systems provide distinct advantages by combining complementary functionalities, enabling concurrent control of optical absorption, dielectric response, and electrical conductivity. Core–shell and heterostructured nanoparticles are particularly effective because they stabilize dispersion, regulate interfacial electronic states, and introduce controlled pathways for carrier hopping or tunneling.^174^ In poly(3-hexylthiophene) (P3HT) films, the incorporation of 6 wt% ZnO@SiO_2_ core–shell nanoparticles resulted in an approximately 98% increase in absorbance and optical conductivity at a wavelength of 500 nm.^175^ This enhancement is attributed to improved light–matter interaction and more continuous carrier pathways enabled by the hybrid architecture, while the SiO_2_ shell suppresses nanoparticle aggregation and moderates interparticle charge transfer. Hybrid metal oxide systems further demonstrate the capacity for simultaneous bandgap tuning and dielectric enhancement. In hydroxypropyl methylcellulose/sodium alginate (HPMC/NaAlg) blends, the addition of 9 wt% CuO/TiO_2_ reduced the direct bandgap from 4.51 to 3.28 eV and the indirect bandgap from 3.37 to 2.29 eV.^176^ These changes were accompanied by a two-order-of-magnitude increase in AC conductivity, from 1.68 × 10^−12^ to 1.68 × 10^−10^ S m^−1^, and an increase in dielectric constant from 12 to 270 at 10 Hz. Such coupled behavior is characteristic of hybrid nanodielectrics, where interfacial polarization and defect-related electronic states contribute to enhanced permittivity and charge transport.^177,178^

**Fig. 15:**
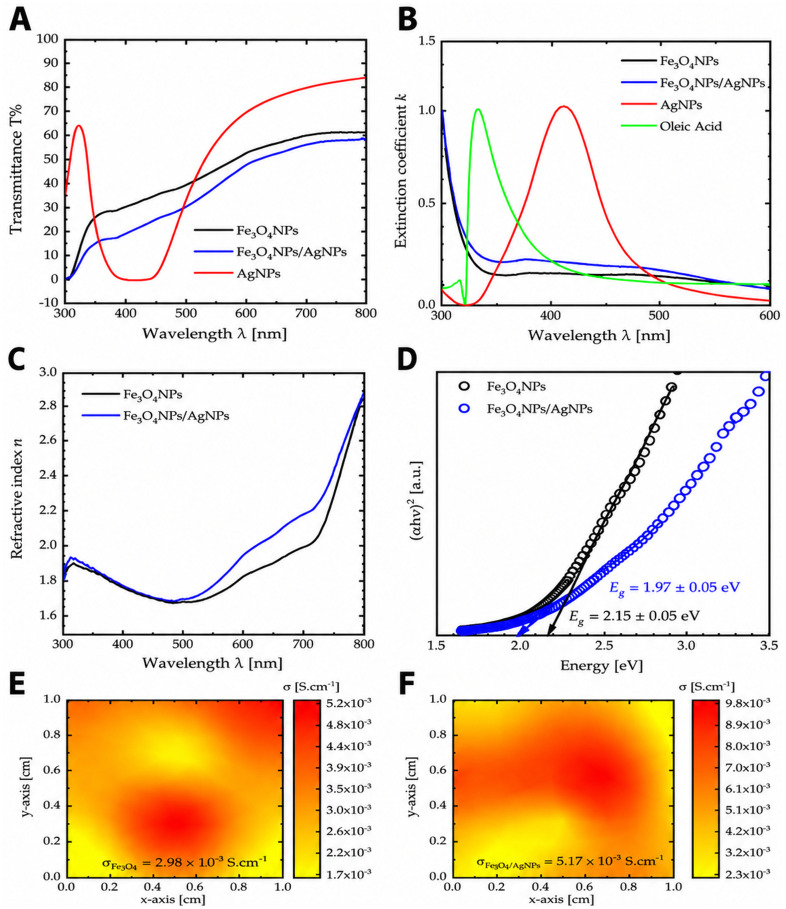
Comparison of Fe_3_O_4_/AgNPs hybrid nanoparticles with others: (a) transmittance spectra, (b) extinction coefficient spectra, (c) refractive index spectra, (d) Tauc plot for Fe_3_O_4_ and Fe_3_O_4_/AgNPs, and heatmap for electrical conductivity of (e) Fe_3_O_4_ NPs and (f) Fe_3_O_4_/Ag NPs, reproduced from ref. 180 with permission from Springer Nature, copyright 2024 (License No. 6297031252284).

Magnetic-plasmonic hybrid nanoparticles represent another important class of multifunctional fillers. Fe_3_O_4_/Ag hybrids consistently exhibit higher electrical conductivity than Fe_3_O_4_ alone, as the Ag component facilitates efficient electron transport and the formation of conductive networks, while the magnetic core contributes additional functional attributes.^179,180^ This combination has been widely explored in polymer nanocomposites designed for EMI shielding and related optoelectronic applications. Fig. 15a–d compare the transmittance, extinction coefficient, refractive index, and Tauc plots of Fe_3_O_4_ and Fe_3_O_4_/Ag nanoparticles, respectively, illustrating the modification of optical constants and bandgap behavior induced by Ag hybridization, whereas Fig. 15e and f demonstrate electrical conductivity heatmaps for Fe_3_O_4_ and Fe_3_O_4_/Ag nanoparticles, respectively, highlighting the superior conductivity of the hybrid system arising from improved charge transport pathways.^180^

### 4.4. Antimicrobial and bioactive properties

Hybrid nanoparticles can impart strong antimicrobial and antibiofilm functionality to polymer matrices when their composition, dosage, and interfacial presentation are carefully controlled. In such systems, efficacy typically arises from the combination of contact-mediated membrane disruption, reactive oxygen species generation, and, where applicable, localized delivery of bioactive agents, thereby reducing reliance on a single antibacterial pathway. A representative example is the GO-PEG (polyethylene glycol)/Ag hybrid platform reported by Han *et al.*, in which GO promotes bacterial capture *via* surface functional groups, while Ag nanoparticles induce membrane damage and oxidative stress.^181,182^ The same architecture enables co-delivery of antibiotics, producing a three-component mechanism: bacterial capture, puncture, and biochemical inhibition. This multifunctional approach improves antibacterial performance while potentially lowering required antibiotic dosage. Beyond Ag-GO systems, lipid–polymer hybrid nanoparticles (LPHNPs) enable co-encapsulation of conventional antibiotics with poorly soluble phytochemicals, supporting sustained exposure within the biofilm microenvironment. Mohammadi *et al.* demonstrated that curcumin-gentamicin LPHNPs suppress *Pseudomonas aeruginosa* biofilms more effectively than comparable single-carrier systems.^183^ The enhanced efficacy was attributed to improved penetration into the extracellular polymeric substance and coordinated release of both agents.

**Fig. 16:**
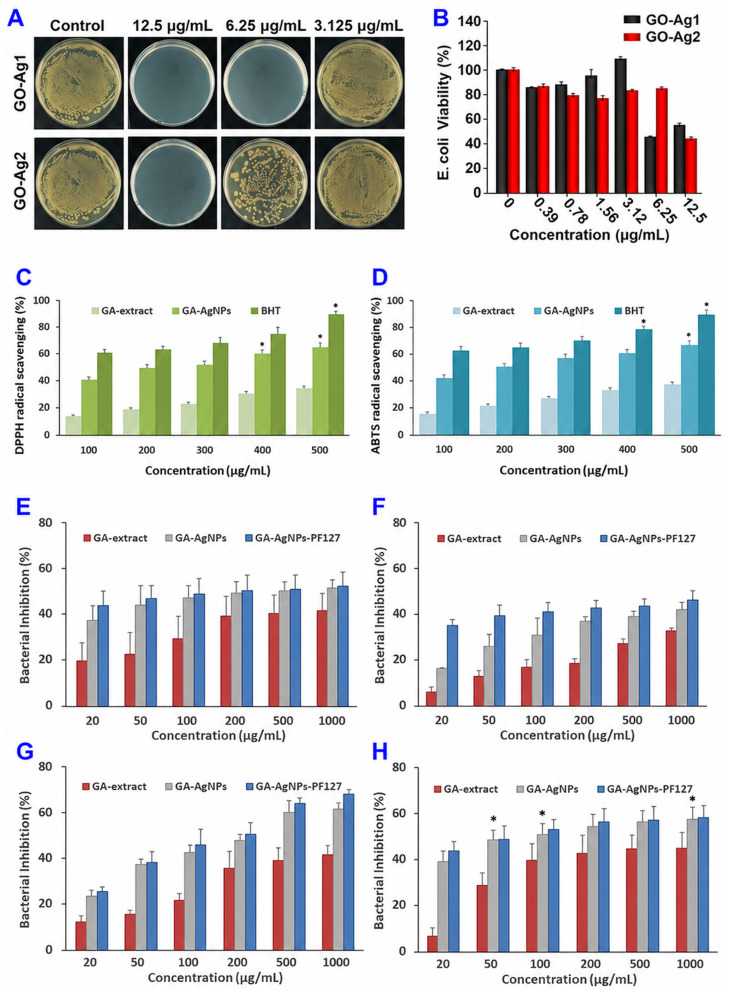
Antibacterial activity of GO-PEG/Ag nanocomposites: (A) colony formation of *E. coli* and (B) *E. coli* viability at different treatments, reproduced from ref. 181 under CC BY 4.0 license; antioxidant effects of GA-AgNPs showing (C) DPPH radical scavenging and (D) ABTS radical scavenging, and biofilm inhibition effect for varied concentrations of GA extract, GA-AgNPs, and GA-AgNPs-PF127 on (E) *B. cereus*, (F) *E. coli*, (G) *P. aeruginosa*, and (H) *S. aureus*, reproduced from ref. 185 under CC BY 4.0 license.

Complementary hybrid designs integrate carbon nanostructures with metal or metal-oxide nanoparticles to combine high surface contact with chemical reactivity. MWCNTs decorated with ZnO or Ag nanoparticles have been shown to enhance antimicrobial activity and inhibit biofilm formation against both Gram-positive and Gram-negative bacteria at relatively low loadings.^184^ Green-synthesized hybrids can further introduce antioxidant functionality relevant to wound-care environments, where infection and oxidative stress often coexist. Chandrasekharan *et al.* reported that *Gmelina arborea* (GA)-derived Ag nanoparticles reduced biofilm formation more effectively than the plant extract alone, with further improvement after formulation into a GA-AgNPs-PF127 hydrogel; DPPH and ABTS assays confirmed radical-scavenging activity.^185^ From a translational perspective, key design priorities include maintaining antimicrobial potency while controlling metal ion release and cytocompatibility, validating performance against mature biofilms, and monitoring the potential for resistance development in silver-containing systems. Fig. 16A and B demonstrate the antibacterial activity of GO-PEG/Ag nanocomposites through reduced *E. coli* colony formation and viability,^224^ while Fig. 16C and D show the antioxidant activity of GA-AgNPs evaluated using DPPH and ABTS radical-scavenging assays^228^ and Fig. 16E–H compares concentration-dependent biofilm inhibition by GA extract, GA-AgNPs, and GA-AgNPs-PF127 against *B. cereus*, *E. coli*, *P. aeruginosa*, and *S. aureus*.^228^

### 4.5. Magnetic properties

**Fig. 17:**
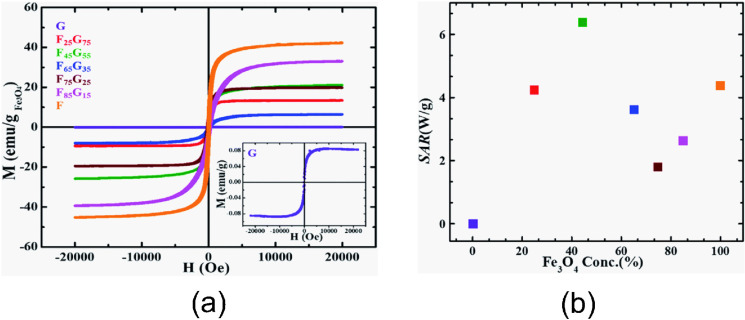
Composition-dependent magnetic hysteresis and hyperthermia performance of Fe_3_O_4_/graphene nanohybrids: (a) room temperature hysteresis loops for compositions F25G75 to F85G15 normalized by sample mass and mass of the magnetic component (Fe_3_O_4_); inset shows hysteresis loop of thermally reduced pure graphene; and (b) specific absorption rate *versus* Fe_3_O_4_ content measured at 633 kHz (9.1 mT), showing optimal performance at 45 wt% Fe_3_O_4_ (SAR of 6.45 W g^−1^), reproduced from ref. 190 under CC BY-NC 3.0 license.

Magnetically responsive hybrid nanoparticles enable field-guided localization, imaging, and heat-mediated therapies. Core–shell architectures, such as Fe_3_O_4_ cores with polymeric shells, can be directed by external magnetic fields to concentrate payloads at target sites, while surface coatings improve colloidal stability, reduce aggregation, and enable functional bioconjugation for biomedical use.^186–188^ Under alternating magnetic fields, localized heating arises primarily from Néel and Brownian relaxation losses, and, in some regimes, minor hysteretic contributions, forming the basis of magnetic hyperthermia when heating is confined to therapeutically relevant temperature windows.^187–189^ In polymer matrices, hybrid magnetic fillers provide an effective route to tailor bulk magnetization and hysteresis behavior. Kaadhm *et al.* reported that epoxy composites reinforced with 15 wt% Fe_3_O_4_ and 2 wt% Ni achieved a saturation magnetization of 17.2 emu g^−1^, remanence of 1.8 emu g^−1^, and coercivity of 99.09 Oe, demonstrating that compositional hybridization can introduce meaningful magnetic response and controlled coercivity into otherwise weakly magnetic polymers.^186^ For hyperthermia-oriented hybrids, magnetic performance depends strongly on interfacial coupling and percolation effects. Seal *et al.* showed that incorporating amine-functionalized MWCNTs into Fe_3_O_4_ nanocomposites enhances magnetic saturation and specific absorption rate to an optimal CNT fraction, whereas excessive loading leads to magnetic decoupling and network-induced attenuation of magnetization.^187^ Consequently, heating efficiency is maximized by balancing CNT content, dispersion quality, and interfacial affinity. Fig. 17 schematically illustrates the influence of hybrid filler composition on magnetic hysteresis behavior and hyperthermia performance. As reported by Dar *et al.*, Fe_3_O_4_/graphene nanocomposites exhibit a pronounced composition dependence in both magnetic hysteresis (Fig. 17a) and hyperthermia efficiency (Fig. 17b), with an optimal specific absorption rate (SAR) of 6.45 W g^−1^ achieved at 45 wt% magnetite, approximately 1.5 times higher than that of pure Fe_3_O_4_. This intermediate composition reflects an optimal balance between magnetic coupling efficiency and dipolar interaction suppression, while also benefiting from the enhanced thermal capacity and heat dissipation provided by graphene.^190^ Accordingly, best-practice design strategies emphasize the co-engineering of particle anisotropy, hydrodynamic size, and surface chemistry, alongside the careful selection of alternating magnetic field parameters that comply with established clinical safety limits.^188,189^

### 4.6. Barrier and permeability properties

Hybrid nanoparticles provide a versatile route to regulate molecular transport across both biological and non-biological barriers by jointly tuning diffusivity (*D*) and solubility (*S*), and therefore the overall permeability (*P* = *D* × *S*). In barrier nanocomposites, impermeable high-aspect-ratio domains primarily lower *D* by enforcing tortuous diffusion pathways, while the polymer-filler interphase can further reduce segmental mobility and free-volume-mediated transport. Hybridization (*e.g.*, nano-micro or 0D-1D–2D combinations) is especially effective when secondary nanostructures suppress platelet restacking/aggregation, bridge interfacial voids, and homogenize dispersion, thereby reducing defect-mediated leakage at a given filler loading and enabling a closer approach to model-predicted barrier limits.^191,192^

In central nervous system delivery, LPHNPs combine the mechanical stability of polymeric cores with lipid interfaces that support PEGylation and ligand presentation, enabling receptor-mediated interactions at the blood–brain barrier (BBB) and controlled release of encapsulated therapeutics.^193^ Beyond architecture alone, lipid composition and surface interactions are increasingly recognized as decisive for brain uptake; for example, low-density lipoprotein receptor-mediated pathways and related corona-mediated effects can enhance brain accumulation and intratumoral penetration.^194^

**Fig. 18:**
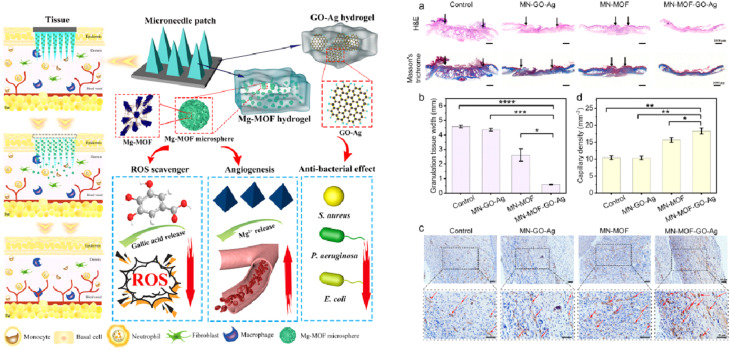
Hybrid nanoparticle microneedle for diabetic wound healing: (i) schematic of MN-MOF-GO-Ag with Mg-MOF-loaded dissolving tips (Mg^2+^/gallic acid release) and a GO-Ag backing layer (antibacterial function); and (ii) day 17 therapeutic outcomes showing (a) H&E staining (upper row) indicating granulation tissue formation and Masson's trichrome staining (lower row) showing collagen deposition across treatment groups, (b) quantification of granulation tissue width, (c) CD31 immunohistochemistry showing blood vessel formation, and (d) quantification of capillary density, reproduced from ref. 196 with permission from American Chemical Society, copyright 2021 (License No. 6297060176940).

Transdermal delivery provides a complementary route to bypass the stratum corneum, particularly when hybrid nanocarriers are integrated into dissolving microneedle platforms. Zhao *et al.* developed an LPHNP-loaded dissolving microneedle patch that delivers miR-218 into the skin and promotes hair regrowth by activating Wnt/β-catenin signaling, illustrating how minimally invasive devices and hybrid nanocarriers can be co-designed to overcome barrier-limited bioavailability and nucleic acid instability.^195^Fig. 18 schematically summarizes a related multifunctional microneedle architecture (MN-MOF-GO-Ag) reported by Yin *et al.*, in which Mg-MOF-loaded dissolving tips release Mg^2+^ ions and gallic acid while a GO-Ag backing layer provides antibacterial activity. Histological assessment at day 17 showed reduced granulation tissue width, improved and more oriented collagen deposition, and increased CD31-positive capillary density, yielding 88% wound closure compared with 26% in untreated diabetic controls.^196^ For non-biological barriers relevant to packaging and protective coatings, hybrid nanoparticles can concurrently enhance UV shielding and gas/moisture barrier performance. Gasti *et al.* showed that chitosan/pullulan films containing clove-oil-loaded chitosan-ZnO hybrid nanoparticles increased UV blocking by 6.16%, improved water-vapor barrier performance by 84.64%, and improved oxygen barrier properties by 57.66% relative to neat films.^197^ Such gains are consistent with synergistic contributions from (i) impermeable inorganic domains that increase diffusion tortuosity and (ii) interphase-mediated densification/immobilization that suppresses permeant sorption and diffusion, while simultaneously enabling multifunctionality (*e.g.*, UV screening and antimicrobial protection).^191,192^ Progress in this area will benefit from quantitative structure-transport correlations that couple permeability measurements to dispersion state, filler orientation, and interphase chemistry, ideally under application-relevant humidity and temperature conditions. In biomedical settings, translation further requires systematic evaluation of how hybrid nanocarriers influence barrier integrity, biodistribution, and immunological responses, alongside standardized reporting of delivery efficiency and safety-relevant endpoints.

### 4.7. Flame retardancy

**Fig. 19:**
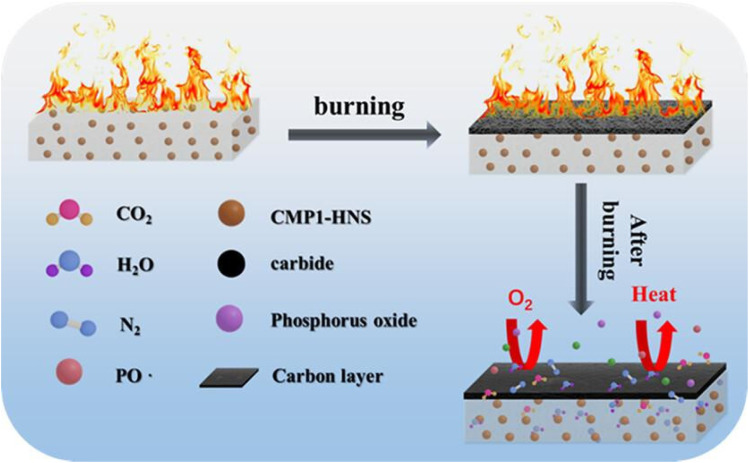
Flame retardant mechanism of CMP1-HNS-BSb/epoxy composites, reproduced from ref. 200 under CC BY-NC-ND 4.0 license.

Hybrid nanofillers enhance fire safety by increasing the limiting oxygen index (LOI), suppressing peak heat-release rate (pHRR), and promoting cohesive, thermally insulating char. These gains commonly arise from synergistic action across condensed- and gas-phase pathways, including barrier formation that restricts heat and mass transfer, catalytic charring that accelerates carbonization and strengthens char integrity, and suppression or purification of combustible volatiles, often accompanied by reduced smoke and toxic-gas release.^198–200^ Such multi-modal behavior is consistent with the established understanding of hybrid nanomaterial-flame retardant systems in polymer matrices.^201^ In epoxy, Hong *et al.* prepared Ag within halloysite nanotubes (HNT) and a polyphosphazene (PZE) coating on Ag@HNT surfaces (Ag@HNT@PZE) hybrids that increased LOI by 33.6% and reduced pHRR by 34.2%, attributed to catalytic char formation and purification of evolved gases enabled by the polyphosphazene-functionalized hybrid architecture.^198^ For cellulosic substrates, Li *et al.* developed amine-functionalized SiO_2_ hybrids incorporating Schiff-base structures, delivering a 27.6% increase in LOI and a 34.2% reduction in pHRR while imparting durable antibacterial activity, indicating a multifunctional protection strategy relevant to textiles and packaging.^199^ In polyurethane coatings, ZnO/phytic-acid core–shell hybrids reduced the flame-spread rate by 50% without degrading mechanical performance, consistent with phosphorus-rich char formation from phytic acid and ZnO-assisted stabilization of the protective layer.^202^ Ma *et al.* further demonstrated efficacy at ultra-low loading: 0.5 wt% conjugated microporous polymers hollow nanospheres coated with antimony pentoxide solution (Sb_2_O_5_) and bisphenol A-bis (diphenyl phosphate) (BDP) (CMPs-HNS-BSb) reduced pHRR by 21% and total heat release by 29.7% in epoxy, where hollow conjugated-microporous polymer nanospheres improved thermal insulation and suppressed melt dripping.^200^Fig. 19 illustrates the CMP1-HNS-BSb/epoxy flame-retardant mechanism, emphasizing how hierarchical hybridization promotes compact char formation and reduces heat and volatile transfer during combustion.^200^

### 4.8. Smart and stimuli-responsive properties

“Smart” polymer nanocomposites convert external stimuli (light, magnetic or electric fields, heat, mechanical stress/strain, or chemical triggers) into programmable, measurable changes in properties such as shape recovery, stiffness, conductivity, permeability, adhesion, and barrier or fire-protection performance. Hybrid nanoparticles, which integrate two or more nanoscale building blocks within a single filler architecture (for example, 0D magnetic nanoparticles coupled with 2D graphene or MXene, CNT-graphene hierarchical networks, or core–shell Fe_3_O_4_@polydopamine), are especially effective because they provide (i) multiple stimulus-transduction routes (photothermal, magnetothermal, electrothermal) for remote and localized activation, (ii) hierarchical conductive and thermal pathways that reduce percolation thresholds and remain operative under deformation, and (iii) chemically active interfaces (π–π interactions, hydrogen bonding, metal–ligand coordination, catechol chemistry) that support reversible interphase reconstruction and functional recovery beyond simple crack closure.^203–205^

Self-healing is a flagship function, but in hybrid-filled systems, it is most usefully framed in terms of stimulus delivery, energy routing, and interphase reformation. The main healing paradigms (physical, chemical, and supramolecular) and the intrinsic trade-off between chain mobility and mechanical robustness are well established.^206,207^ Hybrid nanofillers can enhance intrinsic repair by concentrating energy at damaged regions where bond exchange and chain diffusion are rate-limiting, using near-infrared photothermal heating, inductive magnetothermal heating under alternating magnetic fields, or electrothermal Joule heating. In parallel, hybrid filler surfaces can behave as reversible interphase nodes, enabling recovery of both mechanical integrity and electrical functionality after damage, which is essential for repeatable repair in coatings, soft devices, and stretchable electronics where bulk heating is impractical.^207–209^

Hybrid architectures are similarly central to shape-memory and multi-stimulus actuation, where remote triggering and programmable deformation sequences are required. Electro- and magneto-active shape-memory polymer nanocomposites rely on fillers that introduce alternative activation pathways and field-tunable recovery behavior.^203^ Distributed hybrid networks (*e.g.*, Fe_3_O_4_/graphene or CNT/metal frameworks) can convert magnetic, optical, or electrical inputs into localized heating along percolated pathways, enabling rapid, spatially resolved shape recovery that is difficult to achieve with single-component fillers. Coupled photothermal and magnetic responses further support reversible reconfiguration and shape-memory-assisted crack closure, which is attractive for soft robotics and reconfigurable device concepts.^204,205^

Beyond healing and actuation, percolated hybrid networks enable self-sensing and self-reporting. Hierarchical conductive architectures (for example, CNTs bridging graphene sheets) stabilize conductive pathways under cyclic strain and generate robust piezoresistive signals with tunable gauge factors, enabling real-time tracking of deformation, damage, and recovery, particularly when paired with dynamic matrices that restore conductivity upon healing.^210^ In safety-critical contexts, fire exposure can be treated as an extreme thermal stimulus that triggers adaptive protection *via* char formation and barrier-layer evolution. Evidence from flame-retardant polymer nanocomposites shows that combining nanomaterials with conventional flame retardants can yield synergistic condensed-phase and gas-phase mechanisms, strengthening the broader concept of “smart” response under severe stimuli.^201,211^

**Fig. 20:**
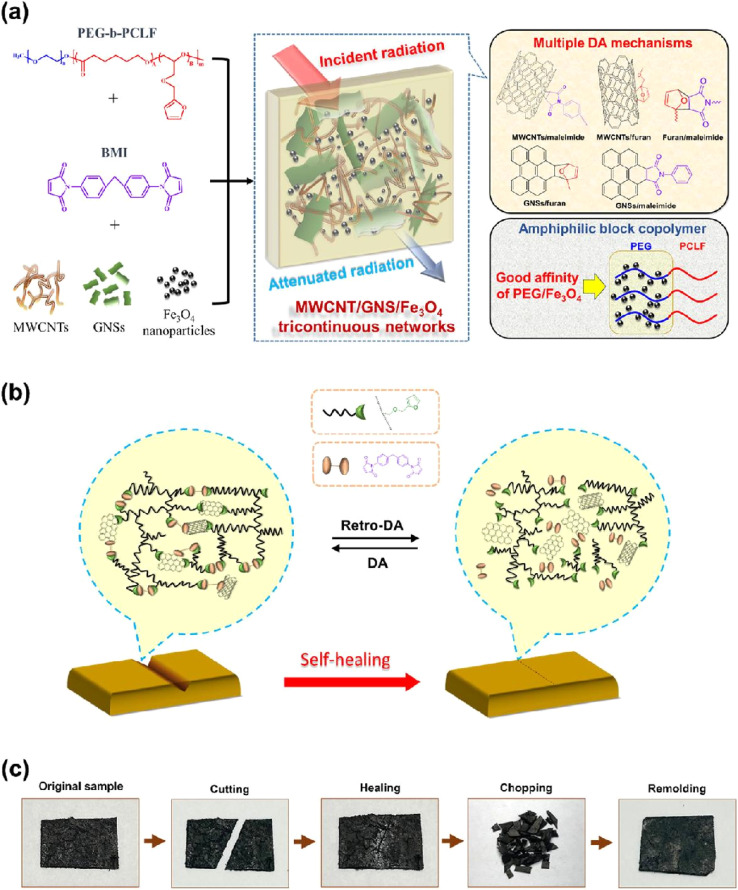
Design and self-healing mechanism of an MWCNT/GNS/Fe_3_O_4_ tricontinuous network nanocomposite: (a) synthetic scheme showing PEG-b-PCLF undergoing multiple Diels–Alder reactions with BMI and carbon nanomaterials, with MWCNTs bridging GNSs and Fe_3_O_4_ nanoparticles uniformly interspersed; (b) thermal self-healing showing heating triggers *retro*-DA de-cross-linking and molecular mobility for damage repair, while cooling reforms DA bonds and restores integrity; and (c) remolding practical demonstration showing cyclic cut-heal-remold capability, reproduced from ref. 212 with permission from American Chemical Society, copyright 2022 (License No. 6297060839892).

Self-healing in hybrid-nanoparticle nanocomposites can be classified by activation pathway and by the role of nanofillers in damage repair. Thermally activated systems commonly employ dynamic covalent chemistries that undergo controlled scission and reformation. Fig. 22 illustrates a tricontinuous network in which amphiphilic PEG-*b*-PCLF (poly(ethylene glycol)-*block*-poly(ε-caprolactone)) block copolymers form multiple Diels–Alder (DA) adducts with BMI (bismaleimide) cross-linkers and surface-functionalized carbon nanomaterials, yielding a dense DA-bonded architecture (Fig. 20a). Upon heating, *retro*-DA reactions transiently de-crosslink the network (Fig. 20b), increasing segmental mobility and enabling constituents to diffuse into the damaged zone. Cooling then restores DA cross-links, re-establishing network integrity and recovering mechanical performance. This reversible chemistry supports both crack healing and bulk remolding through repeated cut-heal-reshape cycles (Fig. 20c), demonstrating reprocessability alongside autonomous repair.^212^

**Fig. 21:**
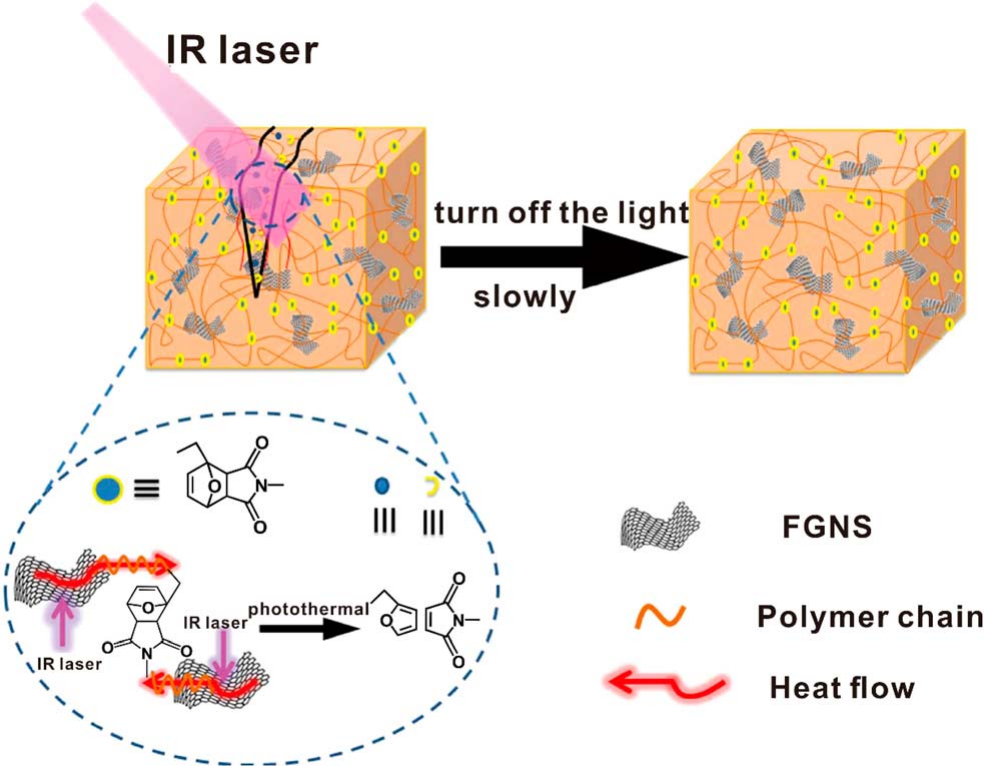
IR-laser-triggered photothermal self-healing in a functionalized graphene nanosheet/polyurethane nanocomposite, showing graphene converting photon energy to localized interfacial heat, enabling polymer chain interdiffusion and crack closure; subsequent cooling restores entanglements and interfacial bonding without external pressure, enabling remote, non-contact healing, reproduced from ref. 213 under CC BY 4.0 license.

Stimulus-responsive filler networks provide an alternative route by converting external energy inputs into localized interfacial heating. Fig. 21 shows photothermal self-healing in functionalized graphene nanosheet/polyurethane nanocomposites. Infrared irradiation is absorbed by the conductive graphene network and converted to heat *via* non-radiative relaxation, resulting in a localized temperature rise at the crack interface rather than uniform bulk heating. This confined heating promotes chain mobility, interdiffusion across the damage plane, and crack closure. After irradiation ceases, cooling enables re-entanglement and interfacial rebonding without external pressure, highlighting the dual role of conductive nanofillers as reinforcements and remotely addressable heating elements.^213^Table 5 compares polymer nanocomposites incorporating hybrid nanofiller systems, highlighting nanoparticle size and loading, functional roles, and the resulting enhancements in mechanical performance, thermal conductivity, and electrical or dielectric properties.

**Table 5:** Summary of polymer nanocomposite incorporating hybrid nanofiller systems, detailing nanoparticle size and loading, functional roles, and the resulting enhancements in mechanical performance, thermal conductivity, and electrical or dielectric properties^a^

Polymer matrix/hybrid nanofillers	NP size and loading	Functional roles of hybrid systems	Mechanical performance	Thermal transport	Electrical/dielectric performance	Notes/other key outcomes	References
Epoxy/graphene nanoplatelets (GNPs)/silica	GNPs: ∼2 nm thick, ∼4.5 µm lateral size; GNP loading explored up to ∼2 wt% (percolation ∼2.2 wt%)	Silica: disrupts electrical percolation (insulating “spacer”); GNPs: heat-conducting network; hybrid: decouples k enhancement from electrical conduction	Strength: max ∼81 MPa (+16%) at low GNP loading; modulus: ∼12.7–14.7 GPa	k: up to ∼1.54–1.56 W m^−1^ K^−1^ (≈+58% *vs.* baseline), stable over cryogenic-to-room range; processing route affects performance	Volume resistivity: >10^15^ Ω cm at/under low GNP (≤∼2 wt%); dielectric breakdown up to ∼22 kV mm^−1^ at low GNP (≤∼0.25 wt%)	High viscosity at higher GNP; porosity/dispersion strongly impacts insulation and breakdown	214
PVA/Ag/TiO_2_	Composite particles ∼150–200 nm (Ag addition increases average size to ∼200 nm). Synthesis masses: PVA/TiO_2_ (200 mg/200 mg) and PVA/Ag/TiO_2_ (200 mg PVA, 200 mg TiO_2_, 80 mg Ag)	Ag: SPR + e^−^ transfer/Schottky barrier; TiO_2_: Photocatalytic scaffold; PVA: Stabilizer/binder (reduces recombination, improves handling)	—	No bulk k reported; thermally stable under low-T synthesis conditions	Not intended as an electrical insulator; Ag provides conductive/charge-transfer pathways (dielectric metrics are not the focus of this study)	Optical bandgap reported ∼2.9 eV; heavy-metal ion removal optimized under UV-C irradiation (*e.g.*, Cr^6+^ up to ∼94%)	215
PMMA/ZnO/GO	Hybrid nanofiller loading: 0.1–0.4 wt% (optimum ∼0.2 wt%); composite nanostructure ∼200 nm	ZnO: antimicrobial (ROS, Zn^2+^ release); GO: dispersant + reinforcement; hybrid: synergistic antibacterial + mechanical gains	Flexural strength: +23.4%; compressive strength: +31.1% (optimum ∼0.2 wt%); declines above ∼0.3 wt% due to agglomeration	—	—	Lower water solubility/absorption; strong antibacterial efficacy (>60% reduction), non-cytotoxic; aging stability	216
PVDF/BaTiO_3_/MWCNTs	BaTiO_3_ ∼20 nm; MWNT dia. ∼20 nm; optimum ≈11.5 vol% BaTiO_3_ + 0.35 vol% MWNTs	MWNTs: Percolation-assisted polarization → high *ε*_r_; BaTiO_3_: high-k ceramic + “spacer” controlling conduction; hybrid: Promotes PVDF β-phase and suppresses losses	—	—	*ε*_r_ ∼59 (@100 Hz); tan *δ* < 0.055; E_bd_ ∼324 MV m^−1^; energy density ∼10.3 J cm^−3^	Stability at elevated temperature	217
PDMS/AlN + Al_2_O_3_ + BNNT (CAPA (catechol-polyamine)-modified)	BNNTs: 30–50 nm dia., length ∼10 µm. BNNT (neat or CAPA): 1 wt%; total inorganic filler concentration reported as 87 wt%	BNNTs bridge microfiller “gaps” → phonon pathways; CAPA improves BNNT dispersion and interfacial coupling; hybrid also improves flame resistance *via* char + radical trapping	Flexibility/robust thermal interface material behavior)	k: PDMS 0.19 → AlN/Al_2_O_3_ composite 5.5 → +BNNT-CAPA 8.1 W m^−1^ K^−1^	Electrically insulating BNNT-based thermal interface material concept (dielectric constants not emphasized)	LOI: 20.8% (PDMS) → 58.97% (AlN/Al_2_O_3_) → 64.77% (BNNT-CAPA). T_m_: 330.89 °C → 395.75 °C → 434.29 °C (BNNT-CAPA adds +38.54 °C *vs.* AlN/Al_2_O_3_)	218
PEG/GO (PEGylated GO; GO-PEG-curcumin (cur))	GO ∼1.7 nm; GO-PEG ∼3.5 nm; GO-PEG-Cur ∼11.5 nm; cur ∼4.5 wt%	PEGylation: colloidal stability/stealth; GO: carrier; hybrid: pH-responsive sustained drug delivery	—	Thermal stability improved after PEGylation (reduction peak shifted ∼175 → ∼202 °C)	—	Drug release: ∼60% at pH 7.4 and ∼50% at pH 5.5 over 96 h; zeta potential ∼−13.9 mV	219
Chitosan/ZnO + AgNPs/citronella essential oil (CEO)	AgNPs size ∼30 nm. (formulations (solvent casting): 0.66 g chitosan/100 mL; ZnO 0.33–0.99 g in 15 mL water; AgNP solution 10–30 mL; CEO fixed at 1 mL (series-dependent)	ZnO + AgNPs: broad-spectrum antimicrobial; CEO: hydrophobicity + antifungal contribution; hybrid: Synergy in microbial inhibition and barrier improvement	—	Thermal stability improved; *e.g.*, CZA series shows T10 ∼126–231 °C depending on composition	—	Barrier: WVP (water vapor permeability) decreases *vs.* control (best reported ≈1.026 ± 0.086 ×10^−10^ g Pa^−1^ m^−1^ s^−1^ for CZA5; control 1.411 ± 0.011 ×10^−10^). Optical: strong UV-blocking/opacity; SEM shows dispersion-crack trade-offs affecting WVP	220

a PMMA: polymethyl methacrylate, PVDF: polyvinylidene fluoride, PDMS: polydimethylsiloxane, and BNTs: boron nitride nanotubes.

## 5. Applications of polymer nanocomposites with hybrid nanoparticles

### 5.1. Aerospace and automotive

**Fig. 22:**
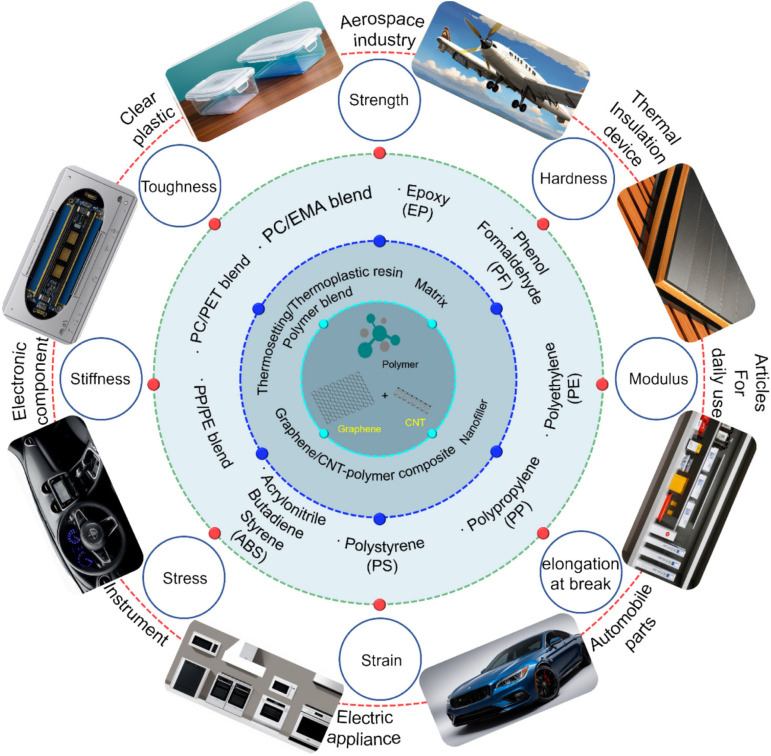
Various applications of graphene/CNT-hybrid polymer composites, reproduced from ref. 54 under CC BY 4.0 license.

Hybrid nanoparticle polymer nanocomposites, combining two or more nanoscale reinforcements within a polymer matrix (*e.g.*, ceramic nanoparticles with carbon nanostructures), are increasingly adopted to meet the coupled demands of aerospace and automotive structures. These sectors require lightweight materials with high mechanical reliability, improved damage tolerance, and added functionality, including electrical and thermal transport, surface protection, and durability under aggressive service environments. Hybridization across multiple length scales (0D/1D/2D) can improve stress transfer, suppress crack propagation, and introduce percolating pathways for charge or heat transport, provided that dispersion and interface quality are carefully controlled.^221–223^ Various applications of graphene/CNT-hybrid polymer composites are shown in Fig. 22.

Beyond structural metrics, hybrid carbon nanofiller systems are particularly relevant for applications that require electrical conductivity, such as lightning-strike protection (LSP), electrostatic discharge mitigation, and electromagnetic interference shielding in carbon-fiber-reinforced polymer components. By combining 2D GNPs (graphene nanoplatelets) with 1D SWCNTs, an interconnected conductive network can form at lower total filler content than single-filler systems, while maintaining processability for resin transfer molding and related manufacturing routes. For example, RTM6 epoxy filled with GNP/SWCNT mixtures (ratios 2 : 8, 5 : 5, 8 : 2) was reported to form a 3D conductive network that enhances the electrical conductivity of the composite and supports spray processing.^224^ More generally, GNP-CNT hybridization can reduce the percolation threshold and enable tunable conductivity, although the magnitude of synergy depends strongly on filler aspect ratio, dispersion state, and junction resistance.^221,222^

For LSP specifically, conventional expanded metal foils and meshes remain effective but add weight and can complicate manufacturability and durability. Carbon-based strategies, including CNT sheets, conductive veils, and hybrid graphene/CNT interlayers, are therefore being explored as lower-mass alternatives or supplements to metallic approaches.^225–228^ Reported LSP concepts include direct integration of CNT films within laminates, as well as copper-coated CNT interlayers tailored for co-curing with aerospace-grade prepregs.^225–227^ Recent perspectives also emphasize that scalable processing, consistent conductivity across large areas, and robust bonding to composite substrates are central requirements for next-generation LSP systems based on carbon nanomaterials.^228^

Hybrid fillers are equally impactful in joining and thermal management, where adhesives and primers must provide load transfer while reducing thermal resistance at bonded interfaces. A representative example is polyurethane adhesive and primer systems containing hexagonal boron nitride (hBN) nanoparticles and MWCNTs, where hBN provides high thermal conductivity while remaining electrically insulating, and a small CNT fraction can improve stiffness and strength and, in some cases, tailor electrical behavior depending on loading and network formation.^229^ The current thermal-management literature emphasizes that achieving high through-thickness heat transport in polymer composites typically requires engineered filler architectures (*e.g.*, alignment, 3D networks, or segregated structures) and low interfacial thermal resistance, especially for electrically insulating, thermally conductive applications relevant to transportation electrification and aerospace electronics packaging.^230,231^

Finally, tribological durability and surface functionality are key in automotive and aerospace subsystems (*e.g.*, bearings, bushings, seals, and sliding interfaces), where polymer composites, such as PEEK-based hybrids, continue to be developed to improve wear resistance under both dry and lubricated conditions.^232^ In addition, hybrid ceramic nanoparticle–carbon nanostructure coatings have attracted attention for surface protection, including anti-icing and corrosion resistance. For example, PVDF-HFP/SiO_2_/CNT nanocomposite coatings have been reported to exhibit strong water repellency and delayed icing, with performance linked to micro- and nanoscale roughness, surface chemistry, and coating robustness under practical handling.^233^Table 6 presents the representative polymer nanocomposites with hybrid nanoparticles for aerospace and automotive applications.

**Table 6:** Representative polymer nanocomposites with hybrid nanoparticles for aerospace and automotive applications

Composition (polymer + hybrid nanoparticles)	Synthesis/fabrication method	Aerospace and automotive-relevant applications	References
Epoxy resin + Al_2_O_3_/graphene nanoplatelets (GNPs)	Ultrasonication, hand lay-up, Compression molding	Aeronautical structures (*e.g.*, wings, fuselage, landing gear) with enhanced tensile strength (230 MPa with 3 wt% Al_2_O_3_) and hardness	234
RTM6 epoxy + GNPs (AO-4) + SWCNTs (ratios: 2 : 8, 5 : 5, 8 : 2)	Ultrasonication, resin transfer molding (RTM)	Enhanced thermal/electrical conductivity, EMI shielding, lightweight thermal management films in spacecraft/aircraft	224
Epoxy (bisphenol-A) + MWCNTs + SiO_2_ + core–shell rubber (CSR)	Two-step curing (85 °C/0.5 h + 120 °C/2 h)	Lightweight pressure vessels (aerospace), impact-resistant EV battery casings (automotive) with 218% toughness boost and 0.00163 S m^−1^ conductivity	235
PVDF-HFP + SiO_2_-CNT hybrid (superhydrophobic coating)	Solution blending and coating deposition	Anti-icing, corrosion resistance, and surface protection for aerospace external surfaces	236
Polyurethane + 0.5% hBNNPs + 0.25% MWCNTs	Mechanical mixing	High tensile strength adhesive joints for aerospace composites	229

### 5.2. Construction

Hybrid-nanoparticle polymer nanocomposites are increasingly used in construction to combine rapid curing with enhanced durability, adhesion, and multifunctionality. In contrast to conventional polymer-modified mortars, polymer concrete (PC) replaces the cement binder with a polymer resin and is valued for its fast strength development, high compressive strength, low permeability, and strong chemical and freeze-thaw resistance, which are advantageous for rapid repairs and corrosion-prone environments.^237,238^ Recent reviews also highlight the growing use of recycled constituents and engineered fillers to improve sustainability while maintaining performance.^238^

A central challenge in structural strengthening is ensuring long-term reliability of epoxy-bonded FRP-to-concrete joints, where adhesive creep and environmental exposure can degrade bond integrity. Nanoparticle-reinforced epoxies can reduce creep, improve crack-bridging, and increase resistance to debonding by strengthening the polymer network and the interphase. Carbon nanomaterials (*e.g.*, graphene oxide) can strengthen epoxy-to-cementitious interfacial bonding through additional polar interactions and improved load transfer, supporting the rationale for nanomodified structural adhesives in FRP retrofits.^239,240^ Consistent with this trend, incorporating functionalized MWCNTs into epoxy has been reported to reduce creep strain by up to 54% relative to neat epoxy in FRP applications.^241^

Nanoclays provide another widely adopted route to improve construction-relevant performance. In epoxy systems, organo-modified clays can increase modulus and strength by promoting more effective stress transfer and restricting polymer chain mobility; improvements of 35–44% (compressive), 22–42% (flexural), and 25–55% (tensile) strength have been reported for clay/epoxy nanocomposites and their polymer concrete formulations.^242^ Beyond static strength, layered silicates are also valuable for fire performance, where the formation of an insulating, tortuous barrier limits heat and mass transfer during burning. Modern multilayer and hybrid clay architectures report synergistic gains in thermal stability and flame resistance by creating highly oriented “nanobrick-wall” barrier structures and increasing char yield.^243^

Protective coatings are an additional high-impact construction use case, particularly for flooring, façades, wood finishes, and infrastructure protection. UV-curable polyurethane acrylate (PUA) coatings can be toughened and hardened with silica nanoparticles, while surface functionalization improves compatibility and reduces defect formation. Inorganic nanoparticles, including nanosilica, are an effective strategy to raise hardness and scratch resistance in UV-curable systems.^244,245^ For example, silane-treated nanosilica in UV-cured urethane acrylate films increased hardness by 40–48% and storage modulus by up to 128%, relative to neat resin 246.

Overall, in construction materials, the performance benefits of hybrid nanoparticle reinforcement are often coupled rather than strictly additive: the same nanophase that improves stiffness may also reshape the curing microstructure, alter interfacial adhesion, and influence transport and fire behavior. This underscores the need for formulation-specific optimization (dispersion route, surface chemistry, and curing schedule) to translate nanoscale effects into robust field performance.^238,243^Table 7 represents polymer nanocomposites with hybrid nanoparticle systems for construction applications.

**Table 7:** Representative polymer nanocomposites (hybrid nanoparticle systems) for construction applications

Polymer system/product form	Hybrid nanoparticles	Typical fabrication route (construction-relevant)	Performance highlights	References
Polymer concrete (PC) repair composite	Fe_3_O_4_ + TiO_2_ nanoparticles	Nanoparticle dispersion into resin *via* high-shear mixing and/or sonication; blend with graded aggregates; cast and cure (ambient or controlled-temperature), then post-cure if required	Improved curing efficiency and compressive strength (repair applications)	247
Epoxy structural adhesive (FRP bonding)	Functionalized MWCNTs	Disperse f-MWCNTs in epoxy (sonication/high-shear), add hardener, apply as a bonding layer for FRP-to-concrete joints, and cure under a service-appropriate schedule	Creep strain reduction up to 54% *vs.* neat epoxy	248
Polymer concrete based on clay/epoxy nanocomposite	Cloisite 30B (organo-clay)	Clay exfoliation/intercalation in epoxy by shear mixing; add curing agent; combine with aggregates for PC; cast and cure	Strength increases: 35–44% compressive, 22–42% flexural, 25–55% tensile	242
Fire-retardant polymer composite	Montmorillonite (MMT) nanoclay	Melt compounding or solution blending, followed by molding or coating; fire-retardant designs often rely on barrier-forming char structures	Reduced flammability *via* barrier/char formation; improved thermal stability	249
Structural polymer composite	MMT nanoclay	Melt compounding (twin-screw extrusion) and molding; often combined with compatibilizers for dispersion	Increased modulus and strength; improved thermal stability	250
UV-curable protective coating	Silane-treated nanosilica	Surface-functionalize nanosilica; disperse into urethane acrylate; apply coating; UV cure	Hardness +40–48%, storage modulus up to +128% *vs.* neat resin	246

### 5.3. Biomedical applications

Polymer nanocomposites containing hybrid nanoparticles have become important in biomedicine because they combine the processability and degradability of polymers with the complementary functions of organic and inorganic nanofillers. By adjusting nanoparticle composition (for example, lipid–polymer, polymer–carbon, or polymer–magnetic hybrids) and engineering the polymer-nanoparticle interface, these materials can be tailored for selective payload encapsulation, controlled release, biointerfacing, and multimodal functionality. As a result, hybrid polymer nanocomposites are being explored across drug delivery, regenerative medicine, imaging, biosensing, and antimicrobial surfaces.

#### 5.3.1. Drug delivery systems

Drug delivery is among the most established biomedical applications of polymer nanocomposites with hybrid nanoparticles. Among these, LPHNPs are widely studied because they integrate a drug-reservoir polymer core with a stabilizing lipid layer and, in many designs, an outer PEG corona to improve colloidal stability and circulation behavior.^251,252^ This architecture supports high loading of hydrophobic agents in the polymeric domain while improving retention and biological performance through the lipid and PEG layers, and it also provides a modular surface for further functionalization (for example, targeting ligands or charge-tuning for nucleic acids).^251–253^

**Fig. 23:**
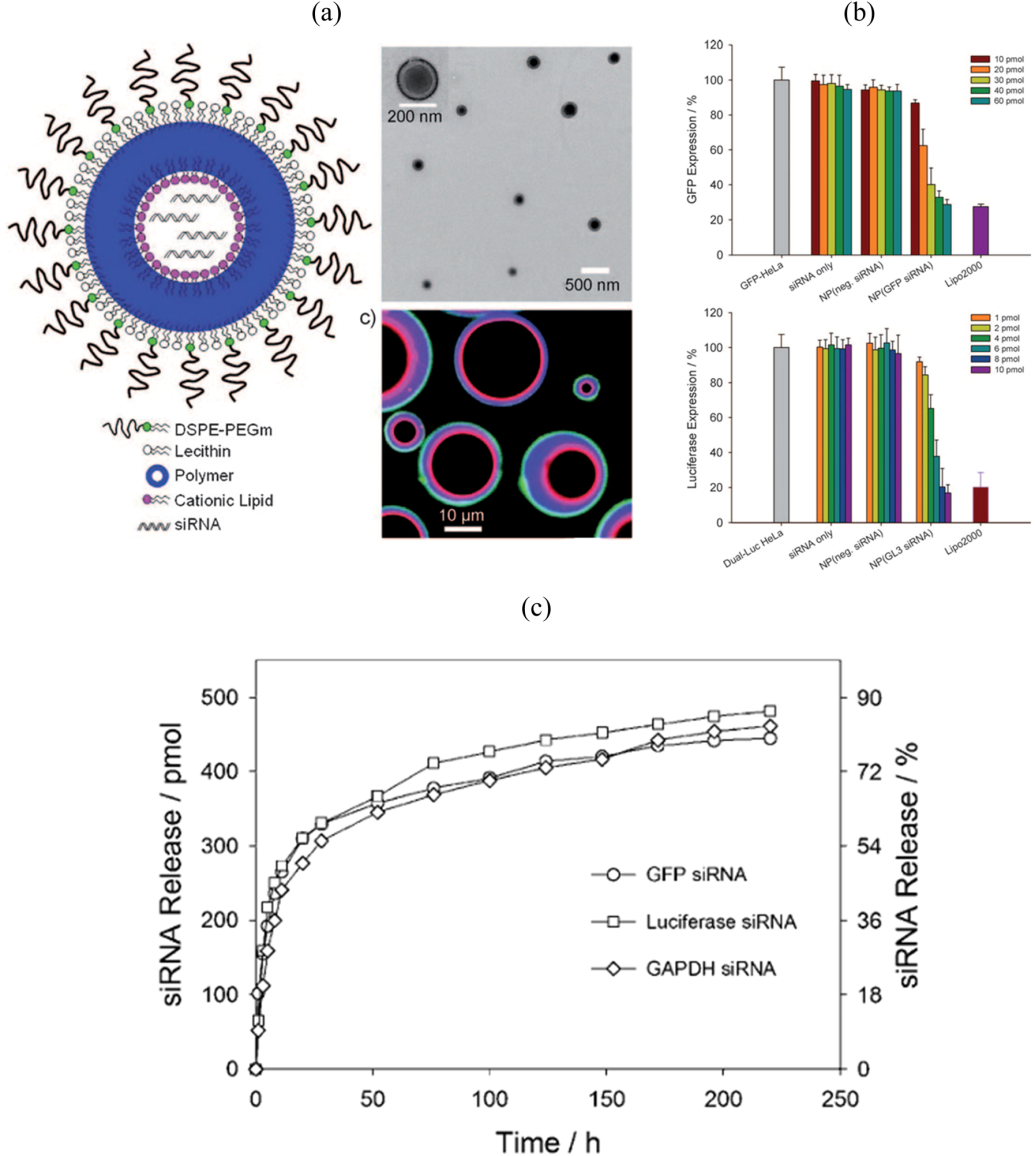
Multilayer lipid–polymer–lipid hybrid nanoparticles: (a) comprising an inner cationic lipid core for siRNA encapsulation, a PLGA polymer layer for controlled release, and an outer PEGylated lipid shell for stability and biocompatibility, with TEM and confocal fluorescence imaging confirming the three-layer architecture; (b) *in vitro* gene-silencing efficiency in GFP-HeLa and Dual-Luc HeLa cells showing dose-dependent silencing with up to ∼72% GFP knockdown at 60 nM siRNA, outperforming naked siRNA and negative controls, and comparable to Lipofectamine 2000 with low cytotoxicity; and (c) sustained *in vitro* release profiles of GFP, GAPDH, and luciferase GL3 siRNAs demonstrating prolonged and consistent release behavior, reproduced from ref. 255 with permission from John Wiley and Sons, copyright 2011 (License No. 6297070173799).

Yu *et al.* developed PEG-lipid-PLGA (polylactic-*co*-glycolic acid) hybrid nanoparticles for the oral delivery of berberine (BBR), using a berberine-soy phosphatidylcholine complex (BBR-SPC) to enhance lipophilicity and polymer affinity. This system achieved a ∼3.4-fold increase in oral bioavailability compared with free BBR, attributed to improved intestinal uptake, efficient mucus penetration *via* the PEG shell, and sustained release from the PLGA core. By synergizing polymer and lipid components, the system effectively addresses solubility and permeability limitations of hydrophobic drugs, establishing a platform for broader therapeutic use.^254^ To advance nucleic acid delivery, Shi *et al.* reported a lipid–polymer–lipid hybrid nanoparticle with a cationic lipid core for siRNA encapsulation, a PLGA middle layer for structural stability, and a neutral lipid-PEG outer shell to reduce immune clearance.^255^ This type of multilayer hybrid remains a useful conceptual template for polymer-based RNA delivery systems, including lipid–polymer formulations investigated for RNA therapeutics.^253^Fig. 23 shows a lipid–polymer–lipid hybrid nanoparticle for siRNA delivery, demonstrating its multilayer architecture, high siRNA loading efficiency (∼80%), and sustained release behavior. The polymer layer ensures structural stability, while the lipid and PEGylated shell enhances colloidal stability and cellular interaction. The hybrid system achieved effective gene silencing (∼72% knockdown) with minimal toxicity *in vitro* and *in vivo*, highlighting its potential for RNA interference-based cancer therapy.^255^

Hybridization strategies are not limited to lipid coatings. Yousaf *et al.* developed chitosan/glyceryl monostearate (GMS) lipid–polymer hybrid nanoparticles for the oral delivery of itraconazole, a poorly soluble antifungal drug. These LPHNPs substantially improved drug dissolution and entrapment efficiency, overcoming the bioavailability challenges of itraconazole. This example illustrates the potential of combining chitosan with lipid excipients to enhance the delivery of therapeutics with poor solubility.^256^ Graphene-based nanocomposites also extend the range of hybrid carriers. Charmi *et al.* reported PEGylated graphene oxide (GO-PEG) loaded with curcumin, achieving ∼4.5% drug loading through π–π stacking interactions. The system exhibited pH-responsive release, with ∼50% and ∼60% release at pH 5.5 and 7.4, respectively, making it suitable for targeting tumor and physiological microenvironments. The PEG matrix enhanced biocompatibility and reduced phagocytic uptake, while GO provided a high surface area for effective drug adsorption.^219^ Moreover, multifunctional nanocomposites can integrate therapeutic and diagnostic roles. Hamzian *et al.* fabricated PLGA (±PEG) nanoparticles incorporating superparamagnetic iron oxide nanoparticles (SPIONs) and gemcitabine *via* a W/O/W double-emulsion method. These hybrid carriers showed controlled gemcitabine release, improved cytotoxicity against MCF-7 breast cancer cells, and potential for radiosensitization and magnetic hyperthermia. The PLGA matrix provided structural integrity and drug stability, while SPIONs added imaging capability and magnetically driven therapeutic enhancement.^257^ Together, these studies show that polymer nanocomposites with hybrid nanoparticles can be engineered to match payload chemistry and route of administration, while also enabling combined therapeutic and diagnostic performance when required.

#### 5.3.2. Tissue engineering

Polymer nanocomposites reinforced with hybrid nanoparticles are increasingly used in tissue engineering because they can simultaneously improve mechanical integrity, bioactivity, and cell–material interactions, which are often difficult to achieve with single-phase scaffolds. In bone regeneration, poly(ε-caprolactone) (PCL) is widely employed due to its biodegradability and processability, but it benefits from hybrid reinforcement to address limitations in osteoinductivity and surface bioactivity. Carbon-based nanofillers such as GO, can enhance stiffness and surface reactivity, while ceramic phases such as hydroxyapatite (HA) provide osteoconductive cues that support mineralization and osteogenic differentiation.^258–260^

A representative strategy is the integration of structural control with multiscale porosity. Shiran *et al.* combined 3D printing and salt leaching to fabricate PCL/HA/GO scaffolds with improved interconnectivity and surface roughness, yielding substantial gains in compressive strength and modulus together with high cell viability and elevated alkaline phosphatase activity, indicating enhanced osteogenic potential.^258^ In a complementary approach, Ahmed and Al-Wafi developed electrospun ε-PCL nanofibers hybridized with Sr/Se-doped hydroxyapatite (Sr/Se-HAP) and GO. The composite showed improved tensile properties and cytocompatibility, with GO contributing to both reinforcement and biological response, supporting its relevance for bone repair and wound-related tissue engineering contexts.^261^

**Fig. 24:**
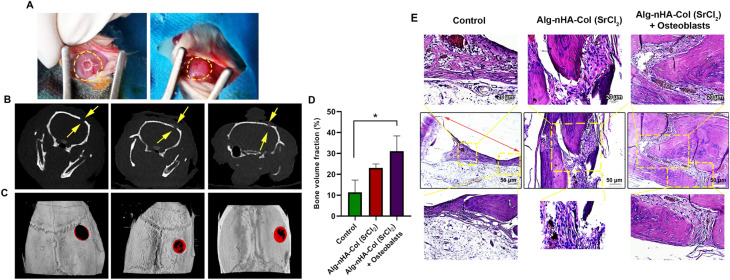
Micro-CT and histological evaluation of calvarial defect repair following implantation of osteoblast-loaded Sr^2+^-cross-linked Alg-nHA-Col microspheres: (A) implantation schematic; (B, C) CBCT and micro-CT images showing *de novo* bone formation (red) after 8 weeks; (D) quantitative micro-CT analysis of bone regeneration (*p* < 0.05, *n* = 4); and (E) H&E-stained sections confirming defect repair, reproduced from ref. 264 under CC BY 4.0 license.

Hybridization with metal–carbon nanofillers can further introduce antimicrobial and osteopromotive functions. Joy *et al.* incorporated GO-Au hybrids into PCL and reported improvements in hydrophilicity and mechanical stability, together with antibacterial activity against both *S. aureus* and *E. coli* and increased osteogenic markers (including ALP activity and mineral deposition), supporting the promise of GO-Au-PCL nanocomposites for infection-resistant bone-regenerative implants.^262^ This concept aligns with broader evidence that gold nanoparticle functionalization of PCL-based 3D scaffolds can promote osteogenic differentiation.^263^ Beyond solid scaffolds, hydrogel-based hybrid systems provide a modular route to osteogenic microenvironments. Hassani *et al.* reported alginate-nano-hydroxyapatite-collagen (Alg-nHA-Col) hydrogels cross-linked with Sr^2+^, showing reduced swelling, improved mechanical behavior, enhanced osteoblast activity, and upregulation of Wnt-associated osteogenic signaling. Importantly, implantation in a critical-sized calvarial defect model demonstrated new bone formation by micro-CT and histology, supporting translational relevance for bone tissue repair, as shown in Fig. 24.^264^

#### 5.3.3. Nanomedicine, antimicrobial, and antiviral applications

Polymer nanocomposites incorporating hybrid nanoparticles have become important platforms for antimicrobial and antiviral technologies because they combine complementary mechanisms within a single material. Typical synergistic actions include ROS generation, disruption of microbial membranes, and controlled release of biocidal ions, while polymer matrices improve processability, stability, and biocompatibility for biomedical applications (*e.g.*, wound dressings and device coatings).^265–267^ Chitosan composites are particularly attractive due to their biocompatibility, biodegradability, and inherent antimicrobial character. Aouadi *et al.* reported a green synthesis route to chitosan-ZnO/PVP (polyvinylpyrrolidone) hybrid nanoparticles that showed strong antibacterial performance, with higher efficacy against Gram-positive *S. aureus*. Notably, the same hybrids exhibited photocatalytic activity, fully degrading methylene blue and toluidine blue, supporting potential use in antimicrobial surfaces and water-hygiene contexts where disinfection and contaminant removal may be coupled. In this system, chitosan contributed to biocompatibility and colloidal stabilization, while PVP improved dispersion and interfacial uniformity.^268^ Motelica *et al.* developed chitosan-based nanocomposite films incorporating ZnO and Ag nanoparticles, further loaded with citronella essential oil (CEO). This multifunctional system demonstrated strong antimicrobial activity against Gram-positive (*S. aureus*), Gram-negative (*E. coli*), and fungal (*Candida albicans*) strains. The synergistic effects of chitosan, metal nanoparticles, and CEO not only enhanced pathogen inhibition but also provided an eco-friendly solution for antimicrobial coatings and food packaging applications, improving shelf life while addressing safety and sustainability concerns. The Ag–ZnO–chitosan ternary nanocomposite also demonstrated significant antibacterial activity against *S. aureus*, *B. subtilis*, *E. coli*, and *P. aeruginosa*, as well as photocatalytic degradation of organic dyes such as methyl orange, supporting applications in both medical device sterilization and environmental remediation.^220^

**Fig. 25:**
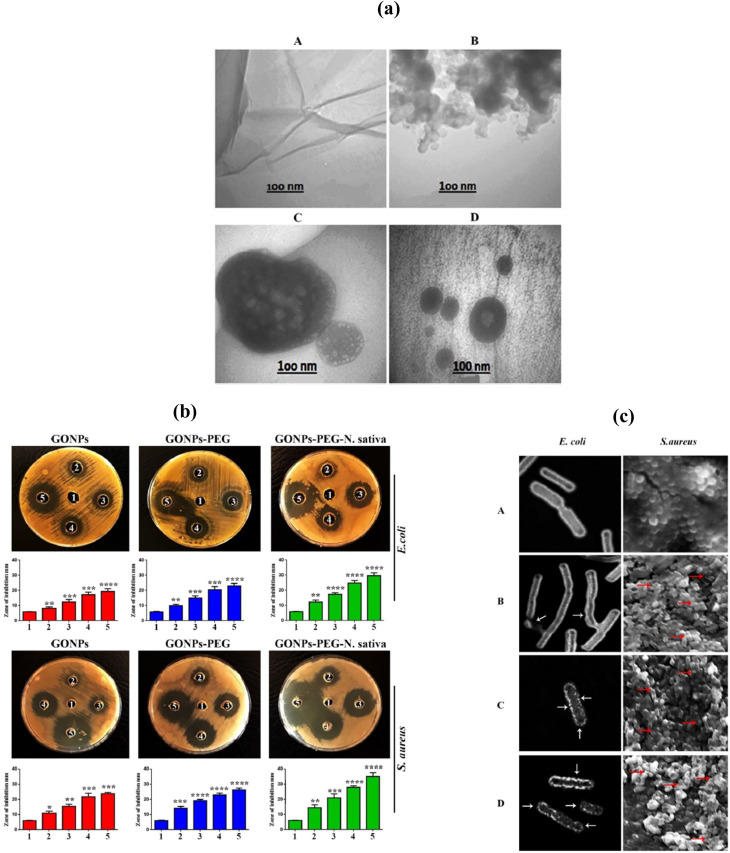
(a) TEM images showing the morphological evolution from single-layered GO nanosheets (<100 nm) to larger, aggregated PEG-functionalized structures (GO-PEG >100 nm) and finally to spherical, tree-like nanocomposites after loading with GO-PEG-*N. sativa*, confirming successful functionalization and drug loading; (b) concentration-dependent antibacterial activity of GO, GO-PEG, and GO-PEG-*N. sativa* against *S. aureus* and *E. coli*, with GO-PEG-*N. sativa* exhibiting the strongest inhibition zones (up to 36 mm for *S. aureus* and 30 mm for *E. coli* at 500 µg mL^−1^); and (c) SEM images revealing the mechanism of action showing untreated bacteria maintaining intact morphology while treated cells show increasing membrane damage, pore formation, aggregation, and structural collapse, reproduced from ref. 271 under CC BY 4.0 license.

PEG is widely used to improve aqueous stability and biocompatibility, which is particularly valuable for graphene-based hybrid nanocomposites. Bao *et al.* showed that PEG-modified graphene oxide-silver nanoparticle composites (GO-AgNPs-PEG) exhibited excellent stability in physiological environments and superior antibacterial activity. Their primary mode of action involved bacterial membrane disruption, highlighting their potential as alternatives to conventional antibiotics for infection-resistant biomedical coatings and wound healing applications.^269^ Related findings across GO-Ag platforms support the view that combining GO interfaces with Ag can enhance antibacterial performance by combining physicochemical injury and ROS-mediated effects.^270^ Similarly, PEG-functionalized GO loaded with *Nigella sativa* extract (GO-PEG-N. sativa) demonstrated strong antibacterial activity against both Gram-positive (*S. aureus*) and Gram-negative (*E. coli*) strains.^271^ The hybrid nanocomposite disrupted bacterial cell wall integrity and induced ROS-associated stress, while maintaining high biocompatibility and enabling the controlled release of plant-derived bioactive compounds. These combined properties support its potential application in infection-responsive wound dressings and antimicrobial surface coatings. Fig. 25 summarizes key structural characterization and antibacterial outcomes for the GO-PEG-N. *sativa* platform.^271^

Beyond chitosan, alginate- and hyaluronic acid-based matrices are widely explored for wound care due to their moisture-handling properties and bio-friendly interfaces. Athamneh *et al.* reported alginate/hyaluronic acid aerogels loaded with ZnO nanoparticles that inhibited *S. aureus* and *E. coli* while maintaining high fluid uptake and sustained ZnO release, properties that align with requirements for infected-wound dressings (exudate management plus durable antimicrobial action).^272^

Hybrid nanocomposites can also target viruses, especially when photoactive or plasmonic components enable ROS-mediated inactivation. Ma *et al.* synthesized Ag/TiO_2_ nanocomposites with improved dispersion and reported dose- and time-dependent antiviral effects against influenza H1N1. Compared with Ag nanoparticles alone, the hybrid provided stronger suppression of infection-associated markers while allowing lower silver loading, thereby reducing cytotoxicity risk in practical use.^273^ Boldogkői *et al.* embedded Ag-modified TiO_2_ photocatalysts into polymer films and demonstrated visible-light-triggered antiviral activity consistent with ROS-driven damage to viral components, indicating the feasibility of durable, self-disinfecting coatings that function under ambient illumination.^274^ These results extend the antimicrobial and environmental applications of such materials into antiviral domains, demonstrating that hybrid nanoparticle-polymer systems can actively inactivate viruses through mechanisms that exploit ROS generation, disrupt viral envelope proteins, and inhibit viral receptor binding. Together, these studies emphasize how polymer-hybrid nanoparticle systems can be engineered to inactivate pathogens *via* ion release, membrane disruption, and light-driven oxidative pathways. Table 8 summarizes representative polymer-based hybrid nanoparticle and scaffold systems spanning drug and gene delivery, tissue engineering, and antimicrobial applications, highlighting how hybridization (*e.g.*, lipid–polymer shells, graphene/metal hybrids, and bioactive ceramic fillers) enables tunable release profiles, improved biocompatibility, enhanced osteogenic performance, and broad-spectrum antibacterial activity.

**Table 8:** Representative polymer-based hybrid nanoparticle systems for drug delivery, tissue engineering, nanomedicine, antibacterial, and antiviral applications

Composition (polymer/hybrid nanoparticles)	Fabrication method	Key functional outcome(s)	Biological target(s)	Representative application	References
PEG-lipid-PLGA hybrid nanoparticles loaded with berberine–phospholipid complex (BBR-SPC)	Solvent evaporation; PLGA core with SPC/DSPE-PEG shell	Encapsulation ∼89%; sustained release; improved oral bioavailability	—	Oral delivery of poorly water-soluble berberine (improved absorption; reduced first-pass loss)	254
PLGA (middle layer) + cationic lipid (inner core) + neutral lipid-PEG (outer shell)	Modified double-emulsion solvent evaporation	High siRNA loading, sustained release, reduced immunogenicity	Cancer cells (gene silencing)	siRNA delivery platform for cancer therapy	255
Chitosan and glyceryl monostearate (GMS) based lipid–polymer hybrid nanoparticles (LPHNPs)	Solvent emulsification-diffusion	Improved dissolution and bioavailability of poorly soluble drugs	—	Oral itraconazole delivery (enhanced antifungal therapy)	256
Polyethylene glycol (PEG)-functionalized GO + curcumin	Modified Hummers method; esterification; π–π stacking loading	pH-responsive release; high loading; improved biocompatibility	Tumor microenvironment (pH 5.5–7.4)	Triggered curcumin delivery for cancer therapy	219
PLGA ± PEG + SPIONs + gemcitabine	W/O/W double emulsification	Controlled release; radiosensitization; hyperthermia; imaging capability	Breast cancer cells (MCF-7)	Theranostic chemotherapy with improved stability and tumor accumulation	257
Polycaprolactone (PCL)/hydroxyapatite (HA)/GO	3D printing (liquid deposition modeling) + salt leaching	Higher osteoconductivity, mechanical reinforcement, and scaffold stability	Bone cells/critical-sized defects	Bone tissue regeneration scaffold	258
Poly (ε-caprolactone) (PCL) + Sr/Se-hydroxyapatite/GO nanofibers	Pulsed laser ablation + electrospinning	Improved roughness and cell attachment; osteoblast proliferation; biocompatibility	Osteoblasts (HFB4)	Wound healing and bone regeneration	261
Polycaprolactone (PCL) + Au-GO nanohybrid	Solvent casting with Au-GO incorporation	Higher hydrophilicity and strength; antibacterial effect; biomineralization (ALP); cell adhesion/viability (HOS)	*Staphylococcus aureus* (Gram-positive); *Escherichia coli* (Gram-negative)	Bone tissue engineering with antibacterial functionality	262
Alginate-nHA-collagen (Alg-nHA-Col)	Ionic cross-linking (Ca^2+^, Ba^2+^, Sr^2+^)	Higher strength; reduced swelling/degradation; osteogenic differentiation *via* Wnt activation	Osteogenic cells	Osteogenic platform for bone regeneration	264
Chitosan-ZnO/PVP	Green biosynthesis (plant-mediated reduction and chitosan encapsulation)	Antibacterial; photocatalytic degradation; radical scavenging	*Bacillus subtilis* (Gram-positive); *Staphylococcus aureus* (Gram-positive); *Pseudomonas aeruginosa* (Gram-negative); *Salmonella typhimurium* (Gram-negative)	Antimicrobial therapy; wound healing; water disinfection; potential antiviral coatings	268
Chitosan + ZnO/Ag NPs + citronella essential oil	Solvent casting	Enhanced antimicrobial activity; UV barrier; reduced water-vapor permeability	*Candida albicans*; *Staphylococcus aureus*; *Escherichia coli*	Active food-packaging coatings (reduced bacterial/fungal growth)	220
Chitosan + Ag/ZnO NPs	Laser ablation	Enhanced antibacterial activity; UV-visible barrier; photocatalysis	*Staphylococcus aureus*, *Bacillus subtilis*, *Escherichia coli*, *Pseudomonas aeruginosa*	Wastewater dye degradation; antimicrobial coatings for medical/sanitary uses	275
Polyethylene glycol (PEG)-modified GO-AgNPs	Green synthesis using gallic acid (reductant/stabilizer)	Improved dispersion stability; broad-spectrum antibacterial activity; biocompatibility	Drug-resistant bacteria (broad-spectrum)	Antibacterial coatings, wound dressings, and device sterilization	269
PEG-functionalized GO + Nigella sativa extract	Hummers method; esterification; π–π stacking loading	Enhanced antibacterial activity; biocompatibility; delivery efficiency	*Staphylococcus aureus*; *Escherichia coli*	Smart antibacterial drug-delivery system for wound healing	271
Alginate/hyaluronic acid + ZnO NPs	Supercritical gel drying	Antibacterial activity; high fluid uptake; sustained ZnO release	*Staphylococcus aureus*; *Escherichia coli*	Antibacterial wound dressing with exudate management	272

### 5.4. Environmental applications

Hybrid nanoparticle-reinforced polymer nanocomposites have emerged as versatile platforms for environmental remediation because they can integrate multiple functions into a single material, including adsorption, catalytic degradation, antimicrobial activity, and improved stability under operating conditions. Such multifunctionality is particularly valuable in complex waste streams where contaminant mixtures, variable pH, and competing ions limit the performance of single-function sorbents or catalysts.^276^ In practice, environmental applications of these hybrids have expanded across (i) water and wastewater treatment (adsorption, disinfection, and membrane separations), (ii) air purification (photocatalytic oxidation of volatile organic compounds), and (iii) soil remediation (immobilization and stabilization of heavy metals and persistent pollutants).

In wastewater treatment, hybrid nanocomposites are widely explored as adsorbents for dyes and toxic metal ions. Their effectiveness typically arises from coupling fast adsorption kinetics and high interfacial area (provided by the nanofillers) with the mechanical integrity and tunable surface chemistry of the polymer supports, enabling regeneration and reuse.^277^ For dye removal, Esvandi *et al.* developed a clay/starch/MnFe_2_O_4_ magnetic nanocomposite capable of removing both anionic (sunset yellow) and cationic (Nile blue) dye.^278^ In this ternary design, clay provided a porous scaffold, starch contributed polar functional groups that aided dispersion and adsorption, and MnFe_2_O_4_ provided additional active sites and magnetic separability. The reported performance was attributed to synergistic interactions, including electrostatic attraction and hydrogen bonding, while magnetic recovery simplified post-treatment separation.

**Fig. 26:**
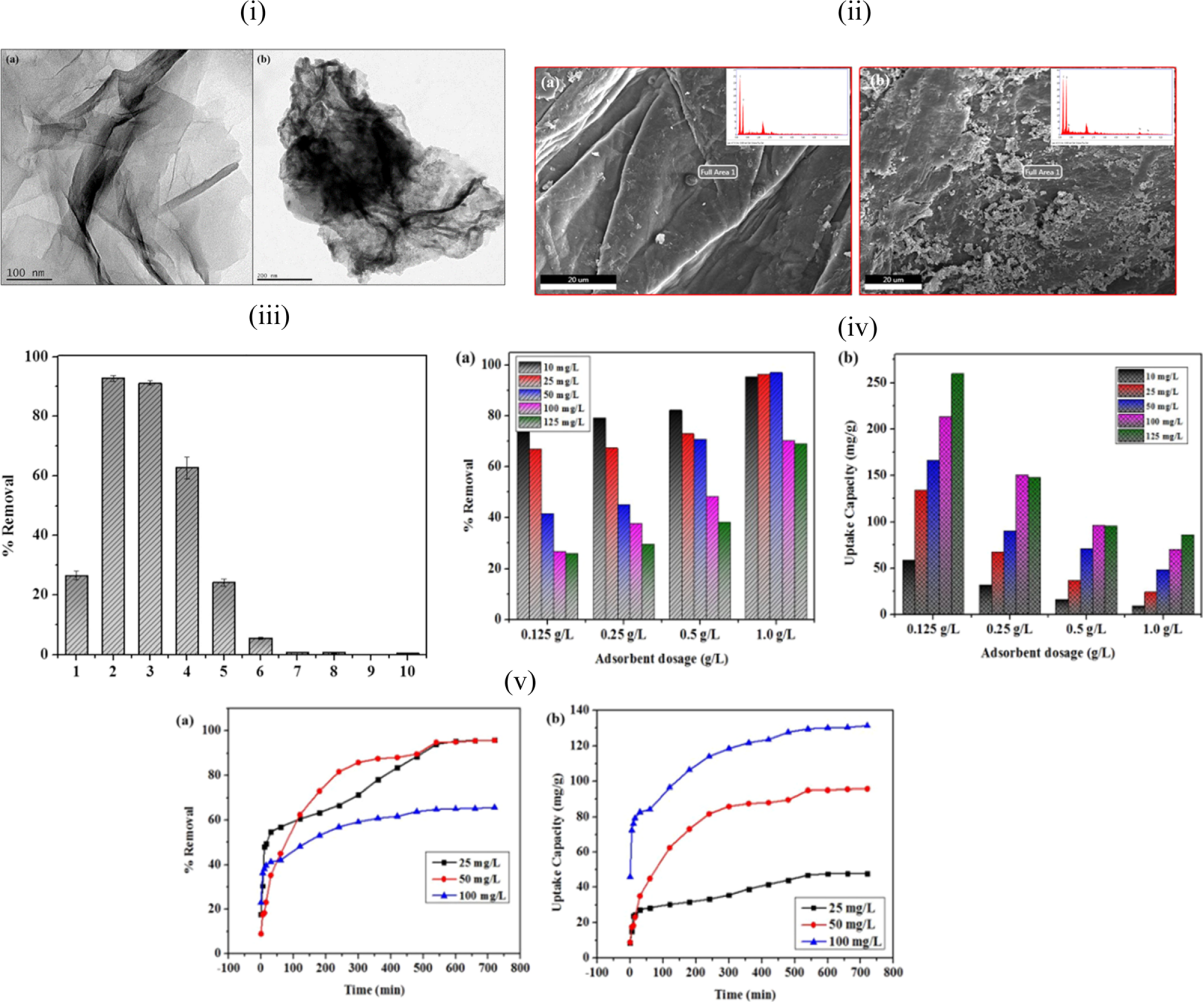
(i and ii) TEM and SEM images of GO and GCF showing the transformation from smooth, layered GO sheets to a rough, aggregated hybrid structure after incorporation of chitosan and Fe_3_O_4_ nanoparticles; (iii) effect of solution pH on Cr(vi) adsorption, with maximum removal (∼96%) at pH 2 due to enhanced electrostatic attraction between protonated GCF surfaces and anionic Cr(vi) species; and (iv and v) influence of adsorbent dosage, contact time, and initial Cr(vi) concentration on adsorption efficiency and uptake capacity, reproduced from ref. 279 with permission from Elsevier, copyright 2018 (License No. 6297071144712 and 6297071264556).

Hybridization is similarly beneficial for the remediation of toxic metals. Samuel *et al.* developed a GO/chitosan/ferrite (GCF) nanocomposite and reported a high Cr(vi) adsorption capacity of 270.27 mg g^−1^ at pH 2, outperforming many conventional sorbents. As illustrated in Fig. 26, incorporation of chitosan and ferrite transforms smooth GO sheets into a rough, aggregated hybrid structure and results in strongly pH-dependent adsorption, with maximum Cr(vi) removal (∼96%) under acidic conditions. The adsorption efficiency and capacity are further influenced by adsorbent dosage, contact time, and initial Cr(vi) concentration. In this system, GO provides a high-surface-area scaffold, chitosan supplies amine and hydroxyl binding sites, and ferrite nanoparticles enable magnetic recovery while contributing additional active sites. The adsorption followed the Langmuir isotherm and pseudo-second-order kinetic models, and the material retained over 80% of its removal efficiency after ten reuse cycles, reaching equilibrium at approximately 600 min. Overall, this study highlights how hybrid nanocomposite design enhances adsorption performance, recyclability, and practical applicability.^279^

Hybrid nanocomposites can also provide antimicrobial activity through contact-active surfaces and controlled release of biocidal species. Polypyrrole (PPy)/CNT/Ag nanoparticle nanocomposites have demonstrated near-complete bacterial removal in water disinfection studies.^280^ In this system, CNTs enhanced surface area and promoted bacterial contact, Ag nanoparticles provided bactericidal activity (including *via* Ag^+^ release), and PPy contributed structural cohesion and surface charge that aided microbial capture and nanoparticle dispersion. More broadly, Ag nanoparticle-based approaches are a promising route toward efficient water disinfection when release, stability, and safety are appropriately controlled.^280,281^

Hybrid polymer-inorganic systems are increasingly proposed for indoor air purification, where photocatalytic oxidation (PCO) is a leading approach for degrading volatile organic compounds (VOCs). TiO_2_-based PCO has been reviewed as a potentially cost-effective strategy for VOC removal, while highlighting key design constraints, including light utilization, adsorption capacity, and by-product formation.^282^ In this context, biodegradable PLA/TiO_2_ composite films have demonstrated photocatalytic oxidation performance suitable for indoor purification, aligning pollutant abatement with polymer sustainability.^283^ Complementary strategies include immobilizing TiO_2_ in functional coatings; perfluorinated ionomer/TiO_2_ multilayer coatings have been evaluated for VOC adsorption and gas-phase photocatalytic degradation under irradiation, illustrating how polymer chemistry can be engineered to improve pollutant uptake while stabilizing photocatalyst particles.^284^ For bioaerosol control, hybrid photocatalytic-antimicrobial designs offer additional value. Ag/TiO_2_/PLA biofilms have been reported to combine photocatalytic activity with antimicrobial effects, achieving significant *Staphylococcus aureus* removal in air purification configurations.^285^

Hybrid nanocomposites also contribute to soil remediation, particularly for immobilizing heavy metals and reducing bioavailability. Nanoparticle-modified biochars, such as MnO_2_- or ZnO-decorated biochar, have been shown to reduce the mobility and bioavailability of metals (including Pb and Cd) in multi-metal contaminated soils under cultivation conditions.^286^ Biochar-based composites containing layered double hydroxides (LDHs) represent another active class, combining biochar porosity and surface functionality with LDH ion exchange and precipitation capacity.^287^ Several studies^288–290^ also emphasized their strong potential for metal immobilization and broader improvements in soil physicochemical properties.

Overall, hybrid polymer nanocomposites provide versatile, multifunctional platforms for dye adsorption, metal ion capture, disinfection, photocatalytic abatement of airborne pollutants, and soil stabilization. For translation to field-scale operation, future work may emphasize nanoparticle retention and aging stability (to minimize leaching), regeneration efficiency, performance under complex real matrices (natural organic matter, competing ions), and life-cycle considerations, especially for photocatalytic systems where by-products can form during VOC oxidation.^282,291^

### 5.5. Energy storage and conversion applications

Polymer nanocomposites reinforced with hybrid nanoparticles are increasingly recognized as highly effective materials for energy storage and conversion. By exploiting nanoscale synergistic interactions, these systems overcome the limitations of traditional polymer-based devices. Earlier strategies to enhance the dielectric energy density of polymers relied on micro-sized fillers or thick dielectric layers, which provided only limited improvements. Modern approaches instead focus on hybrid nanocomposites, where multiple nanoscale components, such as grafted polymers, crosslinked domains, nanofillers, or ultrathin coatings, are combined to optimize intrinsic properties and interfacial effects.^292^ For example, a ternary ferroelectric polymer composite embedding boron nitride nanosheets (BNNSs) and barium titanate (BT) nanoparticles into P(VDF-CTFE) achieved an energy density of 21.2 J cm^−3^, nearly five times higher than biaxially oriented polypropylene (BOPP). In this system, BNNSs improved breakdown strength and mechanical robustness, while BT nanoparticles increased the dielectric constant. The hybrid design also prevented nanoparticle aggregation, demonstrating how carefully engineered multi-nanoparticle strategies can resolve the conventional trade-off between dielectric constant and breakdown strength.^292^

**Fig. 27:**
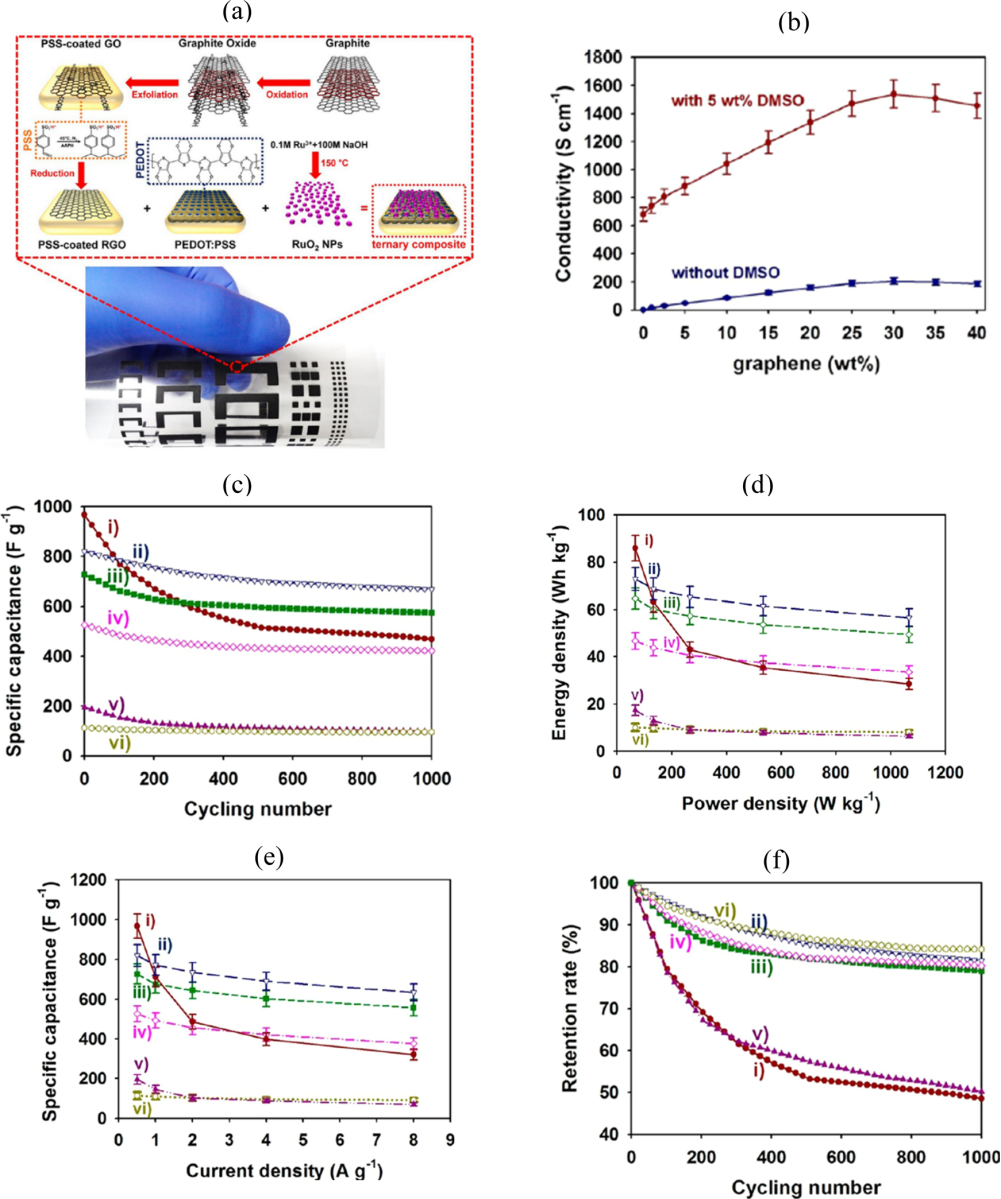
(a) RuO_2_/PEDOT: PSS/graphene screen-printed electrode fabrication procedure and digital camera photograph of screen-printed electrode. (b) Electrical conductivity of PEDOT: PSS/graphene composites *versus* graphene content, showing a maximum of ∼1540 S cm^−1^ at 30 wt% graphene. (c) Specific capacitance *versus* current density for different electrodes, with RuO_2_/PEDOT-G30 delivering 820 F g^−1^ at 0.5 A g^−1^. (d) Ragone plot highlighting the balanced energy–power performance of RuO_2_/PEDOT-G30 (73 Wh kg^−1^ at 67 W kg^−1^). (e and f) Cycling performance over 1000 cycles in terms of specific capacitance and retention rate, respectively, where RuO_2_/PEDOT-G30 retains ∼81.5% of its initial capacitance. Panels (a)–(c), and (f) reproduced from ref. 293 with permission from American Chemical Society, copyright 2015 (License No. 6297080323190). Panels (d) and (e) reproduced from ref. 293 with permission from American Chemical Society, copyright 2015 (License No. 6297080500310).

Conjugated polymers, though widely used in supercapacitors, often suffer from cycling stability issues and limited ion mobility. Hybridization with inorganic or carbon-based nanomaterials addresses these shortcomings. Fig. 27 shows ternary composites such as PEDOT: PSS/graphene/RuO_2_ that achieved a conductivity of 1570 S cm^−1^, a specific capacitance of 820 F g^−1^, and an energy density of 73 Wh kg^−1^, with 81.5% retention after 1000 cycles.^293^ In this system, graphene formed conductive networks, RuO_2_ contributed pseudocapacitance, and PEDOT: PSS ensured uniform dispersion. Similarly, PANI/TiO_2_/GO composites combined GO's conductive framework, TiO_2_'s thermal stability, and PANI's pseudocapacitance, achieving 1020 F g^−1^ at 2 mV s^−1^ with excellent cycling stability.^294^ These examples illustrate how hybrid nanocomposites maximize interfacial interactions, reduce charge-transfer resistance, and stabilize the polymer matrix during cycling.

Hybrid nanocomposites are also attractive in photovoltaics due to their low fabrication cost, tunable optical properties, and structural versatility.^295^ PPy/Cu/GO films exhibited improved electrical conductivity and visible-light absorption, with GO tuning the bandgap and morphology, and Cu enhancing charge transport.^296^ Similarly, P3HT combined with functionalized graphene (SPFGraphene) and f-MWCNTs formed a bulk heterojunction that balanced 1D and 2D charge pathways, improving exciton dissociation and power conversion efficiency (1.05%).^297^ Other innovations include P3HT (poly(3-hexylthiophene)) and PCBM (phenyl-C61-butyric acid methyl ester) composites enhanced with graphene quantum dots, which achieved 5.24% efficiency through simultaneous exciton separation and light harvesting,^298^ and plasmonic Au@PEG-GO hybrids, which improved light absorption and exciton dissociation across multiple layers.^299^ Overall, these examples highlight how controlled nanoparticle incorporation, optimized blending, and stable hybrid structures enhance charge mobility, reduce recombination, and improve long-term device stability.^295,300^

Beyond storage and photovoltaics, polymer nanocomposites with hybrid nanoparticles also excel in photocatalytic hydrogen generation. Nb_6_/PPy-RGO composites combined polyoxoniobate (Nb_6_) as a light absorber, PPy as a conductive bridge, and RGO as a scaffold, achieving hydrogen evolution rates 43 times higher than pure Nb_6_, without the need for noble metals.^301^ Likewise, PTh-rGO-TiO_2_ composites enhanced charge separation, visible-light absorption, and dye degradation efficiency, demonstrating the complementary roles of different nanoparticles in creating 3D interconnected architectures with abundant active sites.^301^ Overall, these studies confirm that hybrid nanoparticle–polymer nanocomposites can be rationally designed to deliver multifunctional performance in energy storage, conversion, and generation, with each component contributing complementary properties to enhance efficiency, durability, and scalability.

### 5.6. Electronics and sensor technology applications

Advanced sensor technologies require multifunctional properties, including miniaturization, rapid response, high sensitivity, selectivity, reliability, low cost, and long-term stability. Many of these requirements are inherently linked to high surface area and interfacial interactions, characteristics that polymer nanocomposites reinforced with hybrid nanoparticles can provide.^276^ Incorporating multiple nanomaterials into polymer matrices combines distinct physicochemical and structural properties, enabling multifunctional performance for biosensing, environmental monitoring, gas sensing, and EMI shielding.^276^

**Fig. 28:**
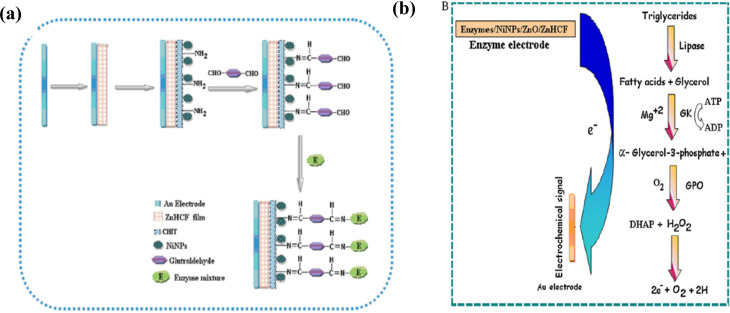
(a) Stepwise assembly of the triglyceride biosensor *via* covalent immobilization of lipase, glycerol kinase (GK), and glycerol-3-phosphate oxidase (GPO) on NiONPs-chitosan using glutaraldehyde, followed by integration onto an electrodeposited ZnO–ZnHCF film. (b) Schematic of the coupled enzymatic and electrochemical reactions underlying the triglyceride biosensor, where enzymatic conversion generates electroactive species that produce the analytical signal, reproduced from ref. 302 with permission from Elsevier, copyright 2013 (License No. 6297091037985).

Hybrid polymer nanocomposites have been widely employed in electrochemical biosensors owing to their ability to enhance conductivity, enzyme immobilization, and catalytic efficiency. For instance, a high-performance amperometric triglyceride biosensor (Fig. 28) was fabricated using a ternary composite of nickel oxide nanoparticles (NiONPs), chitosan, and a ZnO–ZnHCF (zinc hexacyanoferrate) hybrid film.^302^ NiONPs provided a high surface area for enzyme immobilization and efficient electron transfer, while chitosan stabilized the enzyme and prevented leakage, and ZnO–ZnHCF enhanced conductivity and corrosion resistance. The resulting biosensor demonstrated rapid response (4 s), a wide linear range (50–700 mg dL^−1^), a low detection limit (10 mg dL^−1^), high specificity, and long-term stability (50% activity retained after 180 days). Similarly, a cholesterol biosensor developed with AuNPs and f-MWCNTs embedded in a PPy network achieved high sensitivity (10.12 µA mM^−1^ cm^−2^), low detection limit (0.1 × 10^−3^ M), and robust stability, retaining 76% activity after 7 days.^303^ These examples highlight how hybrid fillers synergistically improve conductivity, catalytic activity, and stability.

Polymer nanocomposites have also demonstrated excellent performance in heavy metal ion sensing. A disposable sensor using AuNPs on PANI-MWCNTs achieved ultra-low detection limits for Zn^2+^, Pb^2+^, and Cu^2+^, with recoveries exceeding 93% in real water samples.^304^ Similarly, a polythiophene (PTH)-based hybrid nanocomposite incorporating carboxylated MWCNTs and RGO enabled highly selective detection of Hg^2+^, with a detection limit of 0.009 µM.^305^ In this system, MWCNTs offered abundant adsorption sites, RGO enhanced electron transport, and PTH ensured stability and efficient electron transfer. A P2MA-based composite reinforced with cerium tungstate [Ce_2_(WO_4_)_3_] and CNTs formed a mulberry-like structure with ion-exchange capacity, high conductivity, and thermal stability, achieving ultra-high sensitivity (∼5.138 µA µM^−1^ cm^−2^), low detection limit (0.11 nM), and rapid response (∼10 s) for Cd^2+^ detection.^306^ Furthermore, a polyaniline (PANI), poly(*o*-toluidine) (PoT) copolymer (PANI-*co*-PoT)/GO nanocomposite reinforced with AuNPs enabled sensitive Cr(vi) detection, offering a porous morphology, excellent selectivity, and a detection limit of 0.0215 µM.^307^

Hybrid polymer nanocomposites are also effective for gas and humidity sensing. A chitosan/ZnO/SWCNT ternary nanocomposite functioned as a high-performance humidity sensor, exhibiting superior sensitivity across 11–97% RH, fast response/recovery, low interference, and long-term stability.^308^ Ag-doped polyindole/hydroxyethyl cellulose composites have also been reported as next-generation humidity sensors, showing fast response and improved stability.^309^ Chitosan provided hydrophilic adsorption sites, ZnO improved free volume and diffusion pathways, and SWCNTs formed a conductive network. The composite demonstrated fast response/recovery, low interference, and long-term stability. Hierarchical 3D nanocomposites, such as ZnFe_2_O_4_/PANI/RGO for *p*-nitrophenol detection^310^ and RGO/Ag/poly-l-lysine (PLL) for bisphenol A detection,^311^ have demonstrated high sensitivity and selectivity. In these systems, PANI or PLL contributed redox activity, RGO enhanced conductivity, and metal nanoparticles provided catalytic activity, demonstrating the versatility of hybrid designs for pollutant detection.

By incorporating conductive nanofillers such as metal, magnetic, or carbon-based nanoparticles, polymer nanocomposites can achieve high shielding performance while remaining lightweight and versatile.^276^ For instance, in one study,^312^ a lightweight and flexible EMI shielding material was developed by electrospinning carboxymethyl cellulose (CMC) nanofibers that were embedded with multiple metal nanoparticles (MNPs). The incorporation of different nanoparticles provided complementary benefits: conductive MNPs, such as Cu and Zn, significantly enhanced electrical conductivity and reflection-based shielding, while magnetic MNPs, such as Fe and Co, improved absorption *via* magnetic permeability and dielectric losses. The combination of these nanoparticles created synergistic effects, achieving high shielding effectiveness that could be tuned by varying MNP type and content. The nanocomposite maintained mechanical strength, avoided nanoparticle aggregation, and remained eco-friendly and processable.^312^

Overall, polymer nanocomposites reinforced with hybrid nanoparticles enhance sensor performance by improving conductivity, selectivity, stability, and multifunctionality. Their adaptability across biosensing, heavy metal detection, gas sensing, pollutant monitoring, and EMI shielding positions them as next-generation materials for advanced electronics and environmental monitoring.

### 5.7. Coatings, adhesives, and packaging applications

Polymer nanocomposites are increasingly adopted in food packaging, coatings, and adhesives because nanoscale reinforcement can concurrently tune mechanical integrity, transport resistance, and bioactivity. Compared with single-filler systems, hybrid nanoparticles (two or more nanofillers combined as blends, heterostructures, or core–shells) can deliver co-optimized performance by (i) expanding the range of functional mechanisms (for example, barrier plus antimicrobial), and (ii) improving dispersion and interfacial coupling to translate nanoscale effects into bulk properties.^313^ Hybrid nanofillers are particularly valuable in active food packaging, where high tensile strength, low water vapor permeability, UV shielding, and antimicrobial activity are required simultaneously. A carrageenan-based film containing AgNPs and organically modified clay (Cloisite® 30B) demonstrated improved strength, reduced water vapor transmission, UV blocking, and broad antimicrobial activity, with the clay contributing transport resistance and AgNPs providing antibacterial function.^314^

**Fig. 29:**
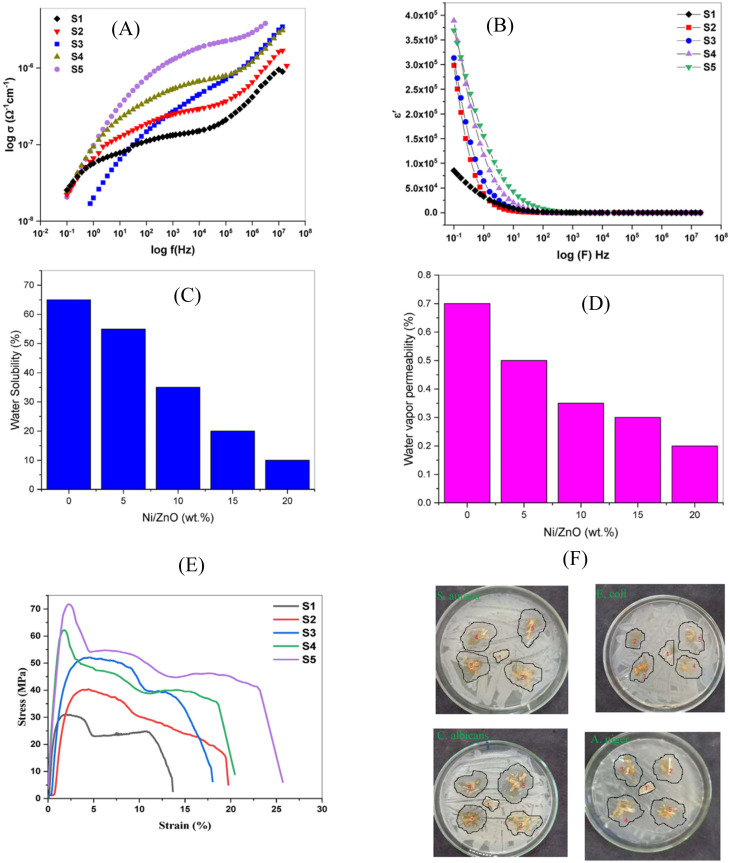
Development and characterization of NaAlg/PEO films doped with Ni/ZnO nanohybrids for active food packaging. (A) AC conductivity and (B) dielectric constant increase with Ni/ZnO content and vary with frequency, indicating enhanced charge transport and dipolar polarization. (C) Water solubility and (D) water vapor permeability decrease with Ni/ZnO loading, thereby improving moisture resistance. (E) Stress–strain behavior confirms mechanical reinforcement, with tensile strength increasing from 30.18 MPa to 72.34 MPa at 20 wt% Ni/ZnO. (F) Antimicrobial inhibition zones against *E. coli*, *S. aureus*, *A. niger*, and *C. albicans* increase with nanofiller concentration, reproduced from ref. 315 under Creative Commons Attribution (CC BY) license.

Similarly, sodium alginate (NaAlg)/poly(ethylene oxide) (PEO) films doped with Ni/ZnO nanohybrids showed a multi-property upgrade that aligns well with packaging needs: lowered water solubility and water vapor permeability (improved moisture resistance), marked mechanical reinforcement, and clear antimicrobial inhibition against representative bacteria and fungi.^315^Fig. 29 shows the role of Ni/ZnO in improving electrical, mechanical, antibacterial, and barrier performance, making the nanocomposite a promising candidate for biodegradable and functional food packaging materials. The same system also exhibited frequency-dependent electrical and dielectric enhancements, which can be considered a secondary advantage for smart or functional packaging concepts (*e.g.*, electroactive sensing layers), provided the manuscript explicitly links the electrical response to the intended packaging function.^316^

Beyond blended fillers, core–shell nanostructures help couple stability with photoactivity. For example, degradable sodium alginate films reinforced with Au–TiO_2_ core–shell nanoparticles showed strong antibacterial performance under visible light (Au improves visible response; TiO_2_ supports photocatalytic ROS generation) while maintaining biodegradability.^317^ Finally, biopolymer films containing bimetallic Ag–Cu nanoparticles have been reported to outperform single-metal counterparts in thermomechanical and antimicrobial performance, consistent with the broader literature, which attributes bimetallic superiority to multimodal antimicrobial mechanisms and metal–metal synergy.^318^

Hybrid nanoparticle strategies are also central to antimicrobial and anticorrosion coatings, where long-term adhesion and transport blocking are decisive. A PLA film containing AgNPs and TiO_2_ achieved stronger antimicrobial action than single-filler analogs, consistent with the complementary roles of Ag ion release and TiO_2_-mediated ROS pathways, enabling efficacy at lower total nanoparticle loading and supporting environmental impact minimization through material reduction.^319^ For corrosion protection, dual-nanoparticle systems improve barrier performance primarily by creating tortuous diffusion pathways and stabilizing the polymer–substrate interface. Epoxy matrices reinforced with GO and nanoclay showed improved impedance retention and adhesion upon exposure, reflecting the synergy between high-aspect-ratio GO sheets and layered silicates in restricting the ingress of corrosive species.^320^ More complex hybrids, such as GO-Fe_3_O_4_ (with surface functionalization to improve dispersion and bonding), can further increase hydrophobicity and micro-hardness while strengthening barrier and passivation effects.^321^ GO-based composite coatings are most effective when dispersion, interface chemistry, and defect control are engineered together rather than relying solely on graphene content.^322^

In adhesives, hybrid nanoparticles can address the classic trade-off between conductivity (or stiffness) and toughness. A flexible, electrically conductive adhesive based on thermoplastic polyurethane (TPU) with Ag flakes and polyaniline@cellulose nanowhiskers formed a percolated 3D network, in which the high-aspect-ratio conductive bridges reduced resistivity by orders of magnitude compared with Ag-only systems, while preserving flexibility for wearable or bendable electronics.^323^ For structural bonding, hybrid toughening is particularly attractive because rigid nanoparticles can raise modulus and strength but often embrittle epoxies unless paired with an energy-dissipating phase. Epoxy adhesives containing core–shell rubber (CSR) particles plus ZnO nanoparticles showed large gains in fracture toughness (218% increase) alongside improved lap shear and thermal stability, indicating complementary mechanisms (crack deflection/voiding from CSR and reinforcement/constraint from ZnO).^324^ CSR-based toughening is most effective when combined with an additional rigid phase, which preserves stiffness and the glass transition temperature while increasing damage tolerance.^325^

Because coatings and food packaging operate at direct environmental/food-contact interfaces, many journals increasingly expect risk- and sustainability-aware material selection, particularly minimizing nanofiller loading, designing for controlled release/migration, and performing early-stage life-cycle screening.^319^ Life cycle assessment has been used to compare nanomaterial-based active agents for packaging and guide selection trade-offs, while Safe-and-Sustainable-by-Design (SSbD) frameworks provide a structured way to identify environmental and (eco)toxicity hotspots early in development.^326^

### 5.8. Flexible and wearable electronics applications

Flexible and wearable electronics require mechanically compliant conductors and sensors that maintain electrical performance under bending, twisting, and large tensile strain while providing stable, low-impedance contact with soft, dynamic biological surfaces. Polymer nanocomposites incorporating hybrid nanoparticles address these constraints by combining complementary filler geometries and functions (for example, 0D–1D, 1D–2D, or 0D–1D–2D combinations) to form hierarchical percolation networks within elastomers or other soft matrices. Compared with single-filler systems, hybrid networks can reduce percolation thresholds, improve crack tolerance and cyclic durability, and stabilize inter-filler contacts during deformation, enabling robust interconnects, strain sensors, and epidermal biointerfaces for wearable platforms.^327–330^

A representative scalable route is printed soft electronics, where aerosol jet printing of polyimide and silver nanowires produced miniaturized, nanostructured strain sensors with tunable resistance and gauge factor. The multilayer, compliant design enabled strong adhesion and smooth lamination to diverse substrates without auxiliary fixtures; a printed two-axis gauge tracked strain on curved hardware and remained reusable over repeated mounting cycles. A skin-compatible version accurately monitored human motion and vital signals (pulse and respiration), highlighting the suitability of printed hybrid nanocomposites for continuous structural and physiological monitoring.^331^ Hybrid metallic architectures further improve sensitivity and stability by engineering junction formation. For example, Ag-flake/Ag-nanocrystal hybrids embedded in PDMS used intense pulsed light to rapidly sinter nanocrystals without damaging the elastomer, thereby enhancing inter-flake electrical coupling and strain sensitivity. The resulting sensors sustained large deformation (up to 80% strain), exhibited a gauge factor of 7.1, and demonstrated stable cycling over 10 000 stretch-release cycles (50% strain), and were integrated into a smart glove and sports band for motion and force tracking.^332^

Hybridization is also effective for mitigating filler aggregation and maintaining conductive pathways at high strain. A ternary system of PS (polystyrene) microspheres, RGO, and Ag nanowires formed *via* electrostatic assembly achieved high conductivity (136.25 S m^−1^), a low percolation threshold (0.159 vol%), and a maximum conductivity of 1230 S m^−1^ at 3.226 vol% loading. These properties enabled a flexible electronic skin that combined stretchability, sensitivity, and cyclic durability for prosthetic finger-motion monitoring.^333^ Likewise, wet-spun SBS (styrene-butadiene-styrene) fibers containing embedded Ag nanowires and *in situ*-formed Ag nanoparticles maintained conductivity during stretching because the nanowires acted as conductive bridges between nanoparticle domains; these fibers provided reliable strain sensing across a broad strain window and enabled sign-language detection when integrated into a smart glove.^334^ Biointerfacing performance can be further improved by coupling hybrid nanocomposites with bioinspired mechanics: a gecko-inspired conductive dry adhesive based on 1D–2D hybrid carbon fillers (CNTs with graphene) achieved low volume resistance through synergistic percolation, replicated high-aspect-ratio microstructures over large areas, adhered strongly to skin, and supported reusable, metal-free electrophysiological monitoring under daily activity.^335^ Moreover, hybrid semiconducting-carbon architectures extend wearable sensing beyond pure piezoresistive metals. A MoS_2_/graphene porous network (GPN)/Ecoflex composite, where conformal MoS_2_ sheets and nanoflakes were grown on a graphene foam scaffold, produced large resistance modulation under strain and pressure with durability over thousands of cycles, enabling on-body drowsiness detection and a 3 × 3 tactile array with spatially resolved touch sensing.^336^

Literature also emphasizes that many wearable systems, especially electrophysiology and long-term epidermal interfaces, are limited by impedance stability (not only DC resistance) under deformation and sweat exposure; material choices (metals, conducting polymers, hydrogels, liquid metals) and deformable architectures (for example, open-mesh and kirigami motifs) are increasingly treated as co-design variables for low-noise signal acquisition and comfort.^337,338^ In parallel, emerging soft-conductor families such as liquid-metal composites and MXene-based composites offer additional pathways for hybrid nanocomposite design, since they can combine high conductivity with intrinsic deformability (liquid metals) or high conductivity with solution processability and surface-functional tunability (MXenes), supporting multifunctional wearable sensing and robust interconnects.^339,340^

### 5.9. Energy harvesting devices

Polymer nanocomposites containing hybrid nanoparticles have been widely used in energy-harvesting devices. The incorporation of multiple distinct materials into a single nanocomposite has enabled the exploitation of combined properties, enhanced functionalities, and performance advantages that could not be achieved with a single material or a simple composite. In one study, synthesis and dual application of a ZnO/functionalized multi-walled carbon nanotube (f-MWCNT) composite for supercapacitive energy storage and piezoelectric energy harvesting were investigated.^341^ For piezoelectric energy generation, the composite was incorporated into a polyvinylidene fluoride (PVDF) matrix to form a flexible polymer nanocomposite film. The inclusion of 1.0 wt% ZnO/f-MWCNT significantly enhanced the electroactive β-phase fraction of PVDF (β-phase fraction ≈ 1.71) without electrical poling, which is crucial for piezoelectric performance. The resulting piezoelectric nanogenerator generated a high open-circuit voltage of ∼80 V and a remarkable power density of 72 kW m^−3^ under periodic mechanical stress, demonstrating efficient energy conversion from finger tapping. The use of a polymer nanocomposite. PVDF (polyvinylidene fluoride)/ZnO/f-MWCNT) was highly beneficial, as f-MWCNTs improved electrical conductivity and provided mechanical reinforcement; ZnO nanorods enhanced pseudocapacitance and acted as nucleation sites for β-phase PVDF crystallization; and PVDF served as a flexible, piezoelectric matrix that could be easily processed into films for wearable or portable devices. Another study also explored polyvinylidene fluoride/zinc oxide/carbon nanotube (PVDF/ZnO/CNT) nanocomposites for piezoelectric energy harvesting under cyclic uniaxial tensile deformation.^342^ The inclusion of ZnO and CNT nanoparticles significantly enhanced the electroactive β-phase content of PVDF, which is crucial for piezoelectric performance. The composite with 15% ZnO exhibited the highest β-phase fraction (62%) and crystallinity (51.16%).

Under cyclic tensile loading at 2 Hz and 10 N force, this composite generated a maximum output voltage of 1.32 V, a current of 0.61 µA, and a power of 0.66 µW. The study highlights the suitability of these materials for harvesting energy from human motion, such as walking or bending, where uniaxial tensile strains are common. In this regard, the ZnO nanoparticles acted as nucleating agents, promoting the formation of the piezoelectric β-phase in PVDF without requiring complex post-processing (*e.g.*, electrical poling or stretching); CNTs improved electrical conductivity and facilitated charge distribution within the polymer matrix, enhancing the overall piezoelectric response; whereas, the PVDF matrix provided flexibility, lightweight properties, and ease of processing into films, making the composite suitable for wearable and motion-driven energy harvesters. The synergistic effect of ZnO and CNT fillers resulted in a significant boost in β-phase content and piezoelectric output compared to neat PVDF or single-filler composites. Another study developed a high-performance, lead-free piezoelectric nanogenerator using PVDF reinforced with surface-modified potassium sodium niobate (SM-KNN) nanorods *via* the electrospinning method.^343^ While the addition of SM-KNN reduced the electroactive β-phase fraction of PVDF compared to pure electrospun PVDF, the composite with 3% SM-KNN exhibited remarkable piezoelectric performance, generating a high output voltage of ∼21 V, a current of ∼22 µA, a current density of ∼5.5 µA cm^−2^, and a power density of ∼115.5 µW cm^−2^ under low pressure (1.1 kPa). This performance significantly surpassed that of composites with unmodified KNN and other reported lead-free nanogenerators, demonstrating the effectiveness of the surface modification strategy. The silane modification reduced the agglomeration of KNN nanorods, promoting their uniform distribution within the PVDF fibers, which allowed for a higher effective loading of the piezoelectric ceramic filler without processing issues. The electrospinning process itself provided *in situ* electrical poling to both the PVDF polymer chains and the aligned KNN nanorods, thus amplifying the net dipole moment and piezoelectric response without requiring a separate poling step. Although the β-phase content was lower, the composite's performance was driven by the increased number of ionic dipoles from the well-dispersed KNN nanorods and their strong interaction with the PVDF matrix. The composite maintained the flexibility and eco-friendliness of PVDF while integrating the high piezoelectric coefficient of KNN, resulting in a sustainable, wearable energy harvester capable of powering small electronics (*e.g.*, LEDs).

**Fig. 30:**
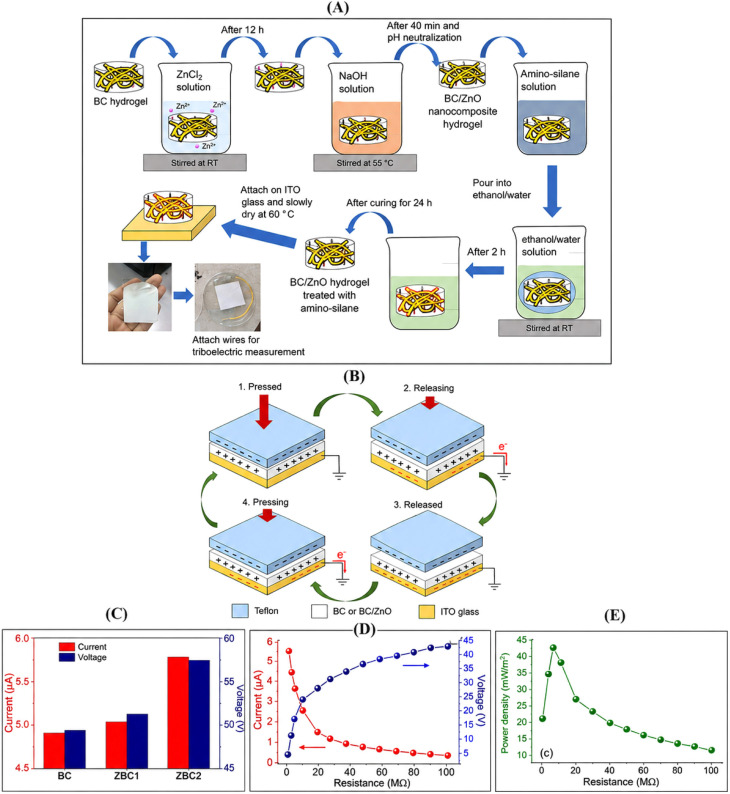
(A) Fabrication of BC/ZnO (ZBC) nanocomposite films: BC hydrogel is impregnated with ZnCl_2_ and treated with NaOH to form *in situ* ZnO nanoparticles, followed by amino-silane surface modification and slow drying on ITO glass to obtain a strongly bonded film without conductive adhesives. (B) Operating principle of a single-electrode TENG in vertical contact-separation mode: contact between the BC-based film and Teflon induces triboelectric charge transfer, while separation drives electron flow through the external circuit, producing alternating current under repeated pressing and releasing. (C–E) Output performance of BC, ZBC1, and ZBC2 films: (C) open-circuit voltage and short-circuit current comparison, with ZBC2 delivering the highest values (57.6 V and 5.78 µA); (D) load-dependent voltage and current; and (E) corresponding power density, with a maximum of 42 mW m^−2^ at 5 MΩ, significantly outperforming pristine BC-based TENGs, reproduced from ref. 344 with permission from American Chemical Society, copyright 2020 (License No. 6297100417052).

Polymer nanocomposites containing hybrid nanoparticles have also been used in various biomaterial-based energy-harvesting applications. For example, a high-performance biocompatible triboelectric nanogenerator (bio-TENG) was developed using bacterial cellulose (BC) and a BC/ZnO nanocomposite (ZBC).^344^ The key innovation was a novel fabrication method that preserved the native 3D nanostructure and high crystallinity (cellulose Iα) of BC by slowly drying the BC hydrogel directly onto an ITO (indium tin oxide) substrate, eliminating the need for destructive dissolution-regeneration processes and conductive adhesives. To further enhance performance, ZnO nanoparticles were incorporated into the BC matrix, creating a nanocomposite that increased surface roughness and introduced piezoelectric-enhanced triboelectrification. However, the ZBC film required surface modification with amino-silane to achieve strong adhesion to the ITO substrate. The optimized ZBC2 nanocomposite (with higher ZnO loading) generated a maximum open-circuit voltage of 57.6 V, a short-circuit current of 5.78 µA, and a peak power density of 42 mW m^−2^, significantly outperforming previously reported cellulose-based TENGs. Surface modification with an amino-silane enabled strong bonding between the hydrophobic ZnO-containing composite and the hydrophilic ITO substrate, ensuring device durability and efficient electron transport without additional interfaces. Fig. 30 illustrates this integrated approach, which preserves BC's 3D nanostructure, eliminates adhesive interfaces, and leverages ZnO nanocomposites to enhance triboelectric output for biocompatible energy harvesting.^344^

### 5.10. Separation membranes

Hybrid polymer nanocomposite membranes are increasingly used to tune both the bulk transport pathways (porosity, pore-size distribution, and tortuosity) and the surface properties (hydrophilicity, charge, and roughness) that govern selectivity and fouling. In practice, the performance gains depend less on the mere presence of nanofillers and more on how well their chemistry, size, crystalline phase, and dispersion state are controlled within the polymer matrix.^291,345^ In membrane-based purification, hybrid polymer nanocomposites significantly improve permeability, solute selectivity, mechanical robustness, hydrophilicity, antifouling resistance, and thermal stability.^277^ A representative example is the incorporation of TiO_2_ nanoparticles into polyethersulfone (PES) nanofiltration membranes. When TiO_2_ grade and crystallography were varied (P25 (20 nm, 80% anatase/20% rutile), PC105 (15–25 nm, anatase), and PC500 (8 nm, anatase)), membrane behavior differed significantly: P25 dispersed more uniformly and produced stronger flux recovery and improved resistance to protein fouling, whereas the ultra-small PC500 tended to agglomerate at higher loadings, which reduced its benefit despite its high surface area. This comparison emphasizes that nanoparticle phase composition and aggregation propensity can dominate the net antifouling outcome, even when the base polymer and fabrication route are unchanged.^346^

**Fig. 31:**
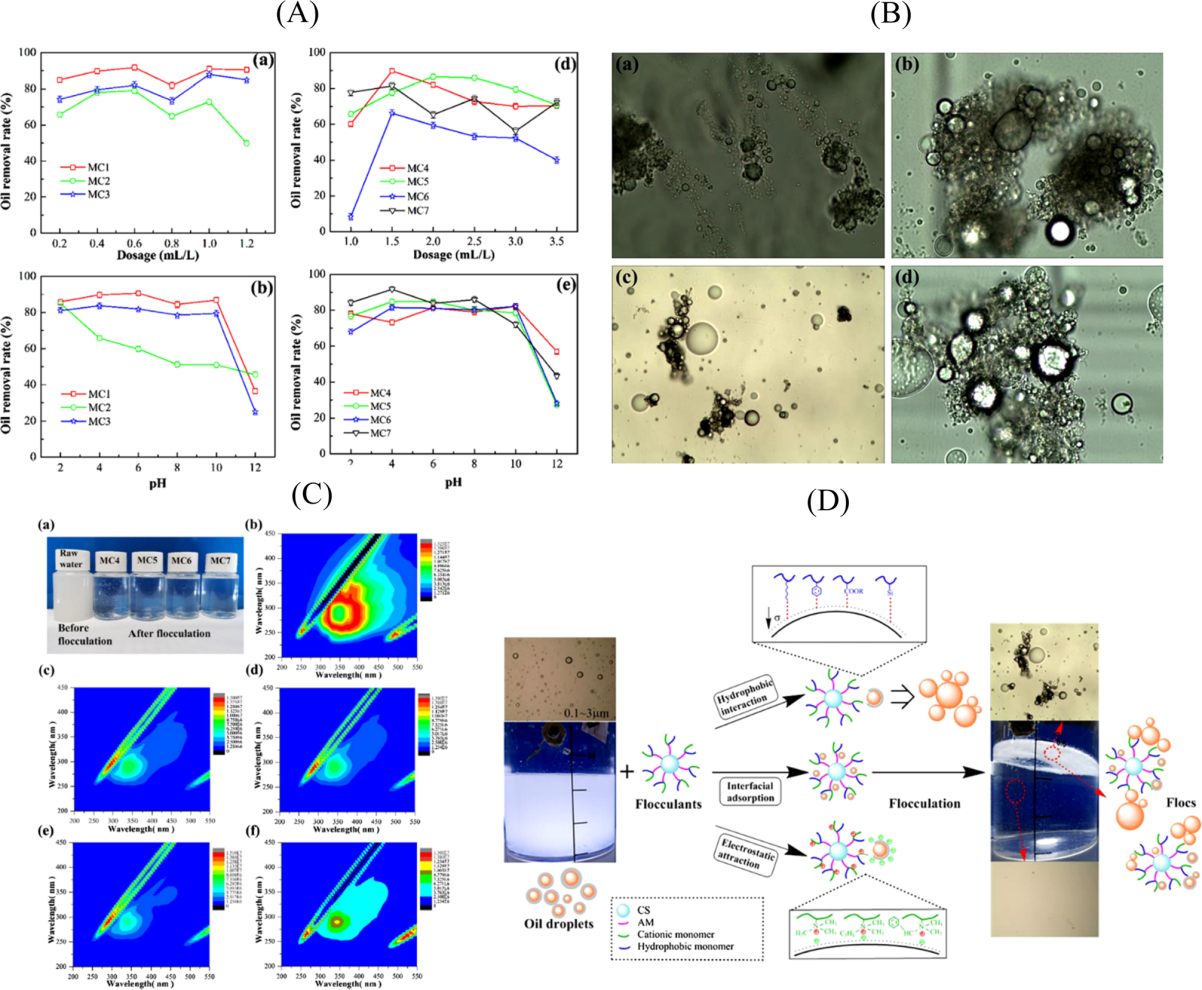
(A) Oil removal efficiency as a function of flocculant dosage and pH for chitosan grafted with different cationic and hydrophobic monomers, with MC1 (DMC-DPL-grafted chitosan) exhibiting the highest oil removal efficiency (∼91.8%) at a low dosage of 0.6 mL L^−1^. (B) Optical micrographs of flocs formed after flocculation, illustrating the formation of dense and compact aggregates driven by hydrophobic association and polymer-bridging effects. (C) 3D excitation–emission matrix (EEM) fluorescence spectra of raw emulsified oily wastewater and treated supernatants, demonstrating effective removal of PAHs and MAHs. (D) Schematic illustration of the proposed flocculation mechanism, highlighting the synergistic roles of electrostatic attraction, interfacial adsorption, and bridging aggregation. Panels (A–C) reproduced from ref. 347 with permission from Elsevier, copyright 2021 (License No. 6297101184852). Panel (D) reproduced from ref. 347 with permission from Elsevier, copyright 2021 (License No. 6297101366740).

Beyond oxide selection, polymer-grafted nanoparticles offer a robust means to suppress nanofiller aggregation and reduce leaching by enhancing nanoparticle-polymer compatibility. For instance, polymer-grafted ZnO (PVP-g-ZnO and P(VP-AN)-g-ZnO) blended into PES *via* non-solvent induced phase separation produced ultrafiltration membranes with improved dispersion, higher hydrophilicity and water flux, and strong antibacterial activity (reported at only 0.5 wt% ZnO) while maintaining PEG rejection; the copolymer-grafted ZnO also improved antifouling stability during operation.^299^ Synergistic effects can be further amplified using hybrid nanofillers. A PES mixed-matrix nanofiltration membrane incorporating a ZnO/MWCNTs nanocomposite has been reported to exhibit antibiofouling behavior, attributed to combined nanostructure-driven transport changes and photocatalytic/antimicrobial activity.^345^ Likewise, a series of modified chitosan (MCS) flocculants was developed by grafting cationic and hydrophobic monomers onto chitosan through low-pressure ultraviolet (UV)-initiated copolymerization for the treatment of emulsified oily wastewater.^347^ The results, summarized in Fig. 31, demonstrate that this polymer nanocomposite strategy is highly effective. Grafting cationic monomers, such as methylacryloxyethyl trimethyl ammonium chloride (DMC), substantially increased the positive charge density of the flocculants, enabling strong electrostatic attraction and charge neutralization with negatively charged oil droplets over a wide pH range (2–10), thereby overcoming the intrinsic limitations of pristine chitosan under alkaline conditions. Concurrently, the incorporation of hydrophobic monomers, particularly vinyltriethoxysilane, strengthened hydrophobic interactions, reduced oil–water interfacial tension, and significantly enhanced demulsification efficiency. This dual functionalization yielded excellent oil removal performance, achieving up to 91.75% removal at an ultralow dosage of 6 mg L^−1^, outperforming unmodified chitosan and singly functionalized flocculants. The nanocomposite architecture promoted synergistic flocculation mechanisms, including electrostatic attraction, hydrophobic association, and adsorption-bridging, leading to the efficient removal of diverse oil components, including alkanes, cycloalkanes, and aromatic hydrocarbons. Overall, the engineered chitosan-based polymer nanocomposite represents a highly efficient, low-dosage, pH-adaptive flocculant for the treatment of emulsified oily wastewater.

Finally, upstream nanocomposite-enabled pretreatment can directly support membrane separations by reducing oil loading and foulant strength before the membrane step. Modified chitosan flocculants prepared by grafting cationic and hydrophobic monomers have been shown to operate across a wide pH window and to achieve high oil removal at low dosage *via* electrostatic attraction, hydrophobic association, and bridging aggregation, thereby meaningfully reducing the fouling burden in subsequent membrane filtration.

Overall, the incorporation of hybrid nanoparticle–polymer nanocomposites can deliver multifunctional, lightweight performance across domains (from aerospace and construction to biomedicine, environmental remediation, and energy devices), but their translation into real-world products ultimately depends on robust dispersion/interface control, scalable processing, and safety- and sustainability-aware design choices.

## 6. Safety and regulatory considerations

### 6.1. *In vitro* and *in vivo* toxicity of hybrid nanoparticle systems

Table 9 presents the representative *in vitro* and *in vivo* toxicity/biocompatibility outcomes reported for hybrid nanoparticle–polymer nanocomposites. Rigorous toxicological evaluation is essential for polymer nanocomposites containing hybrid nanoparticles, particularly in biomedical products, because combining polymers, lipids, and inorganic or carbon-based components can alter nano-bio interactions, biodistribution, degradation pathways, and clearance relative to single-component systems. Toxicity is rarely dictated by composition alone; it is shaped by particle size distribution, surface chemistry and charge, colloidal stability in physiological media, dissolution or ion release (for metal-based constituents), and the identity of the adsorbed biomolecular corona formed *in vivo*, which can modulate immune recognition and organ sequestration.^348^

**Table 9:** Representative toxicity and biocompatibility outcomes of polymer nanocomposites incorporating hybrid nanoparticles

Hybrid nanocomposite system (hybrid type)	Model/route	Dose/duration	Primary readouts	Key toxicity findings	Ref.
PLGA (±PEG) nanoparticles with embedded SPIONs, loaded with gemcitabine (Gem)	*In vitro* (MCF-7 cells)	Gem equivalent to 0.01–10.3 µM; 48 h	Cell viability/cytotoxicity; enhancement ratio (ER)	SPION-PLGA-Gem showed the highest cytotoxicity (ER = 1.89); PLGA-Gem also increased cytotoxicity (ER = 1.53); SPION-PLGA (without drug) was non-toxic (<10% cell death)	257
Lipid–polymer hybrid nanoparticles (various compositions)	*In vitro*; small-animal *in vivo*	Dose-dependent	Acute toxicity/tolerability; circulation; immune interactions	Generally exhibited lower acute toxicity and improved tolerability than free drug; lipid composition and PEGylation influenced immune recognition and circulation half-life, highlighting the need for formulation-specific evaluation	352
PEG-decorated GO nanocomposite	*In vitro* testing and stability assessment in PBS (pH 5.5 and 7.4)	2 mg mL^−1^; 96 h	Cytotoxicity/biocompatibility; colloidal stability; surface charge	No significant toxicity under the tested conditions; mildly negative zeta potential (∼−13.9 mV) contributed to improved colloidal stability and reduced phagocytosis tendency	219
Chitosan-based scaffolds with AgNPs	*In vitro* cytotoxicity assays (L929, MC3T3, HDFa, *etc.*)	Typically 24–72 h incubation	Cell viability/cytocompatibility; irritation observations	Generally demonstrated good cytocompatibility (>80–93.5% viability) with low toxicity; skin irritation was reported only at high AgNP loadings in some film formulations	353
Chitosan/gelatin cryogels with AgNPs and tannic acid	*In vivo* full-thickness infected wound model (mice)	Up to 14 days	Local tolerance; wound healing; infection control	Exhibited effective bactericidal activity, promoted hemostasis, and showed no observable toxicity	353
Chitosan/sericin hydrogels with AgNPs and lupeol	*In vivo* infected full-thickness wound model (Sprague–Dawley rats)	—	Local tolerance; wound closure	Accelerated wound closure without causing adverse effects	353

Cell-based assays remain the first line of evaluation and should include cytotoxicity, membrane integrity/hemolysis, oxidative stress and reactive oxygen species generation, inflammatory signaling and cytokine release, and genotoxicity where relevant. Interpretation requires careful attention to realistic dose metrics and exposure conditions, because nanoparticle sedimentation, agglomeration, and assay interference can distort apparent potency; these issues are widely recognized as persistent barriers to predictive *in vitro* nanotoxicology.^349,350^ Reporting of dispersion protocols, media composition, and physicochemical properties under test conditions is therefore not optional for reproducibility and cross-study comparability, consistent with minimum-information reporting recommendations for bio-nano experiments.^351^

Animal studies remain necessary to assess systemic immunotoxicity, pharmacokinetics, and organ-level effects, including hepatosplenic accumulation, renal clearance constraints, and inflammatory responses. For clinically oriented hybrid nanocarriers, published data often indicate acceptable tolerability at therapeutic doses when formulations are engineered for stability and biocompatibility, but material- and dose-dependent liabilities are common. For example, PLGA (±PEG) nanoparticles embedding superparamagnetic iron oxide nanoparticles (SPIONs) can be tolerated in rodents when appropriately formulated, whereas poorly stabilized or higher iron oxide burdens may increase hepatic retention and oxidative stress.^257^ LPHNPs are frequently reported to reduce acute toxicity compared with free drugs, yet lipid composition and PEG density can alter immune recognition, circulation time, and complement activation risk, requiring formulation-specific evaluation.^352^ Carbon- and metal-containing hybrids can show even greater variability: PEGylated graphene oxide and silver-containing wound materials may be well tolerated within certain windows, but responses depend strongly on surface functionalization, oxidation state, release kinetics, and local exposure levels.^219^

Two practical conclusions follow from the current evidence base. First, surface functionalization (for example, PEG, polysaccharides, polydopamine-like coatings, or zwitterionic chemistries) is often decisive in reducing nonspecific protein adsorption, macrophage uptake, and immune activation, thereby improving biocompatibility and circulation.^348^ Second, dose escalation, time-resolved blood chemistry, and multi-organ histopathology remain essential to detect sub-acute accumulation in liver, spleen, kidney, and lung, as well as delayed inflammatory or fibrotic signatures that may not be apparent in short-term assays.

### 6.2. Nanoparticle tracking and biodistribution

Reliable biodistribution measurements are central to both efficacy and safety assessment because organ accumulation and persistence often determine the dominant risk profile for hybrid nanomaterials. Common strategies include fluorescent labeling for qualitative real-time imaging, radiolabels such as ^64^Cu or ^89^Zr for quantitative PET (positron emission tomography)/SPECT (single photon emission tomography) tracking, and elemental or magnetic signatures for ICP-MS, MRI, or related modalities.^354^ Each method has known limitations: fluorescence can be attenuated and may decouple from the carrier if labels leach; radiolabeling requires specialized infrastructure and careful control of label stability; elemental analysis is often terminal and typically lacks spatial resolution at the tissue microdomain level. Consequently, orthogonal approaches that combine at least one whole-body technique (PET/SPECT or MRI) with ex vivo quantification (ICP-MS or gamma counting) are recommended to strengthen conclusions about clearance routes, organ retention, and long-term fate. For hybrid systems, tracking strategies should also address whether the measured signal reports intact carrier, released inorganic fraction, or degradation products, since polymer erosion, lipid exchange, and ion dissolution can separate components over time. This distinction becomes critical when interpreting “safe” circulation profiles that may mask the delayed accumulation of a persistent inorganic phase.

### 6.3. Regulatory pathways and quality requirements

Regulatory agencies such as the FDA (Food and Drug Administration) and EMA (European Medicines Agency) evaluate nanomedicines under existing pharmaceutical and device frameworks but place additional emphasis on nano-specific critical quality attributes, including particle size distribution, morphology, surface chemistry, colloidal stability, and biodistribution. FDA guidance encourages early engagement to determine data expectations for products involving nanotechnology,^355^ while EMA reflection papers outline considerations for non-clinical testing, product comparability, and risk-based evaluation for nanotechnology-based medicinal products.^356^ Because assessment is case-specific, seemingly modest formulation changes, such as surface coating density, lipid composition, or manufacturing parameters that shift aggregation state, can trigger additional comparability and safety testing.^355,356^ A broader regulatory-science literature also emphasizes that robust characterization and quality-by-design approaches are central to translation, because *in vivo* performance is highly sensitive to formulation-dependent quality attributes.^357,358^

For polymer nanocomposites and hybrid nanocarriers intended for clinical use, successful translation typically requires: (1) comprehensive physicochemical characterization using multiple orthogonal methods, (2) validated bioanalytical assays that can distinguish intact material from degraded components, (3) GLP (Good Laboratory Practice)-aligned toxicology packages that include immunotoxicity considerations when indicated, and (4) clear biodistribution and clearance profiles that support a coherent risk assessment.^356^ For non-medical applications, regulatory obligations may arise under chemicals legislation when nanoforms are manufactured, released, or disposed of, and recent analyses of European regulation highlight continuing efforts to standardize definitions, measurement methods, and risk assessment tools for nanomaterials.^359^

### 6.4. Life-cycle, occupational, and environmental considerations

Safety evaluation should extend beyond intended use to include realistic exposure scenarios during manufacturing, machining, use, weathering, recycling, and disposal. Evidence from machining studies indicates that mechanical processing of polymer nanocomposites often releases aerosols dominated by polymer matrix fragments that contain embedded nanofillers or present protruding nanostructures, rather than large quantities of free isolated nanoparticles. Release behavior depends on process parameters and filler type, supporting a scenario-based approach to exposure control during drilling, sawing, sanding, or cutting.^360,361^ From a governance perspective, contemporary reviews of European nanomaterial legislation emphasize the need to minimize unintended emissions, strengthen standardized characterization and risk assessment methods, and improve implementation and enforcement across the life cycle.^359^ In practice, this supports integrating safer-by-design principles early, selecting hybrid constituents with lower persistence or lower hazard potential where feasible, and implementing engineering controls and exposure monitoring in workplaces that handle nano powders or machine nanocomposite parts.

Dynamic structural stability is a central safety criterion for hybrid nanoparticles because their toxicological identity can change during exposure rather than remain fixed at the as-synthesized state.^362–364^ In polymer nanocomposite products, this issue arises both for intentionally administered hybrid nanocarriers and for fragments generated during processing, weathering, abrasion, recycling, or disposal. Hydrolysis, oxidation, UV exposure, protein adsorption, ionic strength, chloride or sulfide ligands, and pH can alter shell thickness, interfacial bonding, surface charge, aggregation state, dissolution rate, and ion release. Consequently, a particle that shows low apparent hazard when intact may generate a more reactive or bioavailable form after partial matrix or shell degradation.^363–366^

Core–shell hybrids are particularly sensitive to this time-dependent behavior. Polymeric shells improve dispersion, matrix compatibility, and, in many cases, passivate reactive inorganic cores such as Ag, Cu, ZnO, TiO_2_, or quantum dots. In biological media, aliphatic polyester shells such as PLGA and PLA undergo hydrolytic chain scission at rates controlled by pH, temperature, molecular weight, crystallinity, and local water uptake.^367,368^ Loss of shell integrity can expose the inorganic core to proteins, cells, salts, or environmental ligands, thereby changing ion release, redox activity, ROS formation, and cellular uptake. Therefore, toxicity assays performed only on freshly prepared particles may underestimate risk when the dominant hazardous entity forms after delayed shell degradation or matrix aging.^362,366^

Electronically coupled bimetallic hybrids also require time-dependent analysis. Contact between metallic domains with different redox potentials can modify oxidation and dissolution through electron-transfer and galvanic-coupling effects. However, Ag–Au systems show that the direction and magnitude of this effect are architecture-dependent: Au@Ag core–shell particles can suppress Ag^+^ release through electron compensation,^369^ whereas uncoated Ag nanoparticles can release Ag^+^ slowly during storage and show increased toxicity as dissolution proceeds.^370^ Ag–Au/PTFE nanocomposites further demonstrate that alloying, matrix environment, and composition can regulate Ag release.^371^ Thus, safety assessment should not assume a fixed bimetallic composition, but should measure metal speciation and phase-selective dissolution under relevant exposure conditions.

Additional transformation pathways include lipid exchange and protein-corona formation in lipid–polymer hybrids, which can alter cellular uptake and clearance;^372,373^ changes in surface valence states of redox-active iron oxide cores, which may affect Fenton-type ROS generation under oxidative conditions;^374,375^ and photoactivation of semiconductor components such as ZnO and TiO_2_ during environmental exposure. Simulated solar UV has been shown to increase ZnO nanoparticle toxicity through ROS generation and photo-induced dissolution,^376^ while Ti-bearing nanomaterials can be released and transported through wastewater systems.^377^ These examples indicate that long-term risk depends not only on the initial composition but also on the evolving architecture, the surrounding medium, and the released ionic or particulate species.

Accordingly, safety evaluation of hybrid nanoparticle–polymer nanocomposites should include time-resolved physicochemical characterization in application-relevant media. Characterization should track particle size distribution, aggregation, shell integrity, core exposure, metal speciation, ion release kinetics, oxidation state, and surface chemistry using complementary methods such as electron microscopy, spectroscopy, ICP-MS, single-particle ICP-MS, and dissolution assays.^362,366^ Biomedical systems should be tested in relevant serum, lysosomal, or simulated physiological media, whereas construction, packaging, coating, and environmental systems should be assessed after weathering, wastewater, freshwater, soil-porewater, or landfill-relevant exposure. Incorporating these transformation data into hazard assessment would more directly connect hybrid architecture, long-term stability, toxicity, and environmental impact.^363,364^

## 7. Limitations and challenges

### 7.1. Synthesis and processing challenges

Achieving uniform and stable nanoparticle dispersion remains a central challenge,^378^ as agglomeration often undermines improvements in mechanical, thermal, and electrical properties. Owing to their high surface energy and van der Waals interactions, nanoparticles tend to cluster. This problem is exacerbated in hybrid systems containing multiple nanoparticle types, each with distinct surface chemistry and interaction preferences that often favor self-association over integration into the polymer matrix.^147,379^ Such non-uniform distributions create stress concentration points that can lead to premature failure and inconsistent property profiles throughout the material.^380^

The performance of polymer nanocomposites depends strongly on the interfacial adhesion between nanofillers and the matrix. Many high-performance nanoparticles are inherently incompatible with hydrophobic polymers. Weak interfacial bonding reduces stress transfer efficiency, lowers reinforcement effectiveness, and facilitates debonding under load.^381^ Surface functionalization can improve compatibility, but it increases synthesis complexity and cost.^147,382^ Optimizing functionalization in hybrid systems is particularly difficult, as distinct surface chemistries must be simultaneously compatible with the same polymer matrix.^383^

Scaling laboratory synthesis methods to industrial production introduces further limitations: Sol–gel methods produce high-purity, homogeneous materials but are energy- and time-intensive. Gelation, aging, and drying stages are slow and prone to shrinkage and cracking. Moreover, the process is susceptible to parameters such as pH, temperature, and precursor concentration, complicating reproducibility at large scales.^384,385^*In situ* polymerization can achieve good dispersion but often requires specific monomers, catalysts, and reaction conditions unsuitable for many polymers.^386^ Controlling molecular weight and polymer architecture in the presence of nanoparticles is difficult, and removing residual monomers or catalysts adds an extra processing step.^387,388^ Mechanical blending/ball milling is simple but introduces contaminants from the grinding media and vessel, compromising purity. It provides limited control over particle size and morphology, consumes high energy, and generates heat that may degrade heat-sensitive polymers.^389^ The process is also energy-intensive and can cause excessive heat generation, potentially degrading heat-sensitive polymers or modifiers.^390^ Melt intercalation is attractive for thermoplastics but hampered by the high viscosity of polymer melts, which hinders exfoliation and uniform dispersion of fillers such as layered silicates. Shear forces during extrusion may fracture high-aspect-ratio fillers such as CNTs, reducing their reinforcing capability.^391^ Furthermore, non-polar polymers such as polypropylene (PP) and polyethylene (PE) require compatibilizers for effective intercalation, adding cost and complexity.^392^

### 7.2. Characterization and performance limitations

Establishing reliable structure-property relationships in hybrid nanocomposites requires advanced and costly techniques, including high-resolution TEM, XPS, and AFM.^393^ This lack of accessible characterization tools slows material optimization. Determining the 3D spatial distribution, orientation, and interfacial quality of multiple nanofillers within a matrix remains very difficult.^394^ Long-term performance under service conditions is another concern. Exposure to UV radiation, moisture, oxygen, and temperature fluctuations degrades both the polymer matrix and filler-matrix interfaces.^395^ UV radiation can cause chain scission and discoloration, while moisture promotes hydrolysis, especially in composites with natural fibers or hydrophilic fillers.^396,397^ A recent study^398^ confirmed that environmental degradation can significantly affect both structural integrity and biocompatibility of polymer systems. In biomedical and food-packaging applications, nanoparticles may leach from the matrix, compromising functionality and raising toxicity and environmental risks.^399–401^ The lack of standardized test methods for assessing leaching across diverse product categories underscores the need for harmonized evaluation protocols. Under sustained or cyclic loading, composites may exhibit creep deformation or fatigue failure. The influence of hybrid nanofillers on these time-dependent behaviors is still poorly understood and requires systematic long-term studies.^402,403^

Hybrid nanocomposites also involve property trade-offs. Enhancing tensile strength and modulus may reduce toughness and impact resistance, rendering materials more brittle.^404,405^ Increasing filler loading can improve thermal and electrical conductivity but simultaneously raise viscosity, limiting processability in conventional techniques such as injection molding or resin transfer molding.^406^ Furthermore, incorporating functional nanofillers for antimicrobial activity or flame retardancy may compromise optical clarity or biocompatibility.^23^

### 7.3. Economic, environmental, and safety concerns

The high cost of producing high-quality nanomaterials remains a critical barrier to their large-scale adoption, particularly in cost-sensitive sectors such as automotive and construction.^407^ Additional steps required for surface functionalization, dispersion, and specialized synthesis further increase production costs.^407,408^ The potential toxicity of certain nanoparticles is a serious concern throughout their life cycle, from manufacturing through use and disposal. Inhalation of nanoparticle dust poses occupational health risks, necessitating strict safety protocols.^54^ Moreover, the ecotoxicological impacts of nanoparticle release from aged or degraded products into soil and aquatic systems remain poorly understood and require further study.^409^ Nanoparticle toxicity is mediated by oxidative stress, inflammation, and DNA damage, while current risk-assessment frameworks remain inadequate for composites containing multiple nanoparticle types.^410,411^ The hybrid nature of polymer-nanoparticle composites creates a complex waste stream that is difficult to recycle.^412^ Conventional mechanical recycling often fails, as it can exacerbate nanoparticle agglomeration.^413,414^ Advanced chemical recycling methods are needed to separate and recover valuable components. In addition, nanoparticles may contaminate existing polymer recycling streams.^413^ Hybrid composites complicate separation processes and require novel chemical or upcycling strategies to maintain material value.^415^ While the use of biodegradable polymer matrices has been proposed as a solution, the persistence of embedded nanoparticles and their influence on biodegradation and ecosystems remain critical unresolved questions.^416^ The regulatory framework governing nanomaterials and nanocomposites is still under development. Standardized protocols for assessing safety, efficacy, and environmental impact are lacking.^417^ This regulatory uncertainty hinders product development and deters investment. Without harmonized international testing standards, manufacturers remain hesitant to commercialize nano-enabled composites at scale.^410^ Establishing clear regulations and harmonized testing standards will be essential for fostering industrial confidence and enabling market adoption.^418^

### 7.4. Future research needs

Several knowledge gaps should be addressed to accelerate the development and adoption of hybrid nanocomposites. There is a need for robust multi-scale models capable of predicting final composite properties from constituent characteristics, morphology, and interfacial interactions. Such models would reduce reliance on costly and time-consuming trial-and-error experimentation.^419,420^ New, scalable, and effective strategies are required to tailor interfacial interactions in hybrid systems to maximize synergistic effects.^421^ The application of AI and machine learning (ML) remains in its early stages. Progress depends on creating large, high-quality datasets for model training, along with strategies that enable inverse design rather than simple property prediction.^422^ ML-driven workflows can accelerate polymer design, guide process optimization, and even achieve inverse design through robotics-assisted discovery of biodegradable substitutes.^423^ However, challenges remain in data availability, model interpretability, and validation under industrial conditions. Future research may prioritize the development of “green” hybrid nanoparticles through sustainable synthesis routes. Integrating these fillers into biodegradable or recyclable polymer matrices will be critical to establishing a circular economy approach from the outset.^424,425^ Emerging approaches in polymer upcycling and sustainable informatics offer opportunities to design recyclable, environmentally benign nanocomposites.^415^

Overall, while hybrid nanoparticle-based polymer nanocomposites hold substantial promise, overcoming challenges in processing, performance, cost, safety, and sustainability remains the key to realizing their industrial potential. Interdisciplinary collaboration, supported by advances in modeling, AI, green synthesis, and standardized safety regulations, will be essential to translate laboratory advances into safe, reliable, and economically viable products.

## 8. Emerging trends and future perspectives

### 8.1. Self-healing and smart nanocomposites with hybrid nanofillers

Hybrid nanofillers—engineered combinations of two or more nanoscale phases—are enabling polymer matrices that combine structural reinforcement with on-demand functions such as corrosion protection, electrical responsiveness, optical reporting of damage, and autonomous repair. By coupling complementary chemistries and morphologies, hybrid systems can improve nanofiller dispersion, interfacial adhesion, and stress transfer while introducing responsive mechanisms that activate in response to specific stimuli, such as pH, redox potential, temperature, light, or moisture. Rapid progress across self-healing epoxy coatings, shape-memory systems, and multi-stimuli “smart” polymers, as well as the growing use of metal–organic frameworks (MOFs), polydopamine (PDA) gates, and conductive polymers as active nanocontainers or triggers.^426–428^ For example, decorating MWCNTs with TiO_2_ can improve dispersion and matrix wetting without eroding the intrinsic stiffness and aspect ratio advantages of CNTs, with documented gains in mechanical performance and corrosion resistance when embedded in epoxies. In thermo-reversible self-healing matrices, adding such hybrid nanoparticles increased adhesion strength by 317%.^429^ Beyond mechanical reinforcement, hybrid nanofillers enable “active” repair and protection. MOF-based nanocontainers (notably zeolitic imidazolate framework (ZIF)-8) store and release corrosion inhibitors or catalytic species under local pH shifts at a crack tip; PDA shells can act as pH-sensitive gates and adhesion primers; conductive components such as polypyrrole or polyaniline provide redox-assisted passivation and photothermal heating for accelerated healing. PPy@ZIF-8 self-healing epoxy coatings, ZIF-8@PDA hybrids for inhibitor release and adhesion enhancement, and MOF-polymer hybrid microcapsules that combine robust storage with rapid stimulus-triggered delivery.^430^ Stimuli-responsive hybrid nanoparticles also underpin targeted drug delivery. pH-responsive constructs exploit the acidic tumor microenvironment to trigger structural disassembly or drug release, while redox-responsive designs use elevated intracellular glutathione to cleave disulfide linkages and release payloads. Advances in pH, redox, ROS, enzyme, and hypoxia triggers demonstrate precise release kinetics and improved therapeutic indices in preclinical models.^400^ These directions align with a broader move toward long-lasting, repairable, and resource-efficient materials that support circular-economy goals.^401^Table 10 summarizes representative hybrid nanoparticle systems used in self-healing and stimulus-responsive polymer nanocomposites/coatings, highlighting the matrix, key reported functions/metrics, and the dominant healing or protection trigger.

**Table 10:** Representative hybrid nanoparticle systems reported for self-healing and smart polymer nanocomposites/coatings

Hybrid nanoparticles	Polymer matrix	Smart functionality (key metric)	Healing trigger/mechanism	Reference
MWCNTs) + MnO_2_	Polymer-clay nanocomposite hydrogel (PCN hydrogel)	Stretchable, self-healing supercapacitor hydrogel; elongation at break up to 6511%; enhanced electrochemical performance	Hydrogen bonding	437
ZIF-8@PDA (polydopamine-coated ZIF-8)	Epoxy resin matrix	Enhanced corrosion resistance and self-healing; improved thermal stability; reduced water uptake	Hydrogen bonding/PDA-assisted interfacial adhesion	438
PG (poly tannic acid modified GO) + BTA (benzotriazole) @ZIF-8/PM (polymeric matrix)	Waterborne epoxy resin (WEP)	Triple-function smart anti-corrosion with self-healing ability, effective long-term performance in severe environments (*e.g.*, seawater)	pH-triggered release of corrosion inhibitors from ZIF-8 nanocontainers	439
Phen(phenanthroline)-Tpp(tripolyphosphate ions) @MTNs(mesoporous TiO_2_ nanocontainers)-PDDA(poly dimethyl diallyl ammonium chloride)	Epoxy resin	Smart monitoring of color changes due to corrosion, a visual indicator of damage, and self-healing	pH-triggered release of Phen and Tpp corrosion inhibitors	440
ZnO/RGO/PANI	Epoxy resin	233% increase in adhesion strength, improved durability	Redox-assisted passivation *via* PANI	441
BTA@MoS_2_/hydroxyapatite/ZIF-8	Epoxy-resin	Multi-functional smart anti-corrosion, enhanced self-healing	Damage-triggered release of BTA from ZIF-8	442
ZIF 10/ZIF 67 (Zn/Co-bimetallic MOF)	Epoxy coating	Improved corrosion protection, self-healing at 0.15 wt% loading	pH-stimulated protective response of MOF nanocarrier	443
FSiO_2_@sND (fumed silica@surface-functionalized nanodiamond)	Epoxy coating	pH-sensitive dual self-healing/barrier anticorrosion; inhibitor released at 0.33 wt%; +138% barrier efficiency after 5 h immersion	An alkaline pH triggers the release of the APTES (3-aminopropyltriethoxysilane) inhibitor	444
Fe_3_O_4_@CB^6^ (cucurbit^6^ uril-modified Fe_3_O_4_)	P(AM-G) (polymer network)	Photothermal response; ∼100% healing after 48 h; stretchable, injectable, and biocompatible	NIR photothermal heating and CB^6^-mediated host–guest interactions	445
CNTs-SiO_2_	Epoxy resin	Superhydrophobic coating (WCA (water contact angle) 159.4° at 30 wt% CNTs-SiO_2_); improved anticorrosion	Thermally activated shape-memory healing (*T* > *T*_g_)	446
F_*x*_SiO_2_/PHFBA (polyhexafluorobutyl acrylate)–SiO_2_	Waterborne epoxy resin (WEP)	Enhanced corrosion resistance, hydrophobicity, and durability	Thermally activated healing (*T* > *T*_g_; shape-memory effect)	447
GO-SiO_2_	RTV (room-temperature-vulcanizing) silicone rubber	Improved corrosion resistance (∼10× *vs.* uncoated steel), hydrophobicity (WCA 128°), and adhesion; durable immersion performance and self-healing	Moisture-induced pH change and thermal/NIR stimulus aid self-healing	448
PEG (polyethylene glycol)/Fe_3_O_4_-SA (sodium alginate)	Polyamide resin and epoxy	Superhydrophobicity, room-temperature self-healing, and durability	Hydrogen bonding	449

Design practice increasingly follows four guidelines. First, use hybrid filler architectures (*e.g.*, 1D–2D or MOF-polymer combinations) to decouple dispersion control from functional roles, thereby achieving synergistic reinforcement and responsiveness.^26,431^ Second, tune inhibitor release to local micro-chemistries using stimuli-responsive nanocontainers (*e.g.*, ZIF-8 or PDA-gated carriers) so that corrosion inhibitors are deployed at damaged sites.^432,433^ Third, target filler loadings near the electrical percolation threshold to achieve conductive networks while keeping viscosity and printability/melt processability within workable ranges.^434,435^ Fourth, report healing and protection with standardized metrics: healing efficiency over multiple cycles; electrochemical impedance spectroscopy emphasizing low-frequency impedance for barrier performance (as per ISO 16773); durability of wetting states *via* sustained contact and roll-off angles under abrasion or immersion; and adhesion retention after environmental aging using pull-off tests (ASTM D4541).^436^

### 8.2. Biodegradable and sustainable nanocomposites with hybrid nanoparticles

Biodegradable polymers are advancing as substitutes for petro-plastics to mitigate persistent waste and microplastics pollution.^450^ Yet many promising biopolymers exhibit modest strength and barrier performance; embedding hybrid nanofillers (*e.g.*, Cu, GO, CNTs, Ag, ZnO, TiO_2_, S, and nanoclays) can increase tensile modulus/strength, improve thermal stability and moisture/oxygen barrier performance, and impart antimicrobial activity.^451^ Bio-derived hybrid fillers can retain biodegradability while expanding function. For example, PVA/CNF (cellulose nanofibers)/AgNP films exhibit higher tensile strength, reduced water vapor permeability, and stronger antimicrobial activity than neat PVA, consistent with reports on green-synthesized AgNPs.^452–454^ Hybrid GO-ZnO nanofillers in polycaprolactone (PCL) increase tensile properties, accelerate hydrolytic degradation, and provide antibacterial efficacy, supporting applications in bio-packaging. The 0.5 wt% GO-ZnO (1 : 1) can also increase tensile strength, modulus, and elongation, while enhancing degradation and antibacterial activity relative to neat PCL.^455^ Green-route photocatalytic hybrids illustrate “waste-to-resource” concepts aligned with circularity. Phenyl-grafted carbon nitride/TiO_2_ core–shell nanohybrids enable visible-light *Rhodamine B* degradation at 2.3 × 10^−2^ min^−1^ and hydrogen evolution at ≈5800 µmol g^−1^ h^−1^, using inexpensive precursors and mild processing.^456^ Eco-friendly FSiO_2_@sND (fumed silica@nanodiamond) hybrids in epoxy coatings combine self-healing and anticorrosion with benign constituents, supporting durable protection with reduced environmental burden.^444^ In biomedical contexts, biodegradable matrices loaded with hybrid nanoparticles can support regeneration and resorb *in vivo*, thereby avoiding the need for implant removal.^457^ A chitosan-gelatin hydrogel reinforced with modified ZnO (*e.g.*, PDA coating and calcium phosphate (CaP) mineralization) shows antibacterial, biocompatible, and osteoconductive behavior;^458^ broader studies on chitosan/gelatin scaffolds and PDA-coated ZnO support these findings and mechanisms for cell adhesion.^459^ Finally, fully biodegradable energy-storage nanocomposites are emerging. CoMoO_4_ nanofillers in PVA/PVP matrices yield flexible, water-processed electrodes with specific capacitance ≈11.35 F g^−1^ at 1 A g^−1^, 74% retention after 10 000 cycles, and device windows near 1.5 V, showcasing end-of-life-compatible, low-toxicity processing.^460^

### 8.3. AI and ML in polymer hybrid nanocomposites

The spatial distribution of nanoparticles within a polymer matrix is a primary determinant of the multifunctional response of polymer nanocomposites, governing stiffness and strength, transport and barrier behavior, thermal conduction, and failure processes. However, dispersion, clustering, and interphase formation arise from coupled effects of surface chemistry, rheology, processing history, and nanoscale interactions, making nanoparticle distributions difficult to predict and control experimentally at scale.^461^ In this context, AI and ML are increasingly used to complement conventional experimentation and physics-based modeling by extracting structure-process-property relationships from heterogeneous datasets and enabling faster screening of compositions and processing routes.^423,462,463^ ML, broadly defined as algorithms that learn predictive rules from data rather than explicit programming,^464,465^ now supports the development of composite materials across sectors that demand tight property windows and reproducible manufacturing, including aerospace, automotive, biomedical devices, and infrastructure.^423^ These approaches are particularly valuable for polymer hybrid nanocomposites, where multiple filler types, interfacial modifiers, and process parameters expand the design space beyond what can be efficiently explored using trial-and-error workflows.^466,467^

**Fig. 32:**
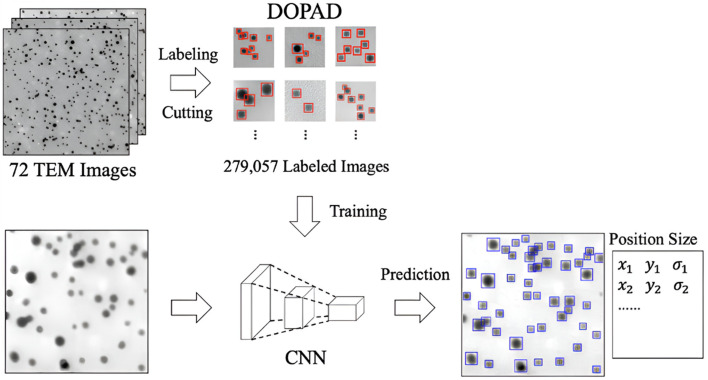
CNN-based supervised learning workflow for nanoparticle detection in polymer nanocomposites. Automated labeling and cropping (DOPAD) converted 72 TEM images into 279 057 labeled sub-images. After training, the CNN localizes nanoparticles in new micrographs and predicts particle locations and size distributions, enabling faster and more consistent microstructural quantification, reproduced from ref. 468 with permission from The Royal Society of Chemistry, copyright 2025.

Recent progress spans three tightly connected themes: microstructure quantification, property prediction, and process optimization. For microstructure analysis, computer vision and deep learning provide scalable routes to quantify dispersion metrics directly from microscopy, reducing subjectivity and improving throughput. Ayush *et al.* introduced nanoNET, an ML platform for predicting nanoparticle distributions in polymer matrices, illustrating how data-driven surrogates can assist dispersion-aware design.^461^ Complementing such efforts, CNN-based pipelines can automate particle recognition and sizing from TEM images, enabling statistically meaningful morphology maps that connect nanoscale organization to macroscopic response.^468^ Beyond polymer-specific examples, deep learning has also demonstrated robust extraction of structure–property linkages from complex microstructures, supporting the broader move toward microstructure-informed modeling (for example, CNN-based analytics in materials microstructures).^469^Fig. 32 shows a CNN-based supervised learning workflow for nanoparticle detection in polymer nanocomposites.

Property prediction is now one of the most mature ML applications in polymer composites and hybrid nanocomposites, with models trained to estimate mechanical response, thermal transport, electrical behavior, and durability using descriptors that encode composition, filler characteristics, and processing. Yasniy *et al.* showed that both neural networks and boosted-tree methods can predict thermal conductivity trends in epoxy composites with different fillers, with useful accuracy for screening and preliminary design.^470^ At the manufacturing level, ML is also increasingly integrated with process simulation and experimental feedback to identify operating windows that satisfy multiple constraints, which is especially relevant for polymer nanocomposites fabricated by additive manufacturing or multi-material printing, where viscosity, cure kinetics, and interlayer bonding must be controlled in parallel^471,472^ In the broader materials community, active-learning and Bayesian optimization strategies provide a principled way to select the next experiment or simulation under uncertainty, accelerating convergence toward target performance while limiting redundant testing.^473,474^

Despite these advances, several barriers still limit the routine industrial deployment of ML-guided design for polymer-hybrid nanocomposites. The most immediate constraint is data quality and completeness. Using the NanoMine resource, Ma *et al.* showed that many polymer nanocomposite datasets remain relatively small and inconsistently annotated; key fields such as processing conditions, dispersion state, and microstructural descriptors are often missing, reducing robustness and limiting transfer to new chemistries and filler combinations. A practical and widely cited near-term objective is the development of standardized, open, and well-curated datasets with more than 10 000 records, with consistent reporting of matrix type, hybrid filler identity, and surface treatment, dispersion metrics, interphase descriptors where possible, and processing history.^475^ Community alignment with machine-actionable data practices, including FAIR (Findable, Accessible, Interoperable, and Reusable) data principles, is expected to improve reuse and model reproducibility,^476^ and emerging efforts toward machine-readable polymer data infrastructures point in the same direction.^476,477^

A second challenge is mechanistic interpretability. Although current ML models can fit complex nonlinear mappings, many remain difficult to interpret physically, limiting their value for hypothesis-driven design. Black-box predictors often fail to clarify how dispersion, interphase thickness, or processing-induced anisotropy drive performance, highlighting the need for improved interpretability for mechanism-based materials engineering.^423^ A promising pathway is the integration of domain constraints and physics-guided learning, including hybrid workflows that combine ML with multiscale simulations and physics-informed formulations to improve extrapolation and reduce dependence on large labeled datasets.^478–480^

Overall, the trajectory of the field suggests that, as data infrastructures mature, experimental/characterization pipelines become more automated, ML-guided design and optimization of polymer hybrid nanocomposites could reach routine industrial technological readiness within the next decade, particularly for property screening, quality monitoring, and process window identification.^423,463,470,475^ Progress toward this goal will depend on interoperable databases, standardized reporting of processing and microstructure, uncertainty-aware decision-making, and models that remain consistent with known composite physics rather than relying only on statistical correlation.

### 8.4. Role of hybrid nanoparticles in next-generation applications

**Fig. 33:**
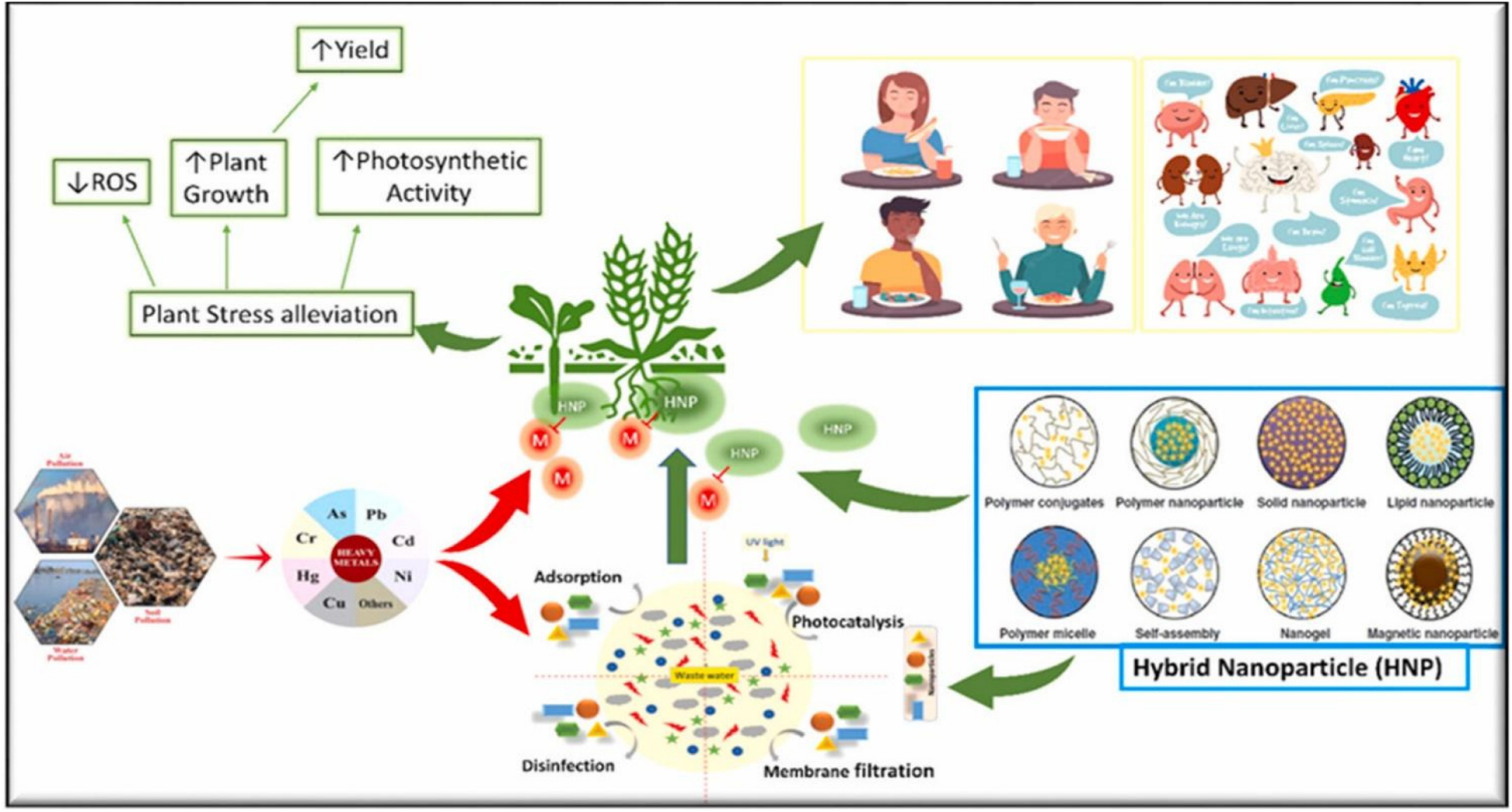
Potential of hybrid nanoparticles in metal-contaminated agroecosystem, reproduced from ref. 498 with permission from Elsevier, copyright 2024 (License No. 6297110494989).

Hybrid nanoparticles are increasingly recognized as enabling platforms for next-generation biomedical, energy, environmental, and manufacturing applications. Their ability to integrate complementary inorganic and organic functionalities within a single construct allows precise tuning of mechanical, chemical, optical, and biological responses. In the treatment of neurodegenerative diseases, hybrid nanocarriers can be engineered to cross the blood–brain barrier, encapsulate and stabilize fragile therapeutics, and provide sustained release. Lipid–polymer hybrids carrying rivastigmine hydrogen tartrate improve delivery in Alzheimer's therapy, while auranofin-loaded chitosan-lipid hybrids demonstrate neuroprotective activity in Parkinson's disease. For migraine management, PLGA-lipid hybrids loaded with propranolol achieve efficient brain targeting *via* intranasal delivery.^481–484^ Beyond small molecules, lipid–polymer hybrids now enable delivery of DNA, RNA, and CRISPR-Cas9 systems. Notably, lipid–polyhydroxyalkanoate hybrids provide sustainable, biodegradable carriers for mRNA therapeutics and vaccines.^485–487^ Hybrid nanoparticles also support wound healing and theranostics. GO-Ag hybrids reinforced in bacterial cellulose enable responsive antimicrobial dressings.^488^ A core–shell construct, PNP@(^131^I-Hypericin), integrates a radiotherapeutic core with a PEG-PCL polymeric shell, offering combined necrosis imaging and targeted therapy for cancer.^489^ In energy devices, PANI-ZrO_2_ hybrids provide synergistic pseudocapacitance, high energy density, and cycling stability for supercapacitors.^490^ Similarly, Al_2_O_3_/TiO_2_ hybrids in PEO/chitosan matrices reduce crystallinity, tune band gaps (from 5.48 eV to 5.01 eV), and enhance AC conductivity, suggesting utility in optoelectronics.^491^ ZnO/NiFe_2_O_4_ hybrids in chitosan-PVP blends improve EMI shielding and magneto-electronic performance, with tunable band gaps and conductivity suitable for supercapacitors.^492^ Hybrid nanostructures offer multifunctionality for pollution control. LBG-AgNP@g-C_3_N_4_ nanosheets combine plasmonic enhancement, visible-light photocatalysis, and antimicrobial activity, enabling simultaneous dye degradation and pathogen removal.^493^ CuO–SiO_2_/PVA composites remove >80% of organic dyes under UV/visible irradiation while neutralizing microbial pollutants.^494^ Polysulfone/TiO_2_–NiFe_2_O_4_@SiO_2_ membranes achieve >95% antibiotic removal with magnetic recyclability.^495^ In soil systems, hybrid MOFs such as ZIF-8(Fe) demonstrate catalytic pollutant degradation and magnetic recovery.^496,497^Fig. 33 illustrates their potential in contaminated agroecosystems.^498^

**Fig. 34:**
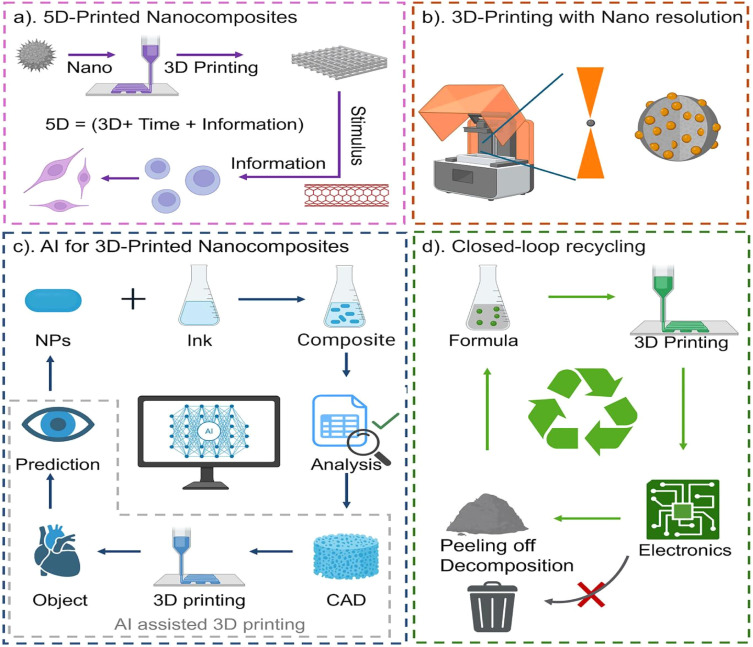
Future perspectives of nanocomposites with hybrid nanoparticles: (a) 5D-printed nanocomposites combining time and information dimensions with 3D printing; (b) nanoscale resolution in 3D-printed objects; (c) AI-assisted 3D printing and optimization; and (d) closed-loop recycling of 3D-printed nanocomposites, reproduced from ref. 3 under Creative Commons Attribution 4.0 International (CC BY 4.0) license.

Hybrid nanoparticles are reshaping 3D, 4D, and emerging 5D printing. Incorporation of nanohybrids into 3D-printed matrices enhances flexibility, thermal conductivity, and tensile strength, enabling the fabrication of customized devices. For example, liquid-crystalline elastomers with PEG-modified Au nanorods exhibit rapid photothermal heating (>150 °C s^−1^ under near-infrared), enabling shape-morphing actuators.^499^ Extending into 4D printing, such hybrids create stimuli-responsive structures for smart textiles, environmental sensors, and biomedical implants. The concept of 5D printing, integrating time and embedded information, remains nascent, but hybrid nanoparticles could serve as “information carriers” to control dynamic functions in tissue engineering and regenerative medicine. Fig. 34 summarizes these perspectives.^3,499^

## 9. Conclusion

Hybrid nanoparticle–polymer nanocomposites are transitioning from exploratory material formulations toward an architecture-driven, multifunctional materials platform in which performance is governed as much by dispersion quality, interphase engineering, and processing history as by the intrinsic properties of individual fillers. Across recent literature, three unifying insights emerge: (i) hybrid architectures can couple otherwise competing mechanisms when co-dispersion and effective interfacial stress, charge, or heat transfer are achieved; (ii) processing is not a secondary consideration but a primary determinant of microstructure, percolation behavior, and multifunctional response; and (iii) successful translation depends on integrating reproducibility, safety, and sustainability into material design rather than treating them as post-hoc constraints.

At the field level, this body of work reframes hybrid nanocomposites as systems-engineered materials, in which dispersion, interphase chemistry, and processing pathways collectively determine whether hybridization delivers reliable performance windows or inconsistent outcomes. Consequently, deployment in advanced coatings, lightweight structural components, additive manufacturing feedstocks, biomedical and food-contact materials, and energy-relevant systems will increasingly be determined by the ability to control and verify microstructure under scalable manufacturing conditions, rather than by isolated demonstrations of peak laboratory performance.

Despite substantial progress, critical bottlenecks remain. Stable co-dispersion of dissimilar nanofillers at industrially relevant loadings, preservation of hybrid architectures during high-shear or high-temperature processing, and persistent trade-offs among conductivity, toughness, processability, optical clarity, and biocompatibility continue to generate contradictory reports across the literature. These challenges are amplified by the lack of standardized metrology, limited 3D characterization of interphases, and the near absence of real-time, in-line monitoring during processing, all of which undermine reproducibility and slow scale-up.

The strength of current conclusions is further constrained by heterogeneity across polymer matrices, hybrid designs, fabrication routes, and testing protocols, as well as limited data on long-term durability, nanoparticle release, recyclability, and full life-cycle impacts. Progress will therefore depend on prioritizing measurable dispersion and interphase quality as default design variables, developing predictive structure-process-property models that explicitly incorporate hybrid morphology, embedding safety and sustainability qualification from the outset, and enabling data-driven discovery through standardized and openly accessible datasets. With these shifts, hybrid polymer nanocomposites are positioned to move from promising laboratory systems to reliable, scalable, and responsibly deployed materials.

## Author contributions

Tasnim Alam Subah: formal analysis, methodology, writing – original draft; Hasan Tasnim Salehin: data curation, investigation, writing – original draft; Rakibul Hasan Rafi: formal analysis, methodology, writing – original draft; Md. Fozle Rabbi: investigation, resources, writing – original draft; Sayem Kawsar: data curation, writing – original draft; Sumaiya Khan: resources, writing – original draft; Shahla Rahman: formal analysis, writing – original draft; Sifat Kawsar: visualization, data curation, writing – original draft; Md Zillur Rahman: conceptualization, project administration, supervision, validation, writing – original draft, writing – review & editing.

## Conflicts of interest

The authors declare that they have no known competing financial interests or personal relationships that could have appeared to influence the work reported in this paper.

## Data Availability

No primary research results, software, or code have been included, and no new data were generated or analysed as part of this review.
